# Some Theoretical and Experimental Evidence for Particularities of the Siloxane Bond

**DOI:** 10.3390/molecules27238563

**Published:** 2022-12-05

**Authors:** Alexandru-Constantin Stoica, Madalin Damoc, Corneliu Cojocaru, Alina Nicolescu, Sergiu Shova, Mihaela Dascalu, Maria Cazacu

**Affiliations:** 1 Department of Inorganic Polymers, “Petru Poni” Institute of Macromolecular Chemistry, Aleea Grigore Ghica Voda 41A, 700487 Iasi, Romania; 2 NMR Laboratory, “Petru Poni” Institute of Macromolecular Chemistry, Aleea Grigore Ghica Voda 41A, 700487 Iasi, Romania

**Keywords:** siloxane bond, Schiff base, coordination compound, silanol, tetramethyl disiloxane, aminoethylaminomethyl moiety, theoretical calculations, structural analysis

## Abstract

The specific features of the siloxane bond unify the compounds based on it into a class with its own chemistry and unique combinations of chemical and physical properties. An illustration of their chemical peculiarity is the behavior of 1,3-bis(2-aminoethylaminomethyl)tetramethyldisiloxane (AEAMDS) in the reaction with carbonyl compounds and metal salts, by which we obtain the metal complexes of the corresponding Schiff bases formed in situ. Depending on the reaction conditions, the fragmentation of this compound takes place at the siloxane bond, but, in most cases, it is in the organic moieties in the *β* position with respect to the silicon atom. The main compounds that were formed based on the moieties resulting from the splitting of this diamine were isolated and characterized from a structural point of view. Depending on the presence or not of the metal salt in the reaction mixture, these are metal complexes with organic ligands (either dangling or not dangling silanol tails), or organic compounds. Through theoretical calculations, electrons that appear in the structure of the siloxane bond in different contexts and that lead to such fragmentations have been assessed.

## 1. Introduction

**Scheme 1:**
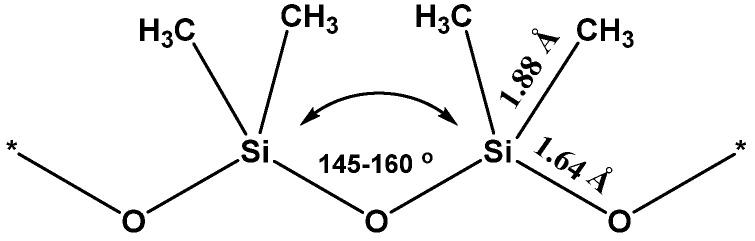
The siloxane bond in a dimethylsiloxane chain.

Compounds containing a Si-O-Si bond, with organic substituents on the silicon atoms, are the basis of silicone materials. From their discovery to the present day, these have evolved into a huge business due to their multitude of applications, premised on their unique combination of properties, such as flexibility, hydrophobicity, high chemical, thermal, and weather stability, biocompatibility, etc. Both the chemistry and the properties of these compounds differ significantly from those of their ethereal counterparts [1]. One of the most important particularities of the siloxane bond is its length, at 1.64 Å (Scheme 1), a value much smaller than that of 1.77 Å, resulting not only from the summation of the covalent radii of the two atoms involved [2] but also from the 1.71 Å that results from the corrections imposed by the difference in electronegativities (1.8 for silicon and 3.5 for oxygen, according to Pauling), which gives a partial ionic character (about 40%) to the bond.

The real and lower value of the length of the siloxane bond is also attributable to the contribution of the widely accepted character of the double bond, generated by the *pπ* → *dπ* interaction between the non-participating *p* electrons of oxygen as donors and the *3d* orbitals of silicon as acceptors. The degree of the double bond (bond order) depends on the nature of the substituents attached to the silicon and oxygen atoms [1,2,3,4]. Some investigations have even shown that both *p*(O) → *d*(Si) and *p*(O) → σ*(Si-R) interactions are present simultaneously. Consequently, both the oxygen basicity and the Si–C bonds are simultaneously weakened, so that *p*(O) → σ*(Si–R) back-bonding and the ionic contribution largely dictate the structural behavior of siloxanes, more so than in the case of organic ethers [1]. Back-bonding shortens and thereby strengthens the Si-O bond and lowers the HOMO energy, which causes the basicity and the ability to donate oxygen to other elements or to form hydrogen bonds to be diminished [1].

The ab initio calculations on the Si-O-Si bond revealed that (*p-d*)*π* bonding is of minor importance, while the ionic character is much higher than that estimated based on the difference between the electronegativities of the two atoms involved [2]. When analyzing other silicon compounds with Si-X bonds, it was found that this difference increases with the increasing electronegativity of the X atom [2]. That’s why the siloxane bond has been called the “evasive” bond [5]; a more correct representation of it uses resonance structures, although their contribution to the general structure of the Si-O bond is different [1].

The partial *π* bond that is superimposed on the *σ* bond shortens the Si-O distance, while the partial delocalization of the non-participating electron pairs of oxygen allows the increase in the Si-O-Si angle [2,6]. The value of the Si-O-Si angle varies within very wide limits, from 105° in heterocyclic compounds with C and a siloxane bond to 150–170° in hexahalogenosiloxanes, with 125° to 180° in silicates [7], but the equilibrium value for the Si-O-Si bond angle is around 145–160°. The energy required to deform this bond to 180° is very low, 1.3 kJ/mol, while, to enable deformation to lower values (toward 109°, which corresponds to *sp^3^* hybridization), much higher energies are required [8]. The quantum chemistry calculations also showed that oxygen basicity increases with a decreasing Si-O-Si angle; this means that hyperconjugation and, by implication, ionicity are lower at small angles [1]. However, the high degree of ionicity of the Si-O and Si-R bonds allowed access to a series of interesting compounds in the field of inorganic molecular chemistry and materials science [1].

One of the most important reactions leading to the formation of the siloxane bond, which is widely used in the synthesis of polysiloxanes with different architectures (cyclic, linear, silsesquioxanes, networks, etc.), is the acid-catalyzed condensation of silanol groups as the starting or intermediate species. Therefore, the mechanism of this reaction has been intensively studied, through both experimental and computational approaches. Kinetic studies have shown that in an acidic environment, the condensation of silanols consists of a set of equilibrium reactions. The formation of complexes via hydrogen bonds strongly affects the general position of the equilibrium by decreasing the concentrations of acid and silanolic and siloxane species. That is why the balance between condensation formation and the breaking of the siloxane bond is very sensitive to the donor-acceptor properties of the environment, as well as to the presence of water and other additives capable of forming strong hydrogen bonds [9,10].

The current study started from a series of experimental results conducted with the intention of obtaining salen-type metal complexes based on a reagent containing the siloxane bond, 1,3-bis(2-aminoethylaminomethyl)tetramethyldisiloxane, when a series of unexpected compounds were produced, including a stable silanol moiety. Although they were isolated and characterized from a structural point of view, including via X-ray diffraction on a single crystal, the need arose to find explanations for this diamine fragmentation in such reaction systems, namely, those for the synthesis of metal complexes of Schiff bases that are derived from different carbonyl compounds. For this purpose, a series of computational chemistry calculations were conducted.

## 2. Results

**Scheme 2:**
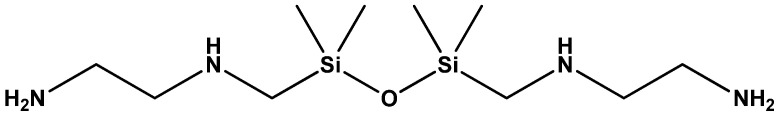
The structure of 1,3-bis(2-aminoethylaminometyl)tetrametyldisiloxane (AEAMDS).

As an alternative to 1,3-bis(3-aminopropyl)tetramethyldisiloxane (APTMDS), extensively used in recent years by ourselves to obtain salen-type Schiff bases, we decided to use another commercially available diamine, 1,3-bis(2-aminoethylaminomethyl)tetramethyldisiloxane (AEAMDS) (Scheme 2).

**Scheme 3:**
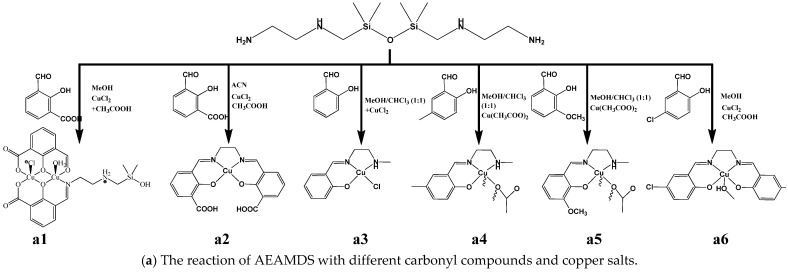

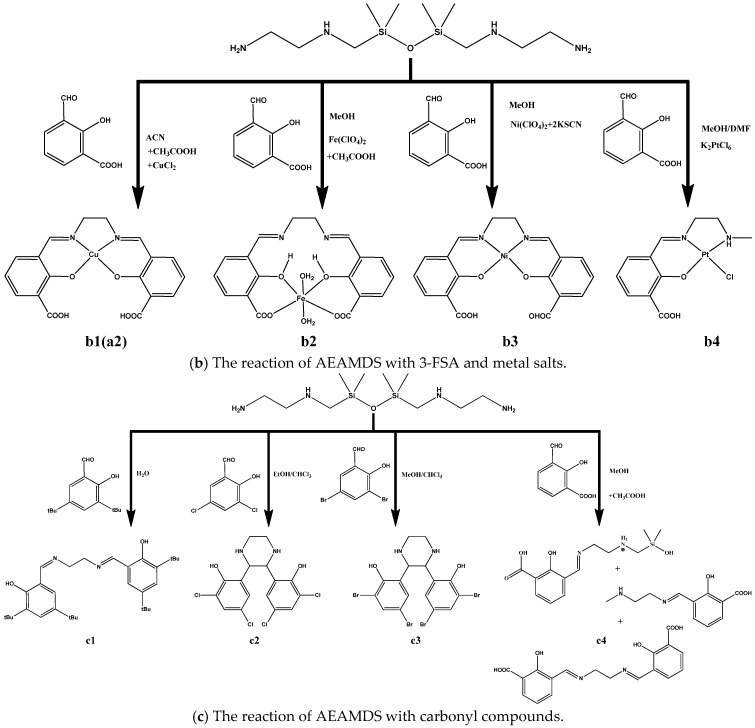
The main reactions are shown, with the involvement of AEAMDS being addressed and the main products obtained.

In a first approach, detailed in one of our previous works [11], AEAMDS was treated with 3-formylsalicylic acid (3-FSA) and copper(II) chloride in a weakly acidic medium, aiming to prepare the proper coordination compound of the salen-type ligand, formed in a one-pot procedure (Scheme 3a1). However, along with the CH=N bond formation, the Si-O-Si bond was also broken down to form the Si-OH bonds, tailing the remaining monoamine, derived Schiff base, and copper complex, respectively. In an attempt to avoid this unwanted reaction, various experiments were carried out. These consisted of the modification of the reactants, i.e., the carbonyl compound and the metal salt and their combinations, or the pH of the reaction medium, respectively, as shown in Scheme 3.

As can be seen in Scheme 3, in all cases apart from a1, the isolated products are based on ethylenediamine, which is released by the decomposition of AEAMDS. This is attributed to the lability of the secondary amine group in the *β* position, relative to the silicon atom in the disiloxane. The Fourier-Transform Infrared Spectroscopy (FTIR) monitoring of the iminization reaction between AEAMDS and 3-FSA (Scheme 3c4) revealed that, along with the formation of azomethine, the breaking of the siloxane bond occurs with the formation of silanol (Figure S1a) but also with the fragmentation of aminoethylaminomethyl with the formation of ethylenediamines (*En*). There is one exception, wherein the silicone motif is found in the product (1a) as aminoethylaminomethylsilane, which was involved in the formation of the desired azomethine; the secondary amine group is retained but the siloxane bond is broken, forming the silanol tail.

**Figure 1:**
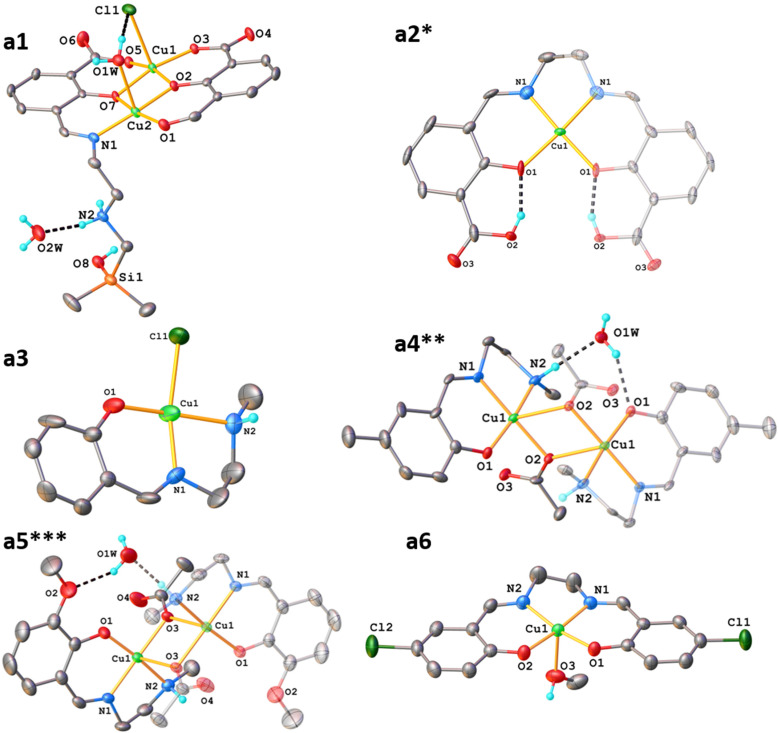
SC XRD analysis for compounds **a1**–**a6** (symmetry code: * 1 − *x*, + *y*, ½ − z, ** 1 − *x*, 2 − *y*, 1 − *z*; *** 1 − *x*, 1 − *y*, 1 − *z*). CCDC **a1**: 2064429; **a2**: 818612.

**Figure 2:**
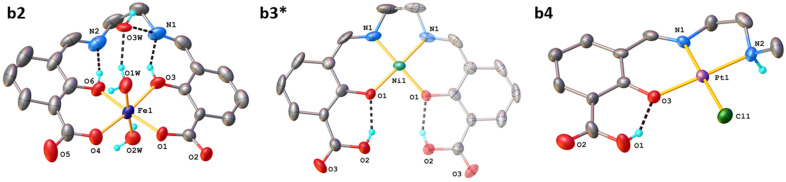
SC XRD analysis for compounds **b2**–**b4** (symmetry code: * 32 − *x*, + *y*, 1 − *z*).

**Figure 3:**
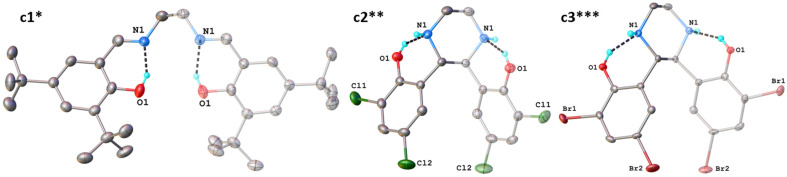
SC XRD analysis for compounds **c1**–**c3** (* 1 − x, + y, ½ − z (CCDC: 102872, 253998); ** 32 − x, + y, 1 − z; *** ½ − x, + y, 1 − z).

The formed compounds were isolated as adequate crystals for single-crystal X-ray diffraction analysis (SC XRD). Following the described procedures, new compounds (**a3**–**a6**, **b2**–**b4**, **c2**–**c3**) have been obtained, with the main crystallographic data and refining details presented in Table 1, Table 2 and Table 3, while a selection of the bond lengths and angles is provided in Tables S1–S9 of the Supplementary Materials. In addition, compounds that are already reported in the CCDC database (as **a1**, **a2**, and **c1**) were obtained. In Figure 1, Figure 2 and Figure 3 are shown the resulting structures of the isolated compounds as determined by SC XRD analysis. The FTIR spectra can be seen in Figure S1b–j.

**1.**  **Reaction products of AEAMDS, with the various carbonyl compounds and copper salts.**
**2.**  **Products of the AEAMDS reaction with 3-fsa and metal salts.**
**3.**  **Products of the AEAMDS reaction with different carbonyl compounds.**

It was concluded that AEAMDS is unstable in the reaction environment; therefore, based on the structures formed, an attempt was made to explain this instability and propose a reaction mechanism.

**Scheme 4:**
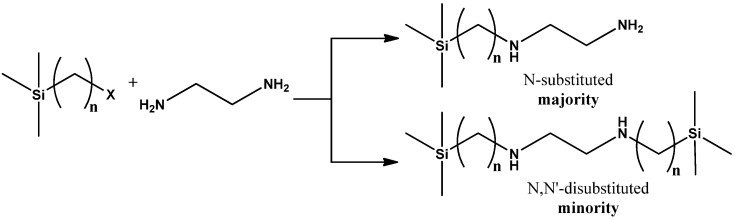
The reaction for obtaining *En* derivatives [12].

Moreover, after analyzing the literature, one can observe the difficulty of stabilizing this compound from the stage of its synthesis. The preparation of this diamine was first reported by Hu et al. in 1984 [12]. The authors report the synthesis of a series of silane and siloxane diamines, based on the reaction of ethylenediamine and alkyl-chlorinated silane or siloxane derivatives, using a three- to fourfold excess of ethylenediamine in refluxing toluene, leading to yields of 50 to 80% in N-substituted products that are contaminated only with small amounts of N,N′-disubstituted amines (Scheme 4) [12].

**Scheme 5:**
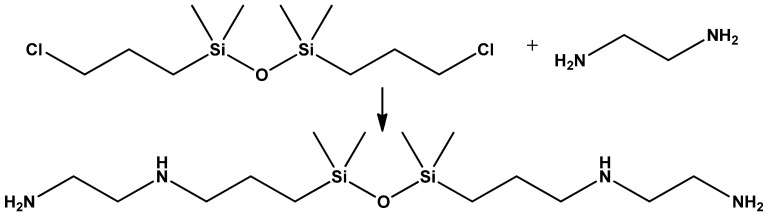
The synthesis of AEAPDS [12].

AEAPDS was prepared via the reaction of bis(chloropropyl)tetramethyldisiloxane and *En* (Scheme 5), with an excess of 1:3.

**Scheme 6:**
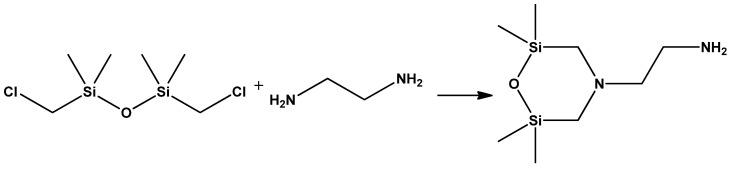
The reaction between bis(chloromethyl)tetramethyldisiloxane and *En* [12].

However, under the same conditions, the reaction of bis(chloromethyl)tetramethyldisiloxane (ClMTMDS) with ethylenediamine led to a cyclic disiloxane and traces of linear disiloxane (Scheme 6). Intramolecular cyclization then occurs, despite a 10/1 molar ratio of ethylenediamine/chloromethyldisiloxane.

**Scheme 7:**
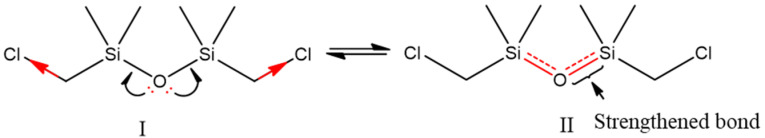
The limit structures of bis(chloromethyl)tetramethyldisiloxane.

Considering the electronic structure of the siloxane bond, one can write the following boundary structures for bis(chloromethyl)tetramethyldisiloxane, as shown in Scheme 7.

Structure **I** shows the undisturbed form of the siloxane bond. Given the electronegativity of the chlorine atoms, they will then attract electrons from the C-Si bond, thus forcing the non-participating electrons of the O atom to enter into conjugation with the *d* orbitals of the silicon atoms. Thus, the siloxane bond will acquire a partial double-bond character, which is more pronounced than in the undisturbed form. With the increase in the degree of the double bond, the siloxane bond becomes a strengthened bond, a situation in which the compound is inert in response to the electrophilic attack of boron trifluoride [3].

**Scheme 8:**
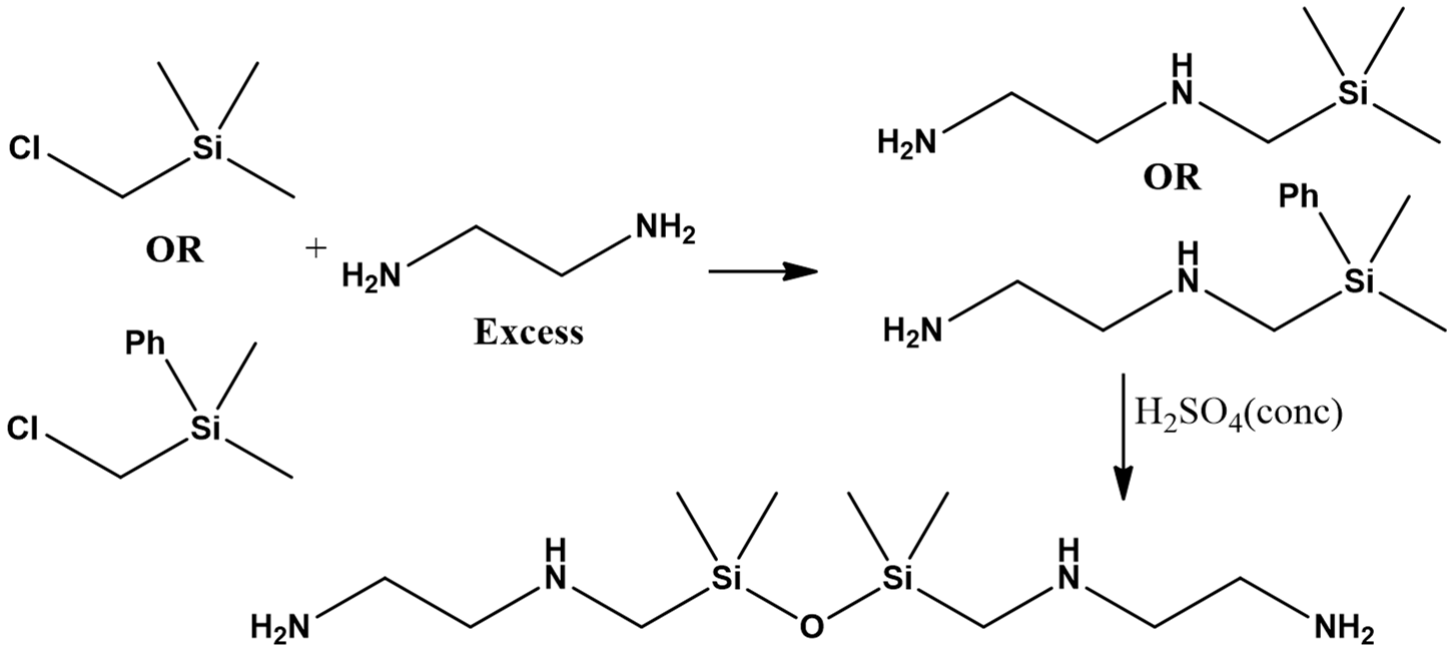
Synthesis reaction of AEAMDS [12].

AEAMDS can be prepared by acid-breaking when one mole of methane is released for each trimethylsilyl group when trimethylsilylmethylethylethylenediamine is treated with concentrated sulfuric acid. Additionally, instead of trimethylsilyl, dimethylsilylphenyl can be used, which will lead to a better yield. These results strongly suggest that the best synthetic strategy for obtaining disiloxanes and polysiloxanes containing ethylenediamine moieties is to prepare the phenylalkylsilyl derivatives of ethylenediamine, followed by the selective cleavage of phenyl groups from Si by strong acids since phenyl groups are easier to break than alkyl groups (Scheme 8) [12].

**Scheme 9:**
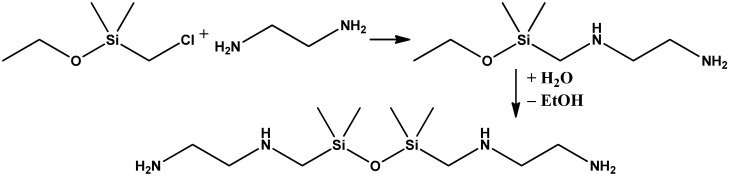
Synthesis of AEAMDS using alkoxysilane [13].

Another variant of this synthesis was reported by Li et al. in 2012, who used the reaction between *En* and chloromethylethoxydimethylsilane (2:0.5). The chlorinated derivative was slowly added dropwise to the reaction medium to ensure an excess of *En*. The compound was purified by vacuum distillation, and the obtained fraction was dissolved in distilled H_2_O to undergo the hydrolysis reaction of the alkoxy bond (Scheme 9). The product was isolated by removing the water via distillation under vacuum, the reported yield being 76% [13].

It can be observed that in any variant of the synthesis that is considered, an excess of *En* is used, on the one hand, to avoid obtaining an N,N′-disubstituted product. On the other hand, the excess of *En* has the role of shifting the chemical equilibrium toward the reaction products, while also fulfilling the role of “stabilizer”. Moreover, the compound used, as shown, has a purity of 95%, due to the content of free ethylenediamine.

**Scheme 10:**
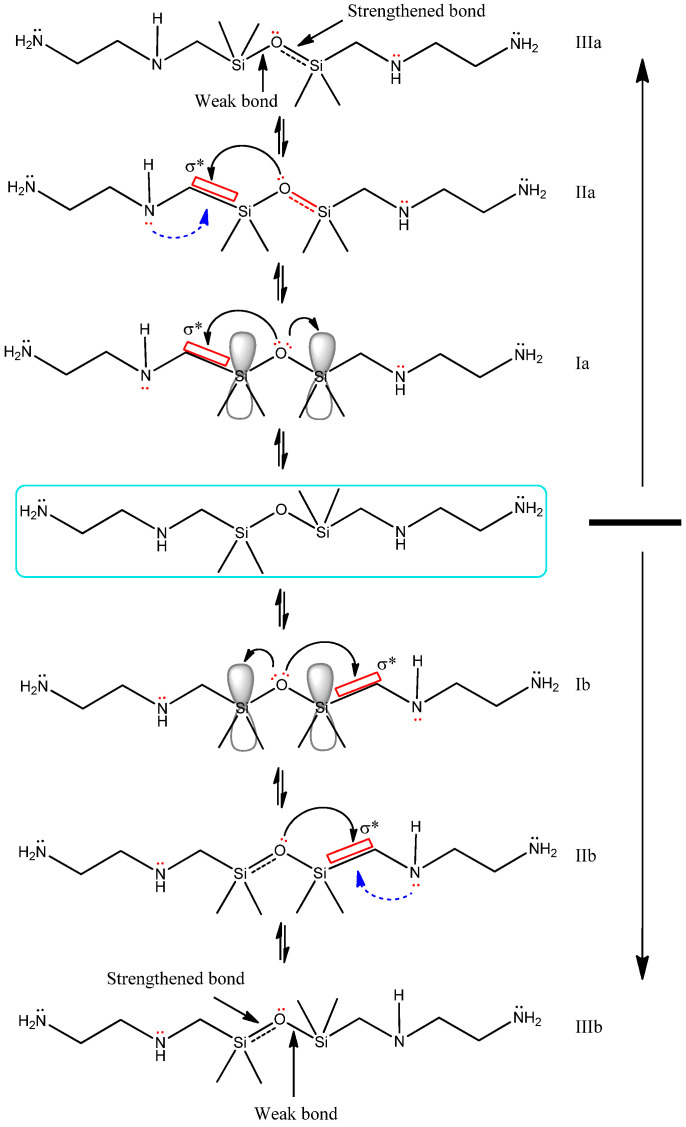
Resonance structures in the case of AEAMDS.

Returning to the iminization reaction of AEAMDS with aldehydes, the secondary amino nitrogen atom possesses a non-participating electron pair that disrupts the conjugation of the non-participating oxygen electrons with the silicon atom (Scheme 10(II)). According to Dankert and Hänisch [1], there are two types of vicinal hyperconjugation interactions in siloxanes. The first involves the 2*p* electrons of oxygen interacting with the 3*d* orbital of silicon, i.e., *p*(O) → *d*(Si). The second type involves the 2*p* electrons of oxygen interacting with the virtual molecular orbital σ*(Si-R), that is, *p*(O) → σ*(Si-R). Both types of interactions are known as back-bonding, to *d*-orbitals and *σ*-orbitals, respectively. These vicinal hyperconjugation interactions can also cause competition between the electron donation toward electrophiles and the stabilization of the Si-O bond. Previous studies have pointed out that both *p* → (Si) and *p*(O) → *(Si-R) are simultaneously present in siloxanes. In the present case, the presence of the N atom in the *β* position, i.e., in the frame of R-moiety (R = CH_2_-NH-), has an influence on the hyperconjugation interaction, *p*(O) → σ*(Si-R), which leads to the destabilizing of the Si-O bond.

**Scheme 11:**
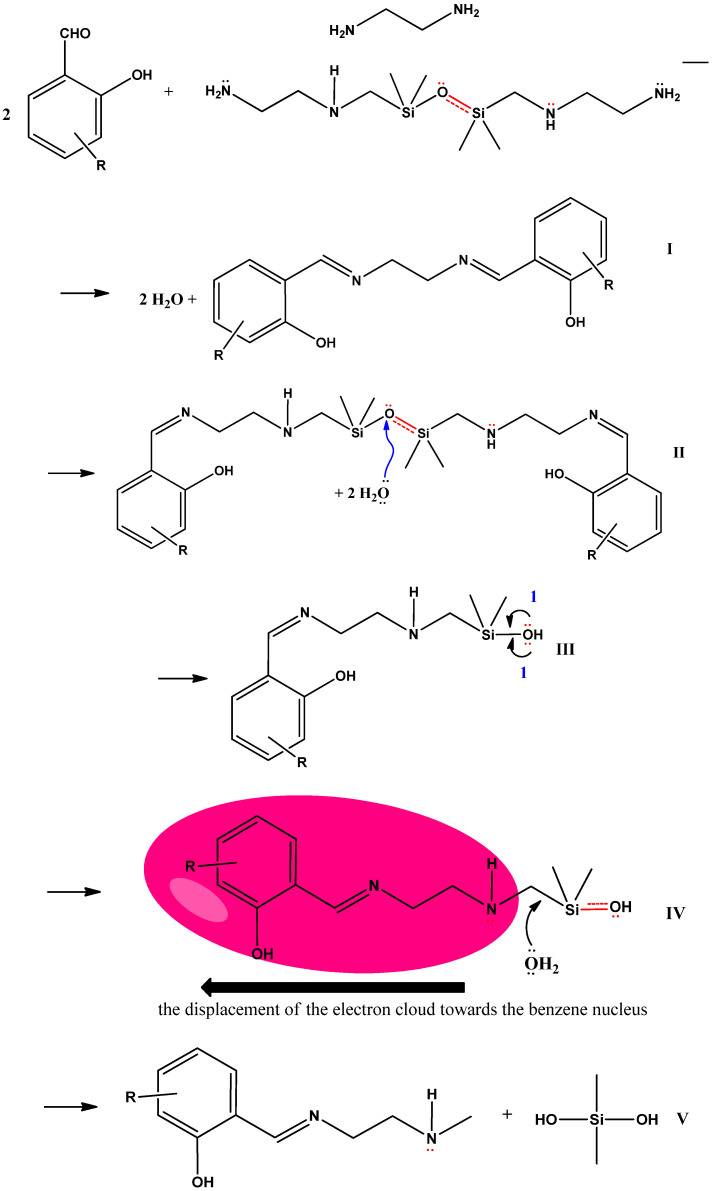
The supposed mechanism for the cleavage of the AEAMDS amine in a reaction with carbonyl compounds.

Once this process is achieved, the formation of the partial double bond between the oxygen atom and the second silicon atom occurs (Scheme 10(III)). The more pronounced double bond character gives the bond greater stability, while the decrease in low electron density weakens the Si-O bond, making it susceptible to hydrolysis. Considering the structure in Scheme 10(III) and the fact that *En* is present in the reaction medium, one can imagine the iminization reaction as follows (Scheme 11).

Given the presence of *En* in the reaction medium and its high reactivity, *En* will react first with the aldehyde, forming a bis-Schiff base. In addition, if the reaction medium is rich in H_2_O, hydrolysis of the NH-C bond with the release of *En* can also occur. From experimental observations, this phenomenon is favored by solvents with a basic character, such as acetonitrile, for example. Evidence for this process is the structures identified via X-ray single crystal diffraction analysis (*a2*, *a6*, *b2*, *b3*, *c2*, and *c3*). After the consumption of *En* from the reaction medium, iminization with the siloxane diamine will occur with the formation of the corresponding bis-Schiff base, which can undergo further reactions, as shown in Scheme 11.

**Figure 4:**
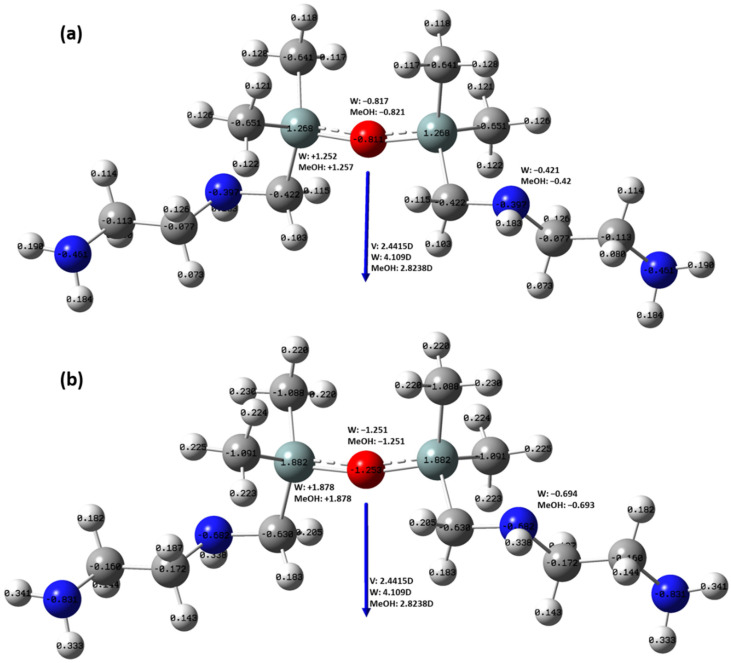
Partial charges for AEAMDS: (**a**) Mulliken, (**b**) NPA from NBO; implicit solvents: W—water, MeOH—methanol, V—vacuum.

**Figure 5:**
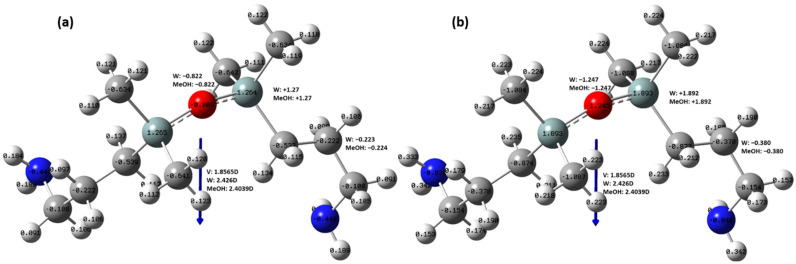
Partial charges for APTMDS: (**a**) Mulliken, (**b**) NPA from NBO; implicit solvents: W—water, MeOH—methanol, V—vacuum.

To explain these unexpected reactions, several theoretical calculations have been made. The Mulliken partial charges were compared for the optimized molecule in a vacuum, water, and methanol; the calculated data are presented in Figure 4 and Table 4. The calculation results indicate that there is an increase in the electron density of the oxygen, silicon, and nitrogen atoms. The increase in electron density at the level of the oxygen atom can be explained by the decrease in the degree of double bond between silicon and oxygen, to ensure the greater flexibility of the bond, a fact also confirmed by the decrease in the value of the Si-O-Si angle, from 164.91° (in vacuum) to 154.4° (in water) and 154.69° (in methanol). The Wiberg bond order analysis in Lowdin orthogonalized basis was made using the OUT file from the NBO analysis and the Multiwfn3.8 program [14]. The calculation highlighted the bond orders: Si-O: 1.129 in vacuum, 1.123 in water, 1.121 in methanol (Table S10). In the case of the nitrogen atom, the increase in electron density can be explained by a polarization of the molecule, a process induced by the solvent. One proof of this aspect is the fact that, in each case, the *β* position, compared to the siloxane bond, is enriched in electrons. The increase in electron density at the secondary amino nitrogen atom can also be explained by the inductive effect generated by the aminoethyl moiety. The same phenomenon was also highlighted using NPA partial charges calculated with NBO, the second-order perturbation theory analysis of the Fock matrix on the NBO basis (Table S11, Figure S5). To validate this hypothesis, the partial charges were compared with those of other siloxane derivatives that were differently substituted in the *β* position. Thus, for the 1,3-bis(3-aminopropyl)tetramethyldisiloxane (APTMDS) molecule optimized under the same conditions (Figure 5), the Mulliken partial charges determined for the oxygen atom were −0.809 in a vacuum and −0.82 in water and methanol (Table 5), the increase in electron density being due to the same phenomenon. In this case, a relaxation of the Si-O-Si angle, 160.52° (in vacuum), 153.2° (in water), and 153.4° (in methanol), was also found. Regarding the silicon atom, a decrease in electron density was observed (Mulliken partial charges) from +1.265 in a vacuum to +1.270 in water and methanol. The decrease in electron density at the silicon atom can be explained by the decrease in the degree of the double bond between silicon and oxygen, with the Wiberg bond order of Si-O being: 1.129 (in vacuum), 1.1201 (in water), 1.1206 (in methanol) (Table S12). Using the NPA, which is much more restricted at the atom level, in the case of APTMDS, no significant changes are observed at the silicon atom level, compared to AEAMDS, where the increase in electron density is observed, when the data of the second-order perturbation theory analysis of the Fock matrix in the NBO basis are analyzed (Table S13, Figure S6).

**Figure 6:**
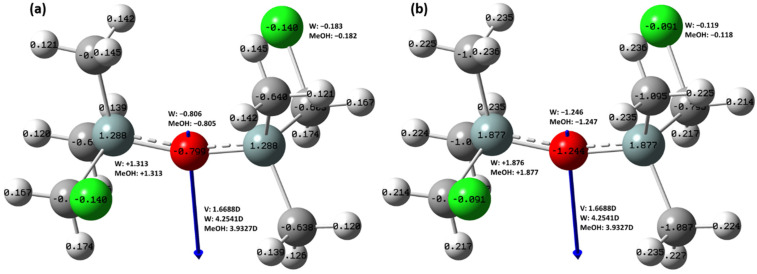
Partial charges for ClMTMDS: (**a**) Mulliken, (**b**) NPA from NBO; implicit solvents: W—water, MeOH—methanol, V—vacuum.

For a better understanding of the phenomenon, the data obtained using the AEAMDS molecules were also compared with those for 1,3-bis(chloromethyl)tetramethyldisiloxane (ClMTMDS). Thus, for ClMTMDS (Figure 6), the Mulliken partial charges for the oxygen atom were determined: −0.799 (vacuum), −0.806 (water), and −0.805 (methanol) (Table 5). The increase in electron density at the level of the oxygen atom can be explained by the decrease in the degree of the double bond between silicon and oxygen, the Si-O Wiberg bond order being: 1.14 in vacuum, 1.136 in water, and 1.137 in methanol (Table S14). In this case, a decrease in the electron density at the level of the silicon atom is also observed, from +1.288 (vacuum) to +1.313 (water and methanol). The observations in the case of NPA for ClMTMDS are similar to those obtained for APTMDS (Table S15, Figure S7).

Following the calculations, it was observed that it is only in the AEAMDS molecule that there is an increase in the electron density at the silicon atom, a fact that confirms the hypothesis that there is a migration of electrons from the nitrogen to the silicon atom (Scheme 10). NMR studies (Figure S2a–h) showed the AEAMDS degradation over time, under normal humidity conditions. Thus, in the ^1^H NMR spectrum of AEAMDS that was recorded immediately after the recipient was opened for the first time, we observed five well-resolved signals, as can be seen in Figure S2a. Based on the chemical shift values and coupling patterns, we assigned these signals to: Si-CH_3_ at 0.1 ppm (singlet), amine protons at 1.5 ppm (broad singlet, most probably due to the exchange with water), Si-CH_2_ at 2.0 ppm (singlet), and the aminoethyl group at 2.7 and 2.8 ppm (two triples). After two years, when we needed to reanalyze the same compound, kept in the original recipient, the obtained proton spectrum had completely changed (Figure S2b). Several signals were observed in the interval from −0.1 to 0.1 ppm, characteristic of Si-CH_3_ groups, and two singlets appeared around 2.0 ppm, where the Si-CH_2_ groups were previously assigned. A new singlet was visible at 2.2 ppm and several triplets were present in the interval at 2.4–2.6 ppm, the region where the aminoethyl group resonates. This complex pattern indicated the presence of several compounds, most probably the degradation products of AEAMDS. Several bidimensional homo- and heteronuclear NMR experiments were recorded (including H,H-COSY, ^1^H,^13^C-HSQC, ^1^H,^13^C-HMBC, and ^1^H,^15^N-HMBC, as Figure S2b–h), in order to gain more information about the chemical structures of these degradation products. After NMR data analysis, we identified the presence of at least five different compounds: initial AEAMDS, the silanol derivative obtained after the cleavage of the siloxane bond, aminoethylaminomethyl, ethylendiamine, and linear or cyclic dimethylsiloxane. The presence of dimethylsiloxane derivatives was indicated by the three signals from −20 to −22 ppm, visible in the ^29^Si NMR spectrum. Degradation occurs, most likely by trapping water molecules from the atmosphere. This aspect indicates that the molecule splits in the presence of water, which is why we resorted to the calculation of silanol molecules, in the form of protonated silanol.

**Figure 7:**
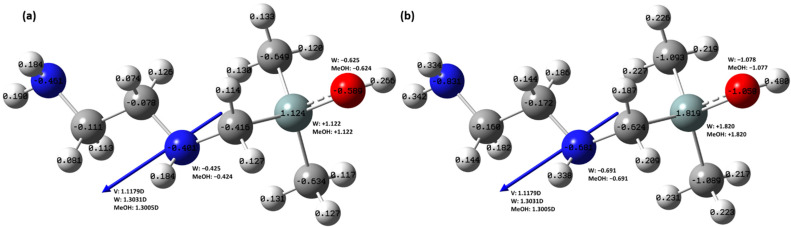
Partial charges for silanol: (**a**) Mulliken, (**b**) NPA from NBO; implicit solvents: W—water, MeOH—methanol, V—vacuum.

In the case of the silanol molecule (Figure 7), the calculations led to increased Mulliken partial charges for the oxygen: −0.589 (vacuum), −0.625 (water), and −0.624 (methanol); for the silicon atom, these were: +1.124 (vacuum) and +1.122 (water and methanol), while the Wiberg bond order of Si-O was: 1.165 in vacuum, 1.154 in water, 1.154 in methanol (Table S16). Even if, in this case (i.e., silanol), the interaction is significantly lower, the same variation pattern as in the AEAMDS molecule is observed, which confirms the hypothesis that the electron pair of the nitrogen atom influences the Si-O bond. A similar trend phenomenon is observed in the case of the NPA data (Table S17, Figure S8). The HOMO LUMO images are shown in Figure S3. 

**Figure 8:**
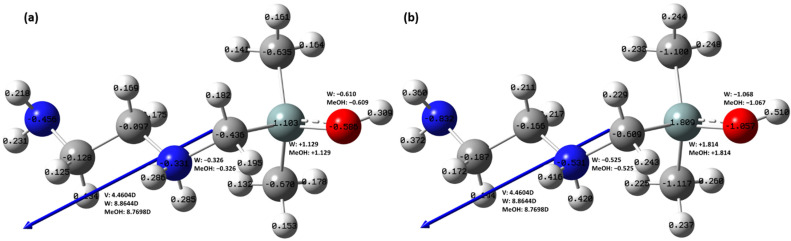
Partial charges for H-silanol: (**a**) Mulliken, (**b**) NPA from NBO; implicit solvents: W—water, MeOH—methanol, V—vacuum.

However, to verify, once again, the hypothesis according to which there is an interaction between the *π* electrons of the nitrogen atom and the siloxane bond resonance, we resorted to protonation of the nitrogen atom of the secondary amine. By protonating the atom, the electron pair is blocked, which means that it will no longer be able to interact with the silicon atom. The calculations revealed that the Mulliken partial charges for the oxygen atom increased, from −0.586 (vacuum) to −0.61 (water) and −0.609 (methanol). The calculations also show a decrease in the electron density (Mulliken partial charges) on the silicon atom, from +1.103 (vacuum) to +1.129 (water and methanol) (Figure 8). Considering the blocking of the electron pair (in the case of the protonated silanol molecule), the results of the calculations on this molecule would indicate a loss of electron density, thus strengthening the hypothesis mentioned above; the Si-O Wiberg bond order is: 1.221 in vacuum, 1.180 in water, 1.181 in methanol (Table S18). The NBO analysis data (Table S19, Figure S9) leads us to the same conclusions. The HOMO LUMO images are shown in Figure S4. 

**Figure 9:**
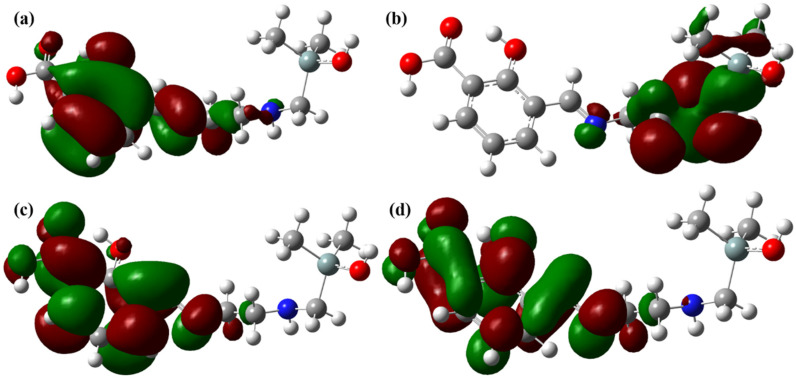
(**a**) HOMO−1, (**b**) HOMO, (**c**) LUMO, (**d**) LUMO + 1 images, for the mono-Schiff base (SB-silanol) showing the silanol fragment.

The proposed mechanism foresees the symmetric breaking of the AEAMDS molecule and the formation of a mono-Schiff base showing a silanol tail (SB-silanol). In this structure, the electron cloud can be delocalized toward the aromatic nucleus, favoring the weakening of the Si-C bond in the hydrocarbon radical. The presence of delocalization was highlighted by the HOMO-1, HOMO, LUMO, and LUMO + 1 images (Figure 9). Here, it was identified as an intramolecular charge transfer (CT). 

**Figure 10:**
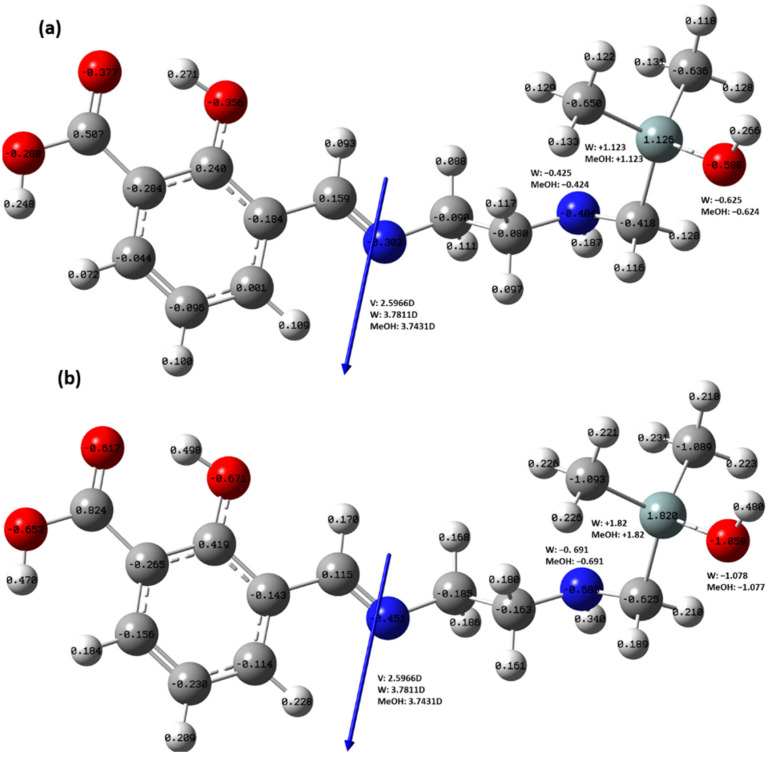
Partial charges for SB-silanol: (**a**) Mulliken, (**b**) NPA from NBO; implicit solvents: W—water, MeOH—methanol, V—vacuum.

The calculations indicate that in this case, too, there is an interaction between the nitrogen atom and the silicon atom, the Mulliken partial charges found for the silicon atom being: +1.126 (in vacuum) and +1.123 (in water and methanol) (Figure 10). The HOMO and LUMO structures support the statements in the proposed mechanism. The Wiberg Si-O bond order is: 1.166 in vacuum, 1.154 in water, 1.155 in methanol, while for the other bonds, these values are presented in Table S20. The results of the NBO calculation are presented in Table S21, Figure S10. 

**Scheme 12:**
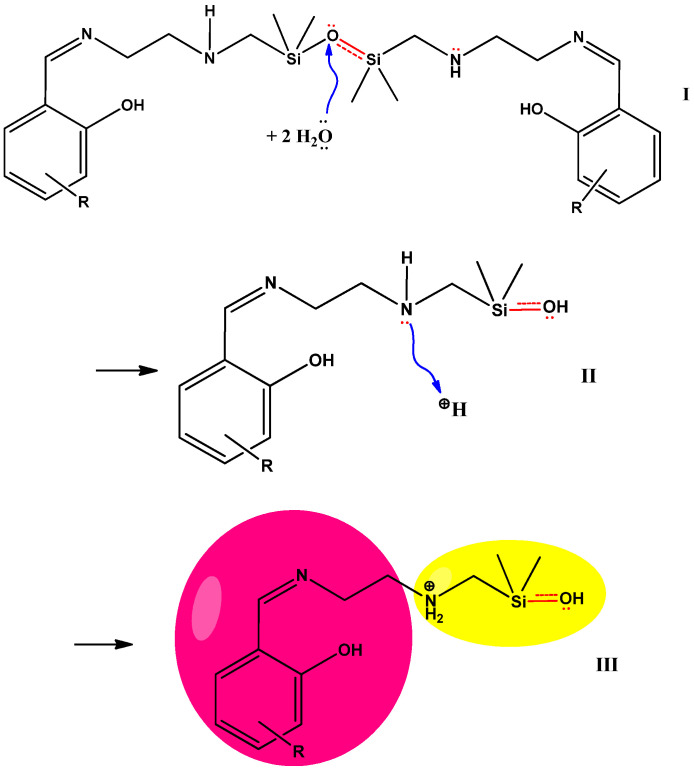
The proposed reaction mechanism in an acid medium.

**Figure 11:**
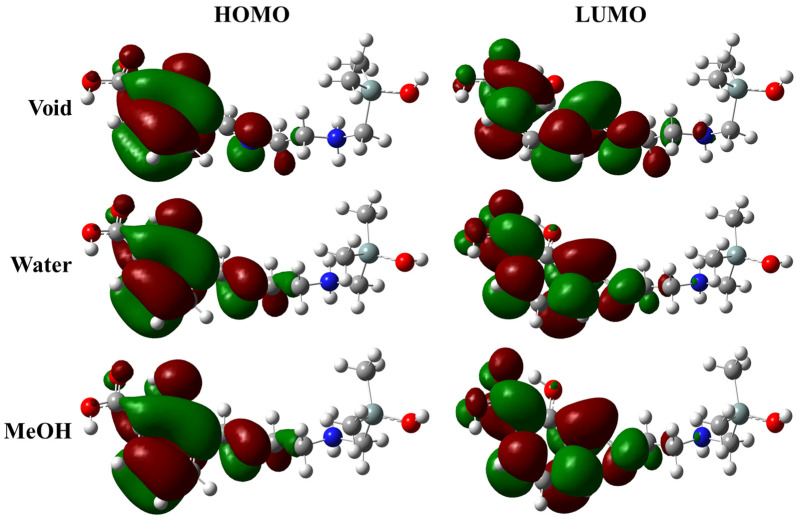
HOMO and LUMO images for the optimized SB-H-silanol molecule in vacuum, water, and methanol.

According to the proposed mechanism (Scheme 12) via protonation of the mono-Schiff base (SB-H-Silanol), at the secondary amino nitrogen, two electronic regions are formed, thus stabilizing the silanol bond. The HOMO and LUMO images (Figure 11) highlight the fact that there is a separation of the regions, according to the proposed mechanism.

**Figure 12:**
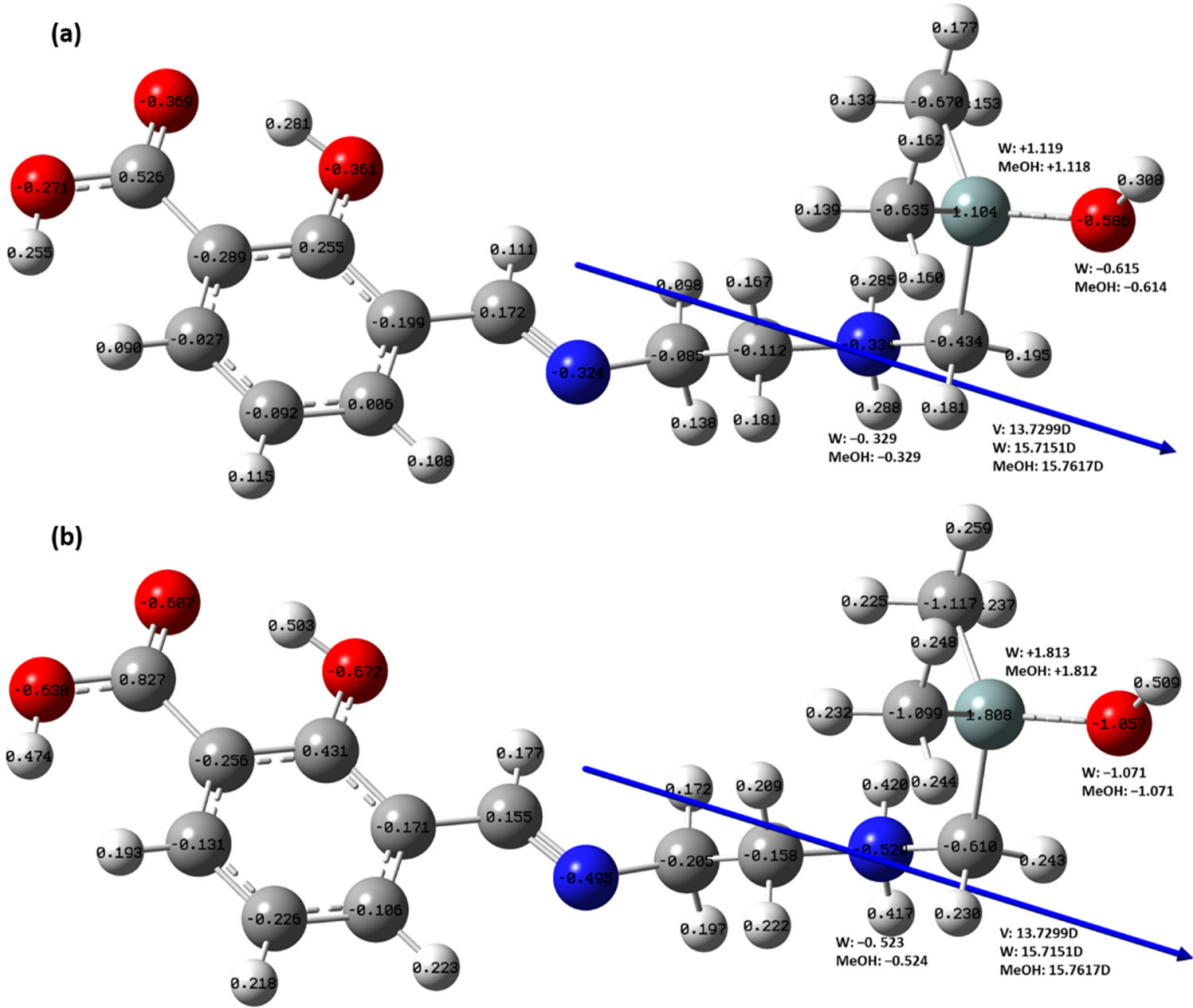
Partial charges for SB-H-silanol (**a**) Mulliken, (**b**) NPA from NBO; implicit solvents: W—water, MeOH—methanol, V—vacuum (c4).

In this case, it can also be observed that by protonating the secondary amino nitrogen atom, the interaction between nitrogen and silicon disappears. Thus, for the silicon atom, the Mulliken partial charges were found, as follows: +1.104 (vacuum), +1.119 (water), and +1.118 (methanol) (Figure 12). The Wiberg bond order of Si-O is: 1.220 (vacuum), 1.181 (water), and 1.182 (methanol) (Tables S22 and S23, Figures S11 and S12). In this case, the same phenomenon is observed as in the case of the non-iminized fragment, in the sense that the partial charge of the oxygen atom increases and the partial charge of the silicon atom decreases. This phenomenon is also valid in the case of APTMDS. The calculated data further support the hypothesis presented in the mechanism, namely, that the pair of π electrons of the nitrogen atom disturbs the Si-O-Si resonance bond, an interaction that leads to the instability of the siloxane bond and its easy hydrolysis. 

Having an amino, basic group, this silanol is self-stabilizing. This is an interesting finding, considering the context in which it is known that the stability of silanols is low as they tend to condense immediately with another silanol group or with alkoxysilanes that are present in the environment; this constitutes a major problem in terms of the lifetime of the hydrolysates of alkoxysilanes. The protonation of silanol makes the silicon more electrophilic and, thus, more susceptible to nucleophilic attack. Under basic conditions, the condensation reaction involves the attack of nucleophilic deprotonated silanol on a neutral silicate species [15]. Various methods have been applied to stabilize them, such as reducing the acid content in the silanol compound (acid added or formed in the hydrolysis process), hydrolysis at the water-organic solvent interface, hydrolysis of the alkoxysilane emulsion in water, filtering the mixture through diatomite, and the use of neutralizing agents, for example, ammonium carbonate or calcium carbonate [16]. 

## 3. Materials and Methods

### 3.1. Materials

The list of materials used in the study is as follows: 1,3-Bis(2-aminoethylaminomethyl)tetramethyldisiloxane, AEAMDS (Gelest, 11 East Steel Road, Morrisville, PA 19067, USA), C_10_H_30_N_4_OSi_2_; 95% (the only one available on the market); 278.55 g/mol; density: 0.941 g/cm^3^; bp: 140 − 5°/2; hydrolytic sensitivity: 2 (reacts with aqueous acid). 3-Formylsalicylic acid (3-FSA), C_8_H_6_O_4_, 166.131 g/mol (Polivalent-95, ap.(of.) 6, 92 str. Ismail mun. Chisinau, Moldova). The other aldehydes and metal salts used in the experimental trials were purchased from Sigma-Aldrich, Darmstadt, Germany. Salicylaldehyde, C_7_H_6_O_2_, 122.12 g/mol (Merck). 5-Methylsalicylaldehyde, C_8_H_8_O_2_, 97%, 136.15 g/mol (Sigma-Aldrich). *o*-vaniline, C_8_H_8_O, 99%, 152.15 g/mol (Aldrich). 5-chlorosalicylaldehyde, C_7_H_5_ClO_2_, 156.57 g/mol (Alfa Aesar, Erlenbachweg 2 76870, Kandel, Germany). 3,5-Di-tert-butylsalicylic acid hydrate, C_15_H_22_O_3_, 234.33 g/mol (Aldrich). 3,5-Dichlorosalicylaldehyde, C_7_H_4_Cl_2_O_2_, 99%, 191.01 g/mol (Aldrich). and 3,5-Dibromsalicylaldehyde, C_7_H_4_Br_2_O_2_, 279.91 g/mol (Acros Organics, Geel—Belgium).

### 3.2. Measurements

*Fourier transform infrared (FTIR)* spectra in transmission mode (400–4000 cm^−1^ spectral range, 2 cm^−1^ resolution, with the accumulation of 32 scans, at room temperature) were recorded on a Bruker Vertex 70 FT-IR spectrometer. *The NMR* analyses were performed on Bruker Avance Neo spectrometers, operating at 600.1, 150.9, 60.8, and 79.5 MHz for ^1^H, ^13^C, ^15^N, and ^29^Si, respectively. The 1D and 2D spectra were recorded with either a 5 mm multinuclear inverse detection z-gradient probe or a 5 mm four-nuclei (H,C,F,Si) direct detection z-gradient probe. Two-dimensional NMR homo- and heteronuclear correlations (H,H-COSY correlated spectroscopy), ^1^H,^13^C-HSQC (heternuclear single quantum coherence spectroscopy), and ^1^H,^13^C and ^1^H,^15^N-HMBC (heternuclear multiple bond correlation) experiments were recorded using standard pulse sequences in the version with z-gradients, as delivered by Bruker with a TopSpin 4.0.8 spectrometer control and processing software. The ^15^N chemical shifts were obtained as projections from the 2D indirectly detected spectra. *The crystallographic analysis* was performed using an Oxford Diffraction CCD diffractometer, XCALIBUR E, with graphite-monochromated Mo-Kα radiation. The CrysAlis package from Oxford Diffraction was used for unit cell determination and data integration [17], while structure-solving was performed using direct methods based on the Olex2 software [18], with the SHELXS structure solution program. These were refined by full-matrix least squares analysis on *F*^2^ with SHELXL-97 [19], using an anisotropic model for the non-hydrogen atoms. All H atoms were introduced in idealized positions (d_CH_ = 0.96 Å). The crystal structure of b2 contains large areas filled with strongly disordered water molecules that could not be modeled using atomic sites; therefore, the Mask option in Olex2 was used to model the contribution of disordered solvent molecules to the structure factors. For calculations with Mask routines, we used a probe radius of 1.2 A and a resolution of 0.2 A. Table 1, Table 2 and Table 3 give the main crystallographic data with refining details, while in Tables S1–S9, a selection of the bond lengths and angles is provided (CCDC: 2214011; 2214190; 2214208; 2214232; 2214239; 2214267; 2214276; 2214287; 2214292). These data can be obtained free of charge via www.ccdc.cam.ac.uk/conts/retrieving.html (accessed on 21 October 2022) (or from the Cambridge Crystallographic Data Centre, 12 Union Road, Cambridge CB2 1EZ, UK; fax: (+44) 1223-336-033; or deposit@ccdc.ca.ac.uk). *Theoretical calculations* were performed using the GAUSSIAN 09 programs. The optimization of the reactants and products of every step along the possible pathway was carried out by using the density functional theory (DFT) method, known as B3LYP, with a 6-311G(d,p) basis set (in its ground state and using the IEFPCM model with water and methanol solvent). Based on the optimized structures (vacuum, water, and methanol), the NBO calculation was performed. The NPA partial charges from NBO were compared with the Mulliken partial charges. Based on the OUT file from NBO, the Wiberg bond order was calculated, using the Multiwfn 3.8 program. All studied structures were optimized their XYZ matrices, being given in Table S24, atom labels Figures S5–S12 and electrostatic potential surface for SB-Silanol Figure S13.

### 3.3. Syntheses

#### 3.3.1. The Reaction of AEAMDS with Different Carbonyl Compounds and Copper Salts

##### Synthesis, Leading to Compound **a1**

A solution consisting of 60 mg (0.2 mmol) AEAMDS, dissolved in 5 mL methanol, was added dropwise over a solution containing 83 mg (0.50 mmol) of 3-FSA, dissolved in 15 mL MeOH at room temperature (RT); the resulting mixture immediately turned pale yellow. The mixture was stirred vigorously for 10 minutes at RT, after which a catalytic amount of glacial AcAc acid was added, and the temperature was raised to 60 °C and maintained for 2 h. Then, a solution consisting of 134 mg (0.78 mmol) of CuCl_2_ × 2H_2_O, dissolved in 10 mL of MeOH, was added dropwise, after which the color of the reaction mass immediately turned emerald green. The reaction mixture was refluxed for 1 h, after which it was filtered, and the filtrate was allowed to stand at RT. After about a week, emerald green cubic crystals were formed, which were separated by filtration, washed quickly with methylene chloride, dried, and analyzed.

##### Synthesis Leading to Compound **a2**(**b1**)

A solution consisting of 60 mg (0.2 mmol) AEAMDS, dissolved in 5 mL acetonitrile, was added dropwise over a solution containing 83 mg (0.50 mmol) of 3-FSA, dissolved in 10 mL acetonitrile at RT. The resulting mixture immediately turned pale yellow. The mixture was stirred vigorously for 10 minutes at RT, after which a catalytic amount of glacial acetic acid was added, and the temperature was raised to 60 °C and maintained for 2 h. Then, a solution consisting of 68 mg (0.4 mmol) of CuCl_2_ × 2H_2_O, dissolved in 5 mL of acetonitrile, was added dropwise, when the color of the reaction mass immediately turned brown. The reaction mixture was refluxed for 1 h, after which it was filtered, and the filtrate was allowed to stand at RT. After about a week, red-brown rectangular prism crystals were formed, which were separated by filtration, washed quickly with methylene chloride, dried, and analyzed.

##### Synthesis Leading to Compound **a3**

In a round-bottomed flask, 0.27855 g (1 mmol) of AEAMDS, dissolved in 5 mL of MeOH, were introduced, over this solution, a solution formed by dissolving 0.244 g (2 mmol) of salicylaldehyde was added, in 5 mL of chloroform. The reaction mixture was stirred vigorously at RT and then placed at 65 °C for 2 h; after that, the flask was removed from the bath and a solution of CuCl_2_ (0.134 g (1 mmol), dissolved in 2 mL MeOH), was added over the mixture, then the mixture was stirred for a further 30 min. At this point, a dark green solution formed. The mixture was then allowed to stand for crystallization.

##### Synthesis Leading to Compound **a4**

In a round-bottomed flask, 0.2799 g (1 mmol) of AEAMDS dissolved in 5 mL of MeOH were introduced; over this, a solution formed by the dissolving of 0.272 g (2 mmol) of 5-methylsalicylaldehyde in 5 mL of chloroform was added. The reaction mixture was stirred vigorously at RT and then heated at 65 °C for 2 h. After that, the flask was removed from the bath, and a solution of Cu(AcO)_2_ (0.183 g (1 mmol) dissolved in 2 mL MeOH) was added over the mixture, and the mixture was stirred for a further 30 min. The mixture was then allowed to stand for crystallization.

##### Synthesis Leading to Compound **a5**

In a round-bottomed flask, 0.2799 (1 mmol) g of AEAMDS, dissolved in 5 mL of MeOH, were introduced; over this solution, we added a solution formed by dissolving 0.3043 g (2 mmol) of *o*-vanillin in 5 mL of chloroform. The reaction mixture was stirred vigorously at RT, and then placed at 65 °C for 2 h. After that, the flask was removed from the bath, and a solution of Cu(AcO)_2_ (0.183 g (1 mmol) dissolved in 2 mL MeOH) was added over the mixture, and the mixture was stirred for a further 30 min. The mixture was then allowed to stand for crystallization.

##### Synthesis Leading to Compound **a6**

In a round-bottomed flask, 0.0314 g (0.2 mmol) of 5-chlorosalicylaldehyde, dissolved in 10 mL MeOH, and 0.0295 mL (0.1 mmol) of AEAMDS, dissolved in 2 mL MeOH, were introduced. The mixture was left under stirring for 1 h. Subsequently, a solution consisting of 0.034 g (0.2 mmol) CuCl_2_, dissolved in 3 mL MeOH, was added. The reaction mixture changed color from straw yellow to blue. The mixture was filtered, then 200 µL AcAc was added and allowed to crystallize.

#### 3.3.2. The Reaction of AEAMDS with 3-FSA and Metal Salts

##### Synthesis Leading to Compound **b2**

In a round-bottomed flask, 0.1016 g (0.4 mmol) Fe(ClO_4_)_2_, dissolved in 2 mL MeOH, and 0.0664 g (0.2 mmol) 3-FSA, dissolved in 10 mL MeOH, were introduced. First, 200 µL of AcAc was added to this mixture. The mixture was vigorously stirred at RT for 1 h. After this, a solution formed by dissolving 0.0295 mL (0.1 mmol) of AEAMDS in 2 mL MeOH was added. Upon the addition of AEAMDS, the color of the reaction mixture changed from straw yellow to reddish brown. The mixture was vigorously stirred for 2 h, then filtered and left to crystallize.

##### Synthesis Leading to Compound **b3**

In a round-bottomed flask, 0.0664 g (0.2 mmol) of 3-FSA, dissolved in 10 mL of MeOH, was introduced; over this solution, we added a solution formed by dissolving 0.0295 mL (0.1 mmol) of AEAMDS in 2 mL of MeOH. A solution formed by dissolving 0.0194 g (0.2 mmol) KSCN in 10 mL MeOH, and a solution formed by dissolving 0.0365 g (0.1 mmol) of Ni(ClO_4_)_2_ in 10 mL MeOH, were combined separately. The resulting solution was filtered and added over the Schiff base solution. The mixture was stirred for 2 h, then left to crystallize. Needle-shaped crystals of a reddish color were then formed.

##### Synthesis Leading to Compound **b4**

In a round-bottomed flask, 60 µL (0.1 mmol) of AEAMDS, dissolved in 5 mL of MeOH, were introduced; over this solution, we added a solution formed by dissolving 0.0664 g (0.2 mmol) of 3-FSA in 5 mL of chloroform. The reaction mixture was stirred vigorously at RT, then heated at 65 °C for 2 h, after which the solvent was completely removed via rotavapor. An oily solid then formed. Subsequently, 0.0295 g of SB dissolved in 5 mL of MeOH was used, over which was added a solution consisting of 0.0415 g (0.1 mmol) of K_2_PtCl_6_, dissolved in a solvent mixture of MeOH: DMF (*v*:*v*. 3:2). The mixture was refluxed for 4 h, after which it was left to crystallize.

#### 3.3.3. Reaction of AEAMDS with Carbonyl Compounds

##### Synthesis Leading to Compound **c1**

In a round-bottomed flask, 0.1 g (4.3 mmol) of 3,5-di-tert-butylsalicylaldehyde, dissolved in 25 mL of water, was introduced; then, separately, a solution of AEAMDS was prepared by dissolving 0.631 mL (0.21 mmol) of AEAMDS in 5 mL of water. The AEAMDS solution was added over that of the aldehyde, and the mixture was then refluxed for 3 h. Finally, the mixture was left to stand at RT.

##### Synthesis Leading to Compound **c2**

In a round-bottomed flask, 0.2799 g (1 mmol) of AEAMDS, dissolved in 5 mL of MeOH, was introduced; over this solution, we added a solution formed by dissolving 0.56 g (2 mmol) of 3,5-dibromosalicylaldehyde in 5 mL of CHCl_3_. The mixture was refluxed for 3 h and then allowed to stand at RT for crystallization.

##### Synthesis Leading to Compound **c3**

In a round-bottomed flask, 0.1 g (0.52 mmol) of 3,5-dichlorosalicylaldehyde was dissolved in 10 mL of ethanol, after which, 84 μL (0.28 mmol) of AEAMDS dissolved in 5 mL CHCl_3_. The mixture was refluxed for 2 h, until the formation of a yellow solution. The reaction mixture was then subjected to the slow evaporation of the solvent, when yellow crystals were formed.

##### Synthesis Leading to Compound **c4**

In a round-bottomed flask, 0.0166 g (0.1 mmol) of 3-FSA, dissolved in 5 mL MeOH, was introduced, after which, 29.5 μL (0.05 mmol) of AEAMDS dissolved in 2 mL MeOH was added, until finally, 11.5 μL of AcAc acid was added. The mixture was stirred at RT for 2 h, until the formation of a yellow solution. The reaction mixture underwent the slow evaporation of the solvent to yield a sticky yellow solid.

## 4. Conclusions

The experimental facts indicate, as supported by the theoretical calculations, that the siloxane bond undergoes hybridization processes that are influenced by the electron-donating atoms in the *β* position from the silicon atoms and by the experimental conditions, which leads to the fragmentation of the molecule, along with the obtaining of a mixture of compounds.

## Figures and Tables

**Table 1:** Crystal data and details of data collection for the ***a*** compounds.

Compound	a3	a4	a5	a6
Empirical formula	C_10_H_13_ClCuN_2_O	C_15_H_25_CuN_3_O_4_	C_13_H_20_CuN_2_O_5_	C_17_H_16_Cl_2_CuN_2_O_3_
Fw	276.21	374.92	347.85	430.76
Space group	*Pnma*	*Pbcn*	*Pbca*	*P-1*
*a* [Å]	10.0886 (9)	16.2686 (14)	8.8802 (5)	8.3005 (7)
*b* [Å]	6.4401 (14)	8.5991 (13)	16.4645 (7)	9.7813 (9)
*c* [Å]	17.0497 (16)	23.510 (3)	21.3421 (12)	11.6182 (13)
α [°]	90	90	90	67.828 (9)
β [°]	90	90	90	83.039 (8)
γ [°]	90	90	90	81.165 (7)
*V* [Å^3^]	1107.7 (3)	3288.9 (7)	3120.4 (3)	861.10 (15)
*Z*	4	8	8	2
*ρ*_calc_ [g cm^−3^]	1.656	1.514	1.481	1.661
Crystal size [mm^3^]	0.10 × 0.08 × 0.02	0.15 × 0.10 × 0.02	0.40 × 0.30 × 0.25	0.30 × 0.20 × 0.05
T [K]	180.00 (14)	180.0 (8)	293 (2)	293 (2)
*μ* [mm^−1^]	2.186	1.352	1.421	1.598
2Θ range	4.692 to 50.034	3.464 to 50.05	4.948 to 50.05	3.794 to 50.042
Reflections collected	5124	7316	7552	5902
Independent reflections	1071 [*R*_int_ = 0.0647]	2863 [*R*_int_ = 0.0989]	2758 [*R*_int_ = 0.0318]	3045 [*R*_int_ = 0.0240]
Data/restraints/parameters	1071/10/124	2863/19/199	2758/0/195	3045/3/230
GOF	1.053	1.150	1.05	1.049
*R_1_*	0.0864	0.1181	0.0411	0.0412
*wR_2_*	0.1628	0.2484	0.0917	0.0949
Largest diff. peak/hole [e·Å^−3^]	0.51/−0.66	1.12/−1.09	0.26/−0.34	0.64/−0.31
CCDC	2214190	2214208	2214232	2214239

**Table 2:** Crystal data and details of data collection for the ***b*** compounds.

	b2	b3	b4
Empirical formula	C_18_H_18.5_FeN_2_O_8.5_	C_18_H_14_N_2_NiO_6_	C_11_H_13_ClN_2_O_3_Pt
*Fw*	454.70	413.02	451.77
Space group	P4_1_2_1_2	*I2/a*	*Pna2_1_*
*a* [Å]	18.8731 (6)	7.4185 (17)	7.3424 (4)
*b* [Å]	18.8731 (6)	9.7198 (18)	8.5259 (5)
*c* [Å]	13.8168 (9)	22.442 (3)	19.5669 (12)
α [°]	90	90	90
β [°]	90	98.99 (2)	90
γ [°]	90	90	90
*V* [Å^3^]	4921.5 (4)	1598.3 (5)	1224.90 (12)
*Z*	8	4	4
*ρ*_calc_ [g cm^−3^]	1.227	1.716	2.45
Crystal size [mm^3^]	0.30 × 0.25 × 0.20	0.25 × 0.05 × 0.03	0.25 × 0.15 × 0.10
T [K]	293 (2)	293 (2)	293 (2)
*μ* [mm^−1^]	0.654	1.256	11.674
2Θ range	4.244 to 50.054	4.576 to 50.036	5.212 to 52.744
Reflections collected	24,895	2113	3345
Independent reflections	4318 [*R*_int_ = 0.0755]	2113 [*R*_int_ = 0.09]	1739 [*R*_int_ = 0.0287]
Data/restraints/parameters	4318/0/272	2113/6/125	1739/20/165
GOF	0.993	0.866	1.115
*R_1_*	0.0671	0.0658	0.0328
*wR_2_*	0.1611	0.0965	0.0791
Largest diff. peak/hole [e·Å^−3^]	0.47/−0.29	1.23/−0.41	1.26/−1.42
*CCDC*	2214267	2214276	2214287

**Table 3:** Crystal data and details of data collection for the ***c*** compounds.

	c2	c3
Empirical formula	C_16_H_14_Cl_4_N_2_O_2_	C_8_H_7_Br_2_NO
*Fw*	408.09	292.97
Space group	*I2/a*	*I2/a*
*a* [Å]	10.2571 (8)	10.3574 (6)
*b* [Å]	10.8832 (8)	11.1742 (5)
*c* [Å]	15.6160 (11)	15.6082 (9)
α [°]	90	90
β [°]	95.820 (7)	94.662 (5)
γ [°]	90	90
*V* [Å^3^]	1734.2 (2)	1800.45 (17)
*Z*	4	8
*ρ*_calc_ [g cm^−3^]	1.563	2.162
Crystal size [mm^3^]	0.10 × 0.04 × 0.02	0.60 × 0.05 × 0.03
T [K]	293 (2)	180.05 (10)
*μ* [mm^−1^]	0.694	8.953
2Θ range	4.57 to 57.85	4.488 to 58.244
Reflections collected	3778	4160
Independent reflections	1976 [*R*_int_ = 0.0286]	2098 [*R*_int_ = 0.0257]
Data/restraints/parameters	1976/0/113	2098/0/116
GOF	1.062	1.058
*R_1_*	0.0527	0.0324
*wR_2_*	0.1164	0.0644
Largest diff. peak/hole [e·Å^−3^]	0.34/−0.34	0.50/−0.62
*CCDC*	2214011	2214292

**Table 4:** Calculated data for target molecules (with N in the *β* position).

Molecule	Solvent	μ (D)	Mulliken Partial Charges			NPA from NBO			Δ_E_ * (eV)
			-N-	-Si-	-O-	-N-	-Si-	-O-	
**AEAMDS**	**Vacuum**	2.4415	−0.397	+1.268	−0.811	−0.682	+1.882	−1.253	6.521
	**Water**	4.1090	−0.421	+1.252	−0.817	−0.694	+1.878	−1.251	6.651
	**MeOH**	2.8238	−0.420	+1.257	−0.821	−0.693	+1.878	−1.251	6.619
**Silanol**	**Vacuum**	1.1179	−0.401	+1.124	−0.589	−0.681	+1.819	−1.058	6.593
	**Water**	1.3031	−0.425	+1.122	−0.625	−0.691	+1.820	−1.078	6.802
	**MeOH**	1.3005	−0.424	+1.122	−0.624	−0.691	+1.820	−1.077	6.794
**H-Silanol**	**Vacuum**	4.4604	−0.331	+1.103	−0.586	−0.531	+1.809	−1.057	6.154
	**Water**	8.8644	−0.326	+1.129	−0.610	−0.525	+1.814	−1.068	6.967
	**MeOH**	8.7698	−0.326	+1.129	−0.609	−0.525	+1.814	−1.067	6.936
**SB-Silanol**	**Vacuum**	2.5966	−0.404	+1.126	−0.588	−0.683	+1.820	−1.058	3.837
	**Water**	3.7811	−0.425	+1.123	−0.625	−0.691	+1.820	−1.078	3.967
	**MeOH**	3.7431	−0.424	+1.123	−0.624	−0.691	+1.820	−1.077	3.961
**SB-H-Silanol**	**Vacuum**	13.7299	−0.334	+1.104	−0.586	−0.529	+1.808	−1.057	4.360
	**Water**	15.7151	−0.329	+1.119	−0.615	−0.523	+1.813	−1.071	4.483
	**MeOH**	15.7617	−0.329	+1.118	−0.614	−0.524	+1.812	−1.071	4.482

* Δ_E_ (eV) = (E_LUMO_ − E_HOMO_)·27.21.

**Table 5:** Calculated data for APTMDS (with CH_2_ in the *β* position) and ClMTMDS (with Cl in the *β* position).

Molecule	Solvent	μ (D)	Mulliken Partial Charges			NPA From NBO			Δ_E_ * (eV)
			-CH_2_-/Cl-	-Si-	-O-	CH_2_-/Cl-	-Si-	-O-	
**APTMDS**	**Vacuum**	1.8565	−0.222	+1.265	−0.809	−0.378	+1.893	−1.245	7.171
	**Water**	2.426	−0.223	+1.270	−0.822	−0.380	+1.892	−1.247	7.231
	**MeOH**	2.4039	−0.224	+1.270	−0.822	−0.380	+1.892	−1.247	7.229
**ClMTMDS**	**Vacuum**	1.6688	−0.140	+1.288	−0.799	−0.091	+1.877	−1.244	7.465
	**Water**	4.2541	−0.183	+1.313	−0.806	−0.119	+1.876	−1.246	7.496
	**MeOH**	3.9327	−0.182	+1.313	−0.805	−0.118	+1.877	−1.247	7.490

* Δ_E_ (eV) = (E_LUMO_ − E_HOMO_)·27.21.

## Data Availability

The data presented in this work are available in the article and Supplementary Materials.

## Supplementary Materials

The following supporting information can be downloaded at: https://www.mdpi.com/article/10.3390/molecules27238563/s1, Chapter S1: FTIR analysis; Chapter S2: NMR analysis; Chapter S3: HMO-LUMO calculations; Chapter S4: X-ray crystallography; Chapter S5: Computational calculations.

## Sample Availability

Samples of the compounds are available from the authors.
