# Supplementary material for: Some Theoretical and Experimental Evidence for Particularities of the Siloxane Bond
Source: Molecules. 2022 Dec 5;27(23):8563. doi: 10.3390/molecules27238563 (PMC9736122; doi:10.3390/molecules27238563)
Supplement: Supplementary file 1 [file molecules-27-08563-s001.zip › molecules-2016608-supplementary.pdf]

Some theoretical and experimental evidence for particularities of the siloxane bond

Alexandru-Constantin Stoica,[1] Madalin Damoc,[1] Corneliu Cojocaru,[1] Alina Nicolescu,[2] Sergiu Shova,[1] Mihaela Dascalu[1] and Maria Cazacu[1]*

[1]*Department of Inorganic Polymers,"Petru Poni" Institute of Macromolecular Chemistry, Aleea Grigore Ghica Voda 41A, 700487 Iasi, Romania*

[2]*NMR Laboratory, "Petru Poni" Institute of Macromolecular Chemistry, Aleea Grigore Ghica Voda 41A, 700487 Iasi, Romania*

*Correspondence: mcazacu@icmpp.ro

S1. FTIR analysis

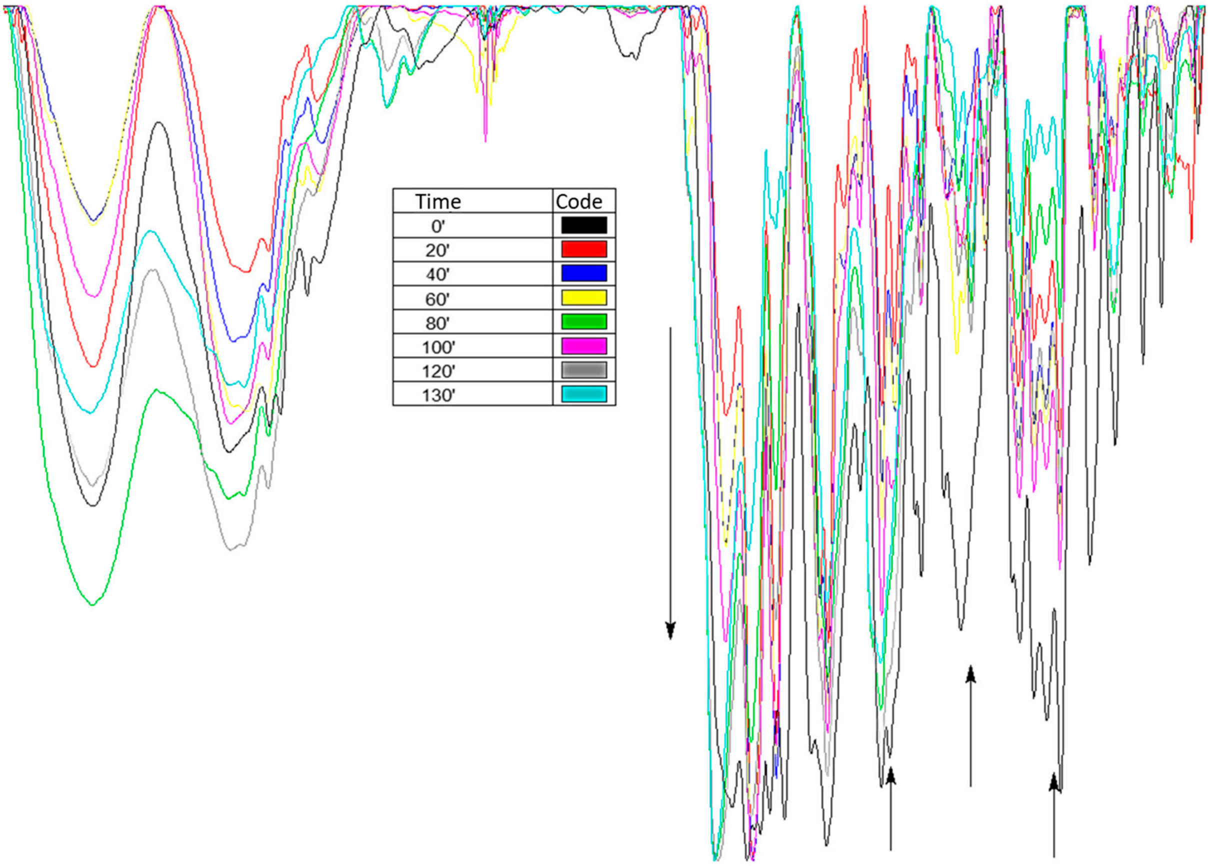

Figure S1a. Monitoring the reaction between AEAMDS and 3-formysalycilic acid (3FSA)

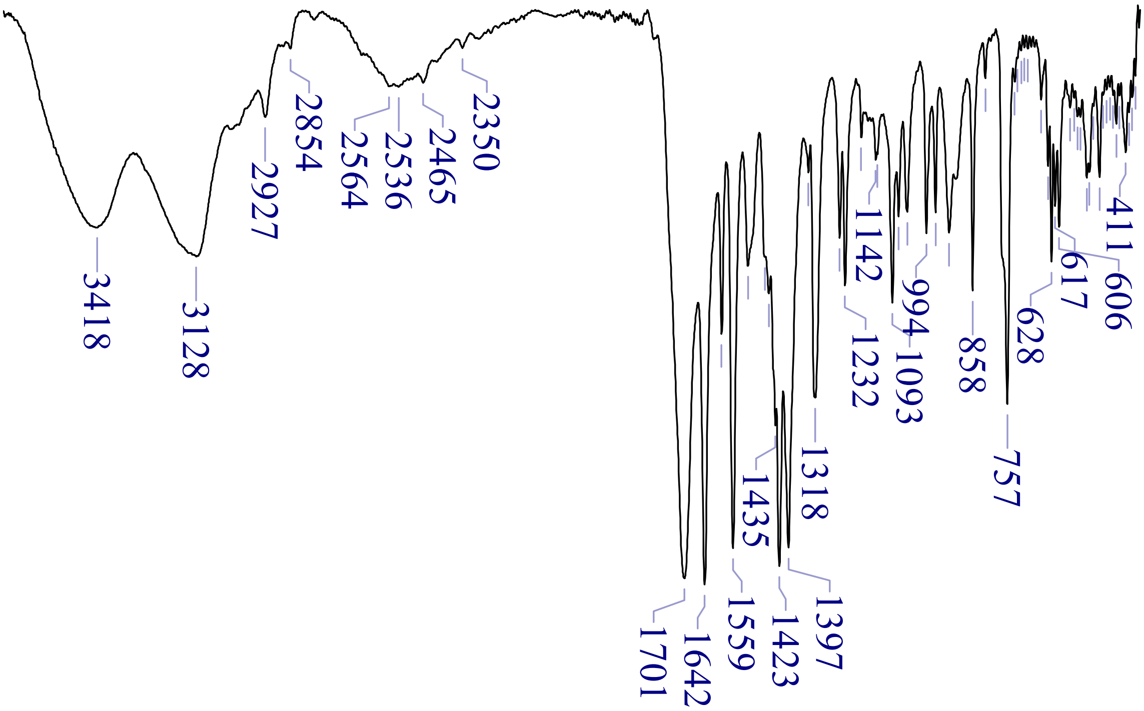

Figure S1b. FTIR spectra for a2

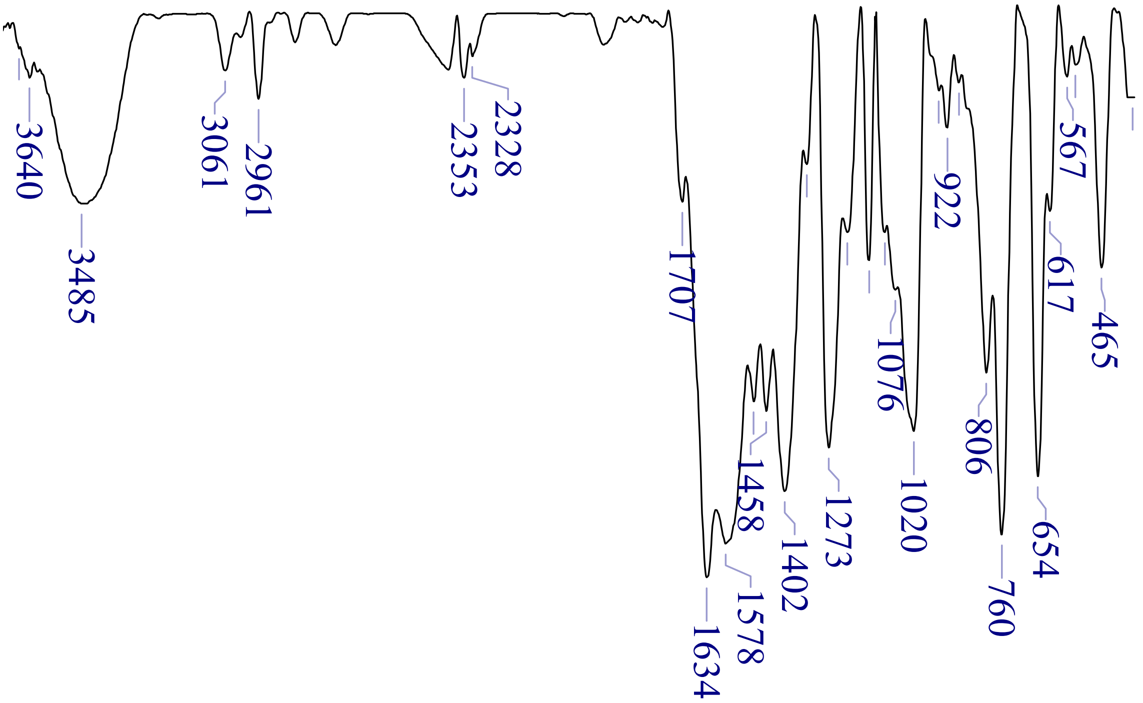

Figure S1c. FTIR spectra for a3

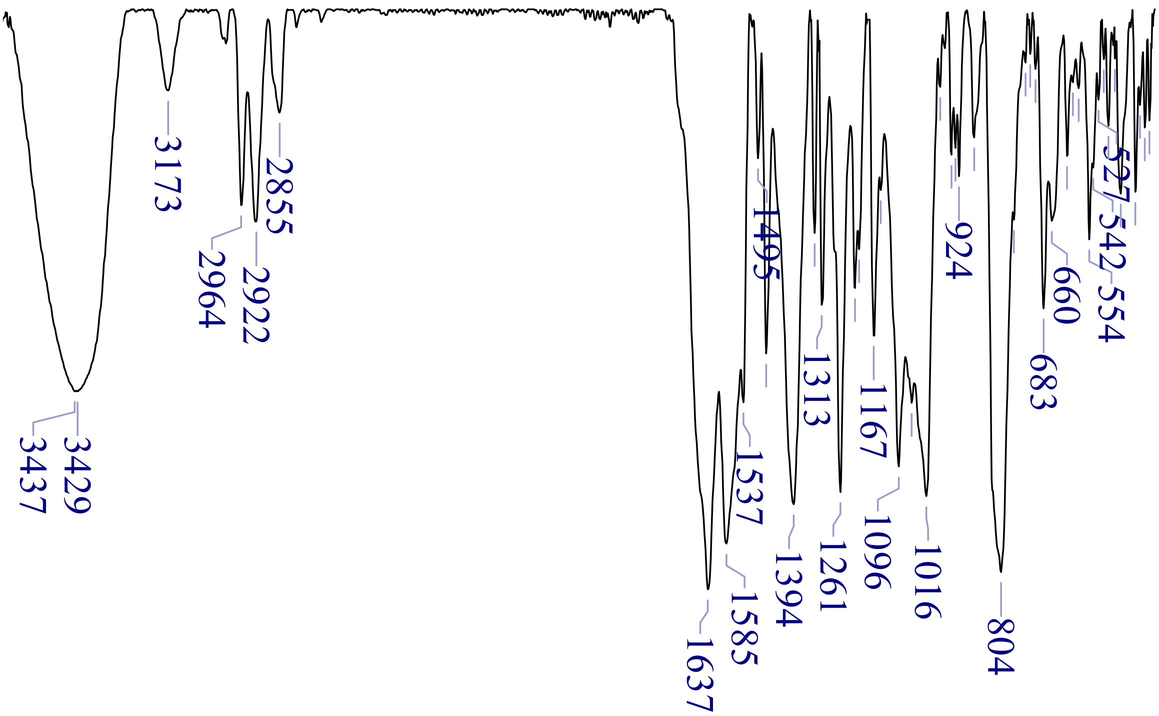

Figure S1d. FTIR spectra for a4

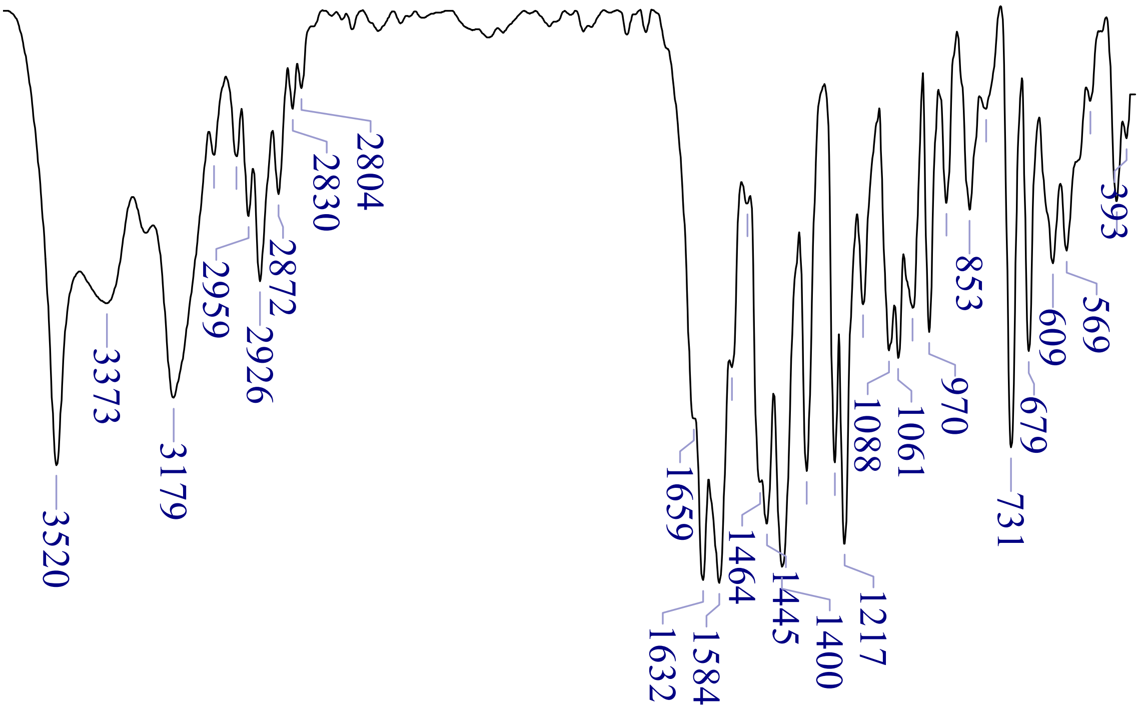

Figure S1e. FTIR spectra for a5

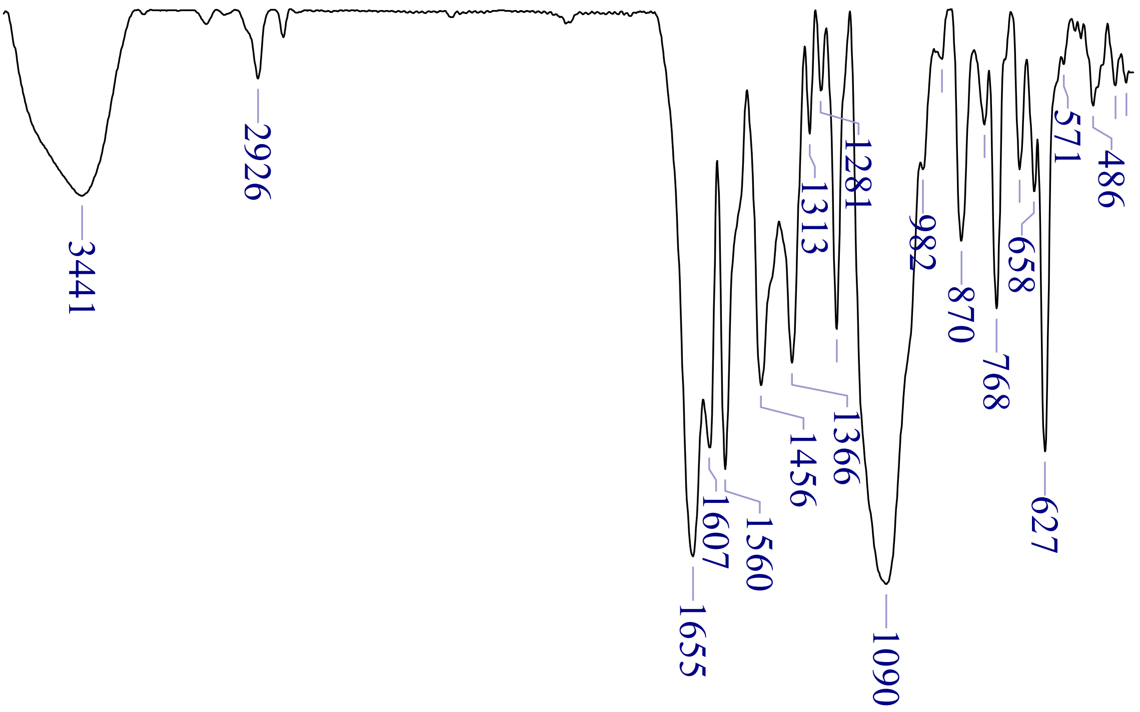

Figure S1f. FTIR spectra for b2

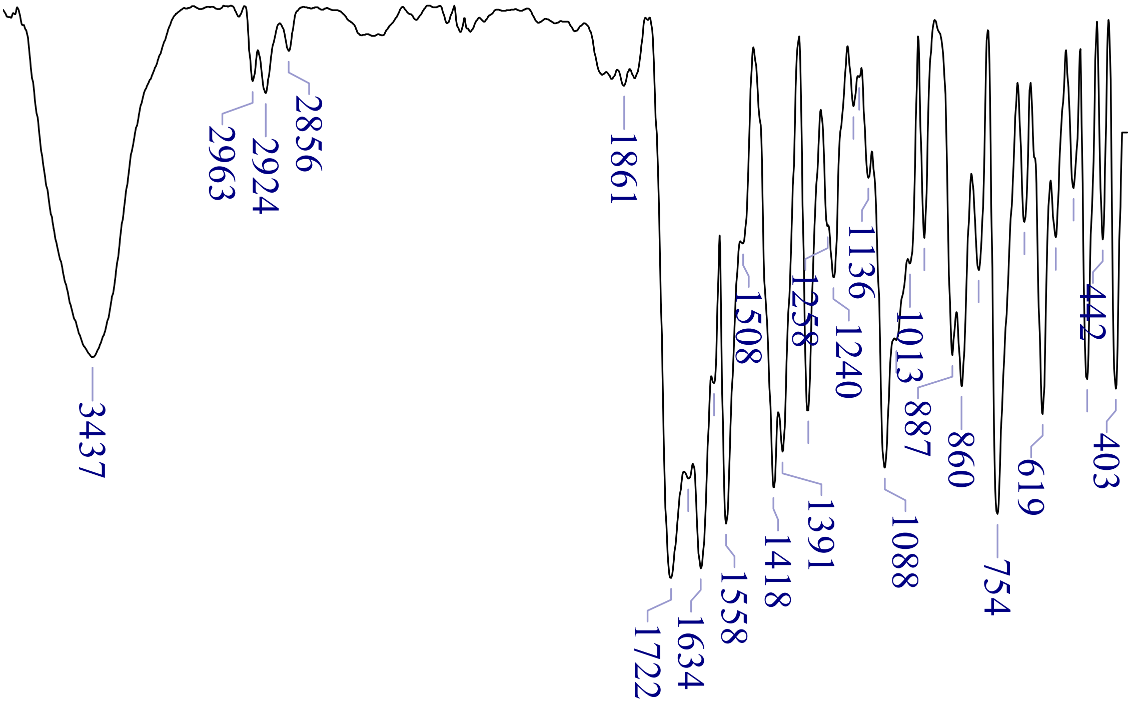

Figure S1g. FTIR spectra for b3

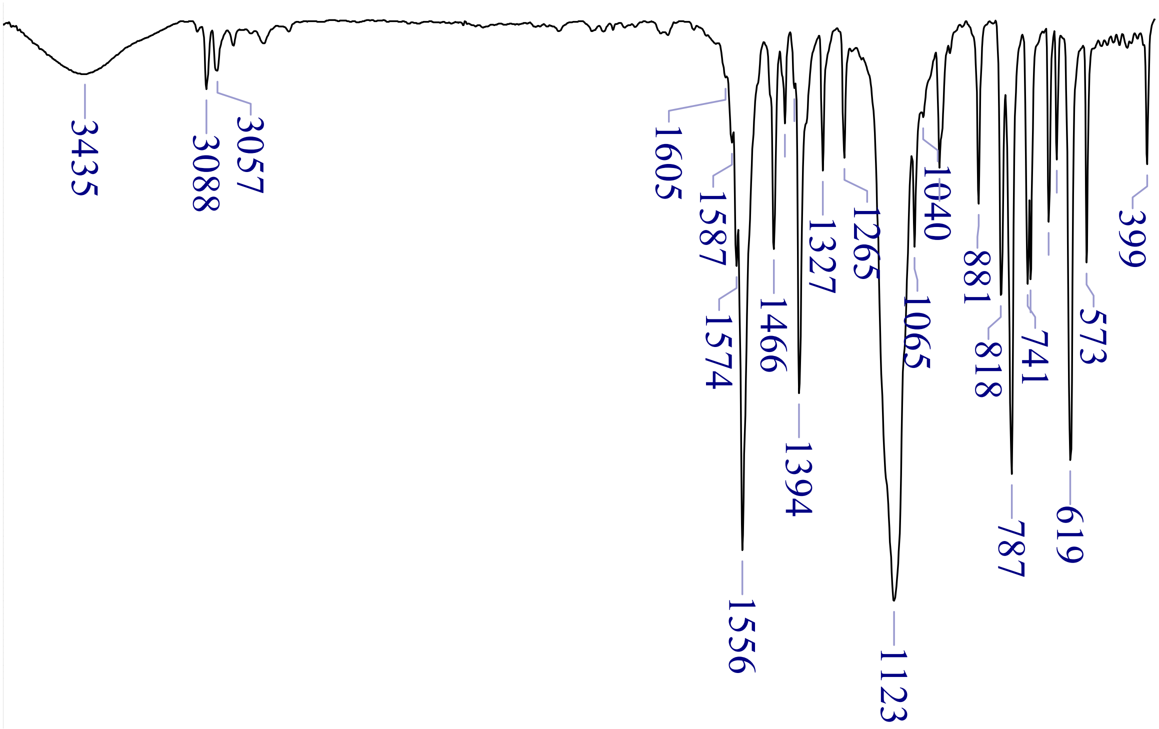

Figure S1h. FTIR spectra for b4

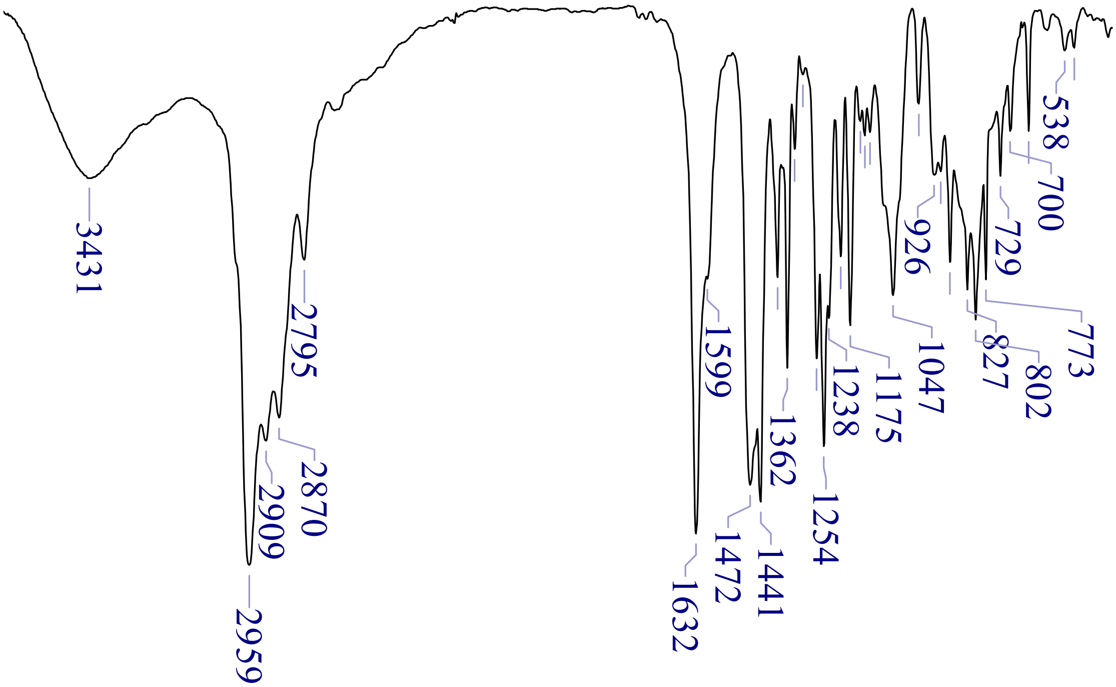

Figure S1i. FTIR spectra for c1

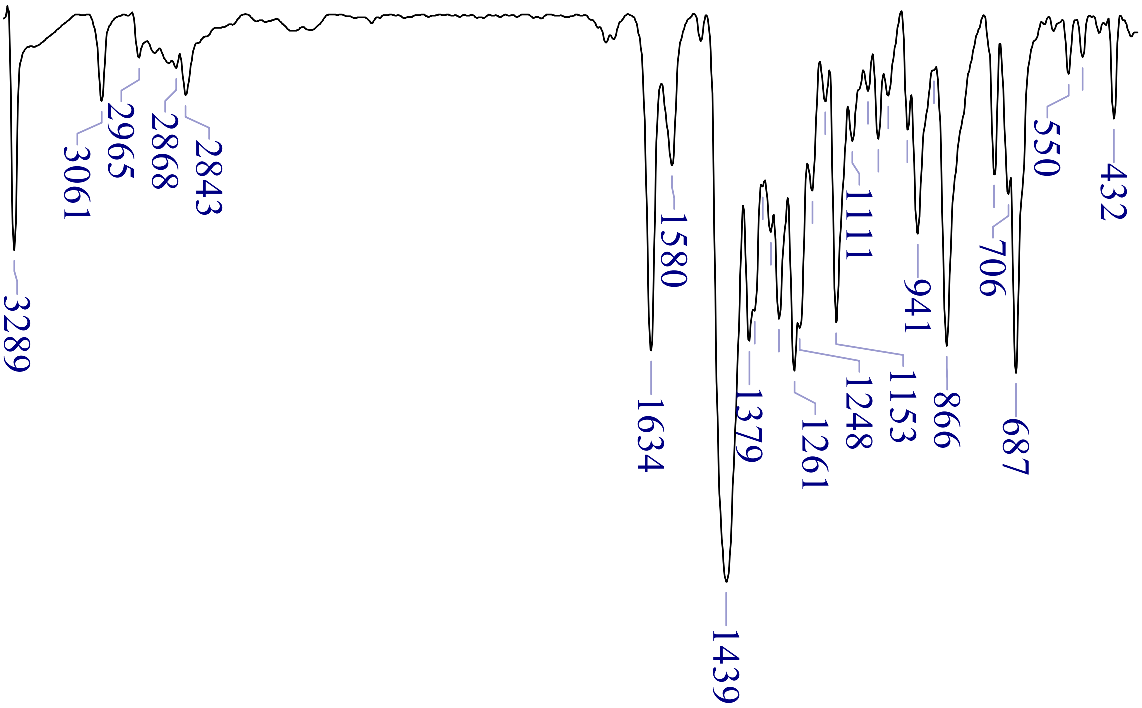

Figure S1j. FTIR spectra for c2

S2. NMR analysis

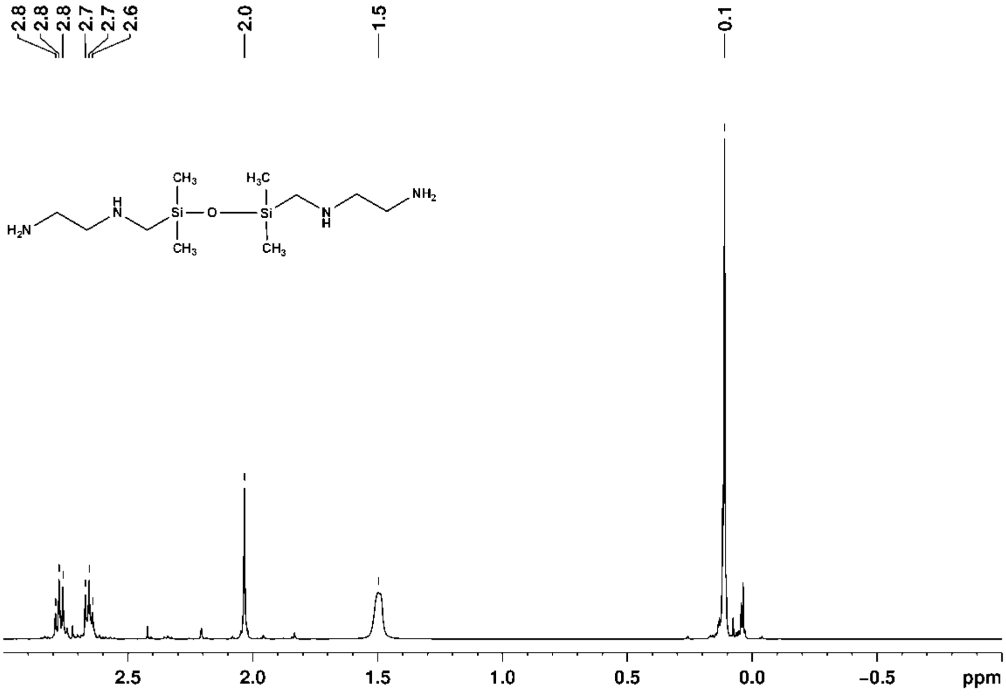

A)

B) 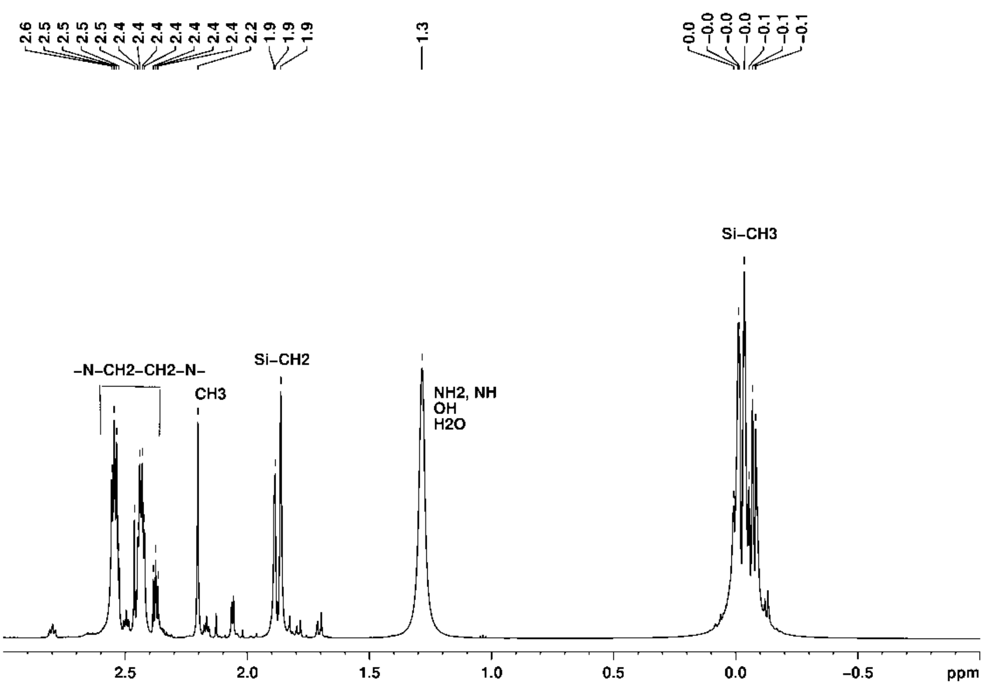

Figure S2a. A) ^{1}H NMR spectrum of initial AEAMDS, recorded in $CDCl_3$, showing five main signals, as expected for the existing chemical structure; and B) ^{1}H NMR spectrum of the same AEAMDS compound, neat, recorded after two year, showing a complex pattern that can be associated with the presence of several compounds.

NMR characteristics of the identified degradation compounds:

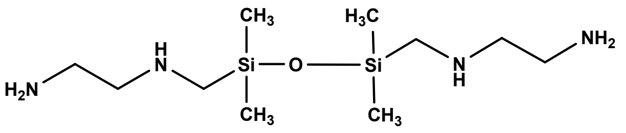

^{1}H-NMR (600.1 MHz, δ, neat) ppm: -0.06 (s, CH_3-Si), 1,30 (NH and NH_2, overlapped with H_2O), 1,85 (s, CH_2-Si), 2.42 (t, J = 6 Hz, CH_2-NH), 2.54 (t, J = 6 Hz, CH_2-NH_2).

^{13}C-NMR (150.9 MHz, δ, neat) ppm: 1.5 (CH_3-Si), 43.0 (CH_2-Si), 43.4 (CH_2-NH_2), 59.3 (CH_2-NH).

^{15}N-NMR (60.8 MHz, δ, neat) ppm: 17.4 (NH_2), 19.6 (NH).

^{29}Si-NMR (79.5 MHz, δ, neat) ppm: 4.63.

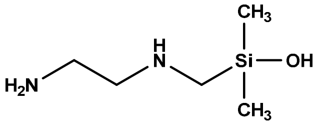

^{1}H-NMR (600.1 MHz, δ, neat) ppm: -0.00 (s, CH_3-Si), 1,30 (NH and NH_2, overlapped with H_2O), 1,88 (s, CH_2-Si), 2.42 (t, J = 6 Hz, CH_2-NH), 2.54 (t, J = 6 Hz, CH_2-NH_2).

^{13}C-NMR (150.9 MHz, δ, neat) ppm: 1.4 (CH_3-Si), 42.8 (CH_2-Si), 43.4 (CH_2-NH_2), 59.3 (CH_2-NH).

^{15}N-NMR (60.8 MHz, δ, neat) ppm: 17.4 (NH_2), 19.6 (NH).

^{29}Si-NMR (79.5 MHz, δ, neat) ppm: 4.27.

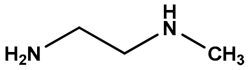

^{1}H-NMR (600.1 MHz, δ, neat) ppm: 1,30 (NH and NH2, overlapped with H_2O), 2.2 (s, CH_3-NH), 2.38 (t, J = 6 Hz, CH_2-NH), 2.54 (t, J = 6 Hz, CH_2-NH_2).

^{13}C-NMR (150.9 MHz, δ, neat) ppm: 38.4 (CH_3-NH), 43.6 (CH_2-NH_2), 57.2 (CH_2-NH).

^{15}N-NMR (60.8 MHz, δ, neat) ppm: 17.7 (NH_2), 19.6 (NH).

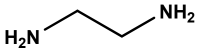

^{1}H-NMR (600.1 MHz, δ, neat) ppm: 2.45 (s, CH_2).

^{13}C-NMR (150.9 MHz, δ, neat) ppm: 47.3 (CH_2).

^{15}N-NMR (60.8 MHz, δ, neat) ppm: 16.9 (NH_2).

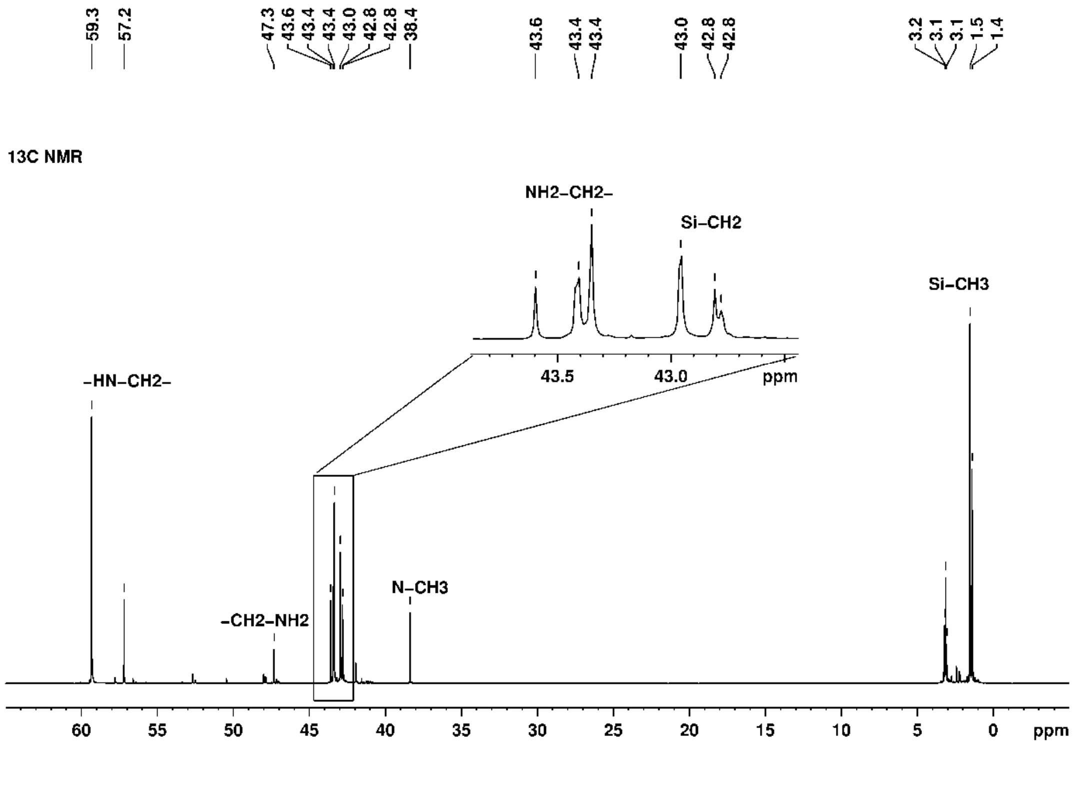

Figure S2b. ^{13}C-NMR spectrum corresponding to degraded AEAMDS, recorded neat, with DMSO-d_6 capillary.

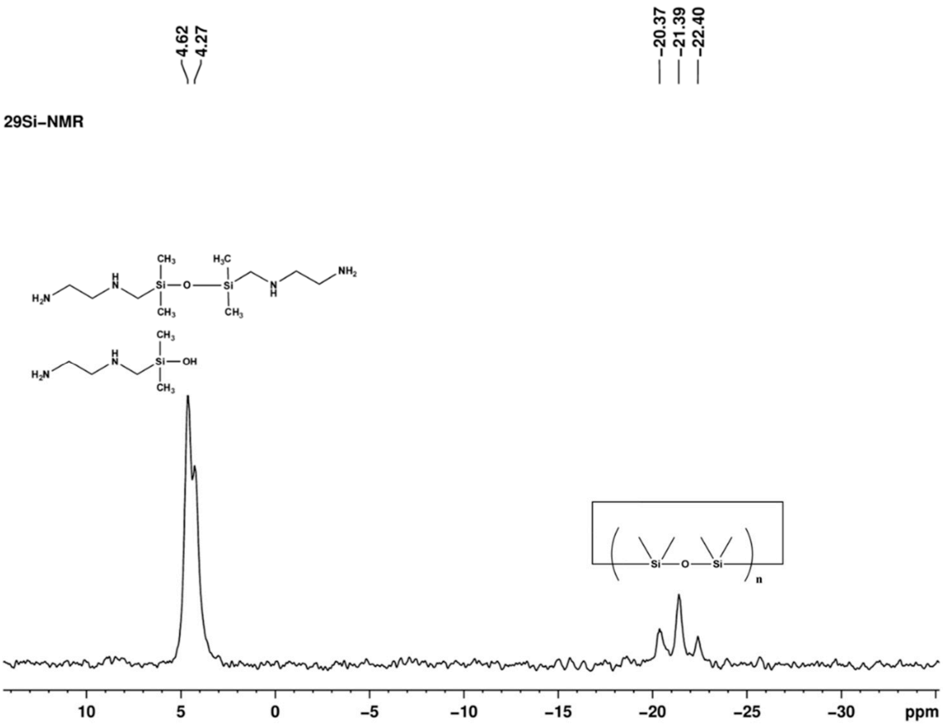

Figure S2c. ^{29}Si-NMR spectrum corresponding to degraded AEAMDS, recorded neat, with DMSO-d$_6$ capillary.

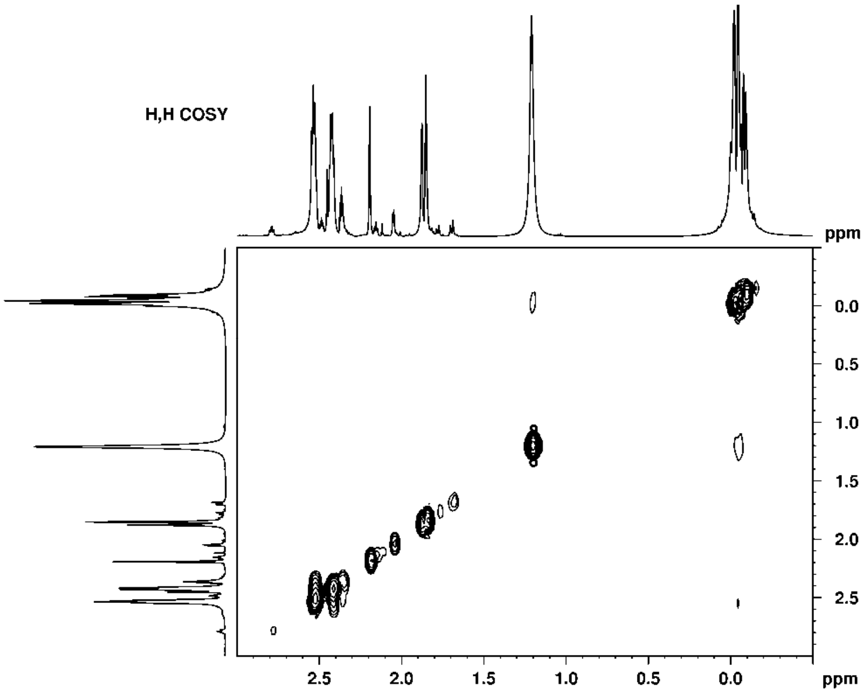

Figure S2d. H,H-COSY spectrum corresponding to degraded AEAMDS, recorded neat, with DMSO-d$_6$ capillary, showing the vicinal couplings between the methylene groups.

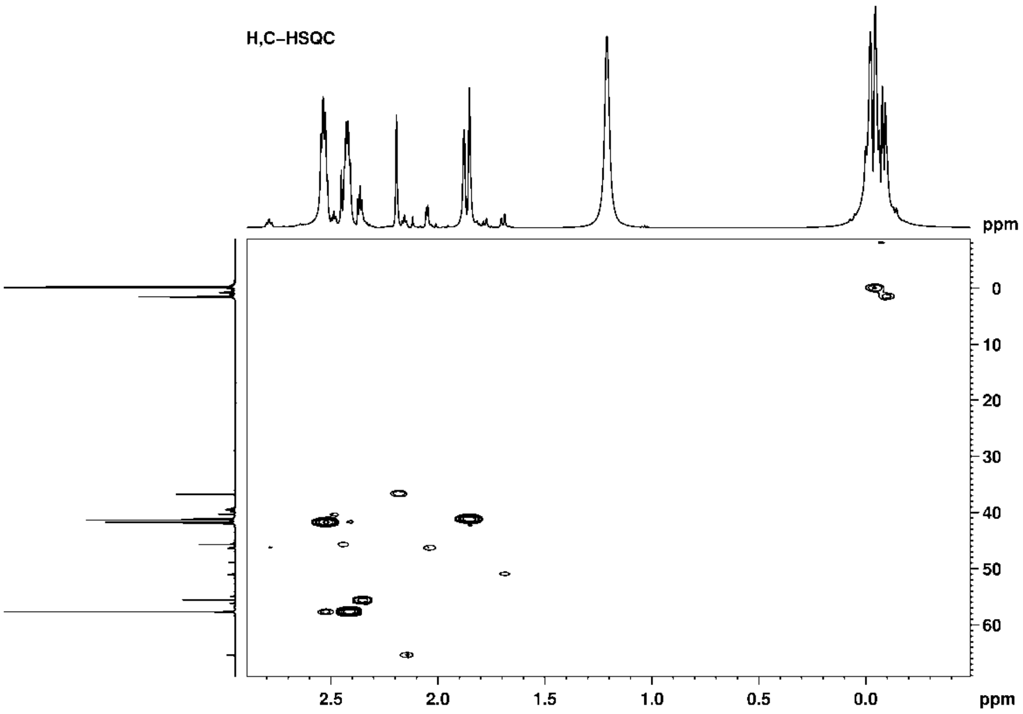

Figure S2f. H,C-HSQC spectrum corresponding to degraded AEAMDS, recorded neat, with DMSO-d_6 capillary, showing the direct correlations between protons and carbon atoms.

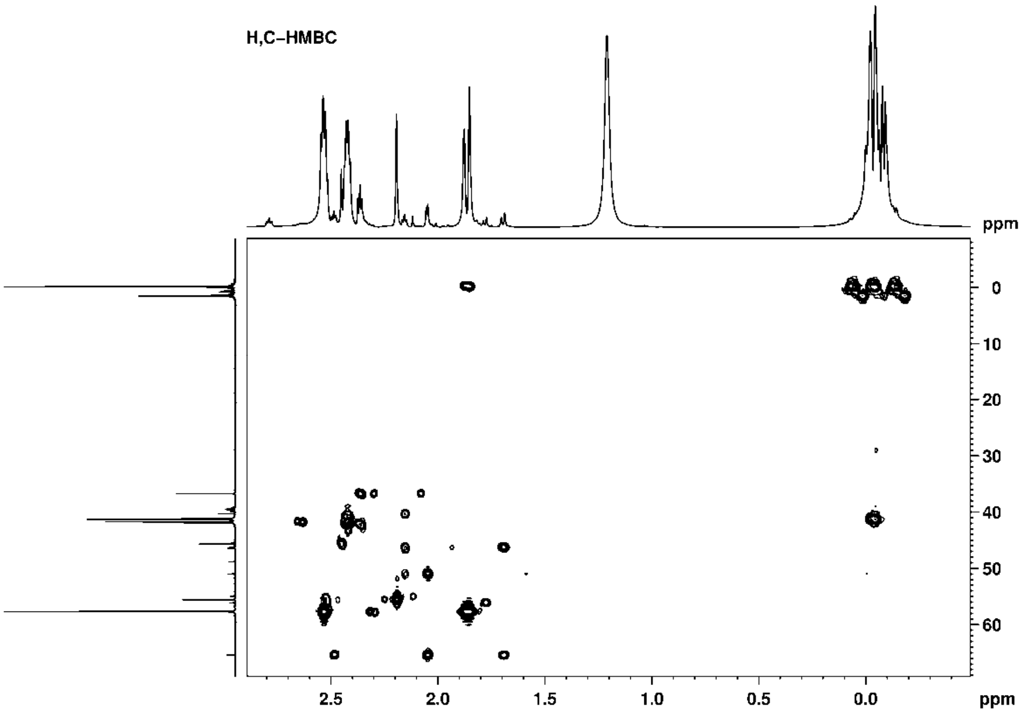

Figure S2g. H,C-HMBC spectrum corresponding to degraded AEAMDS, recorded neat, with DMSO-d_6 capillary, showing the long-range correlations between protons and carbon atoms (2 to 4 bonds apart).

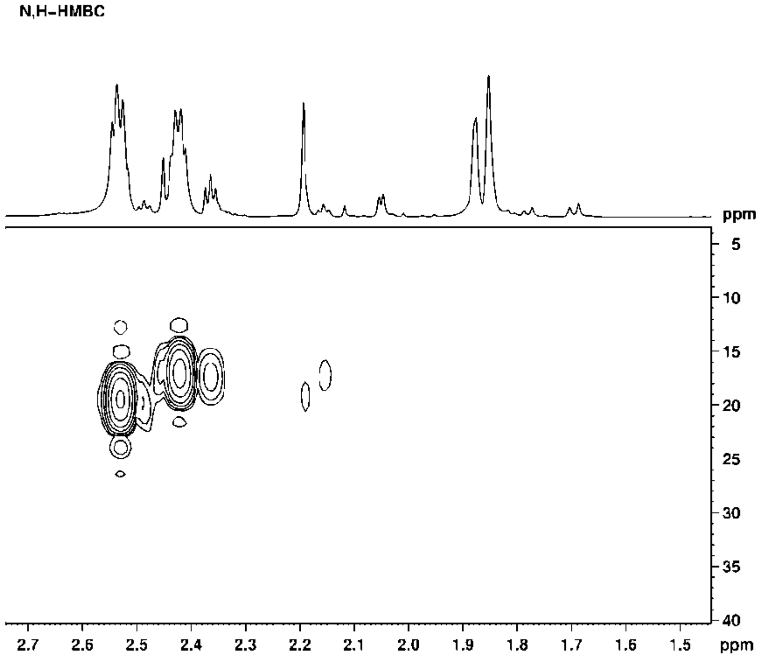

Figure S2h. H,N-HMBC spectrum corresponding to degraded AEAMDS, recorded neat, with DMSO-d_6 capillary, showing the long-range correlations between protons and nitrogen atoms (3 bonds apart). NH_2 resonates at 17.4 ppm, while NH at 19.6 ppm.

S3. HOMO-LUMO spatial distributions

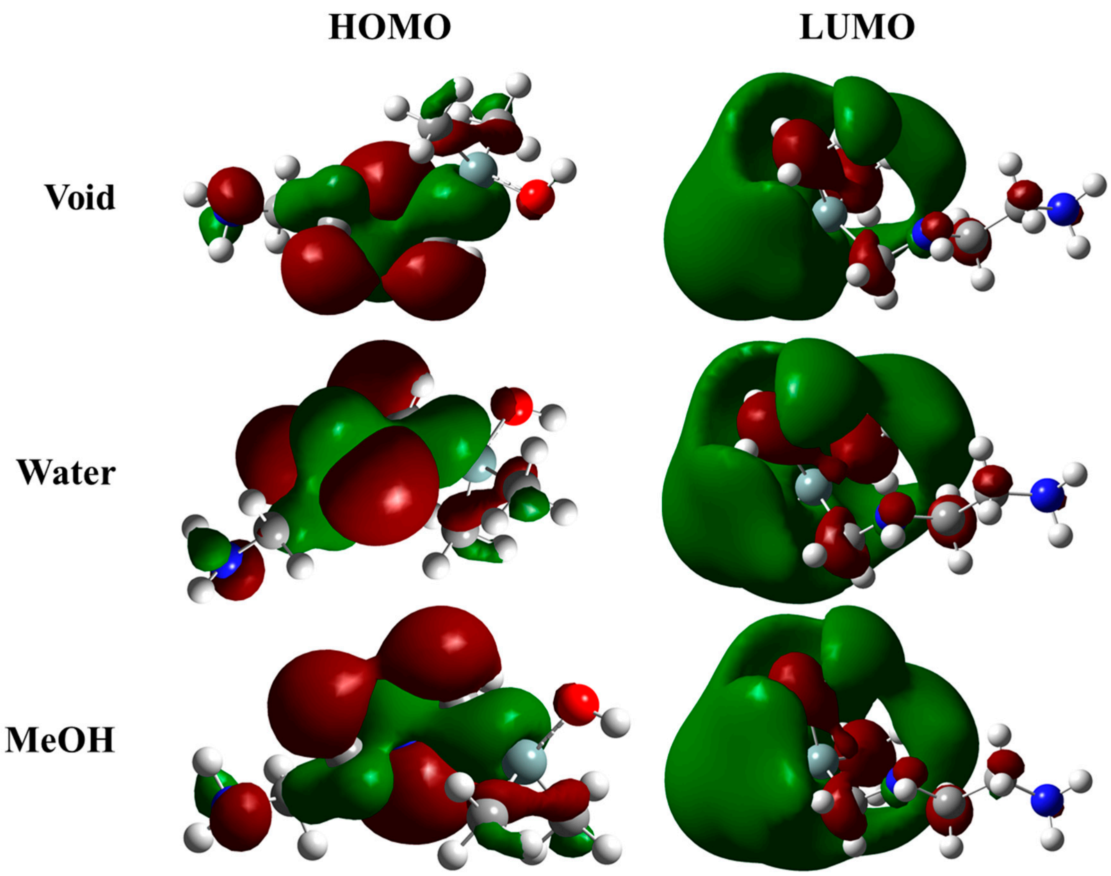

Figure S3. HOMO and LUMO images for silanol molecule

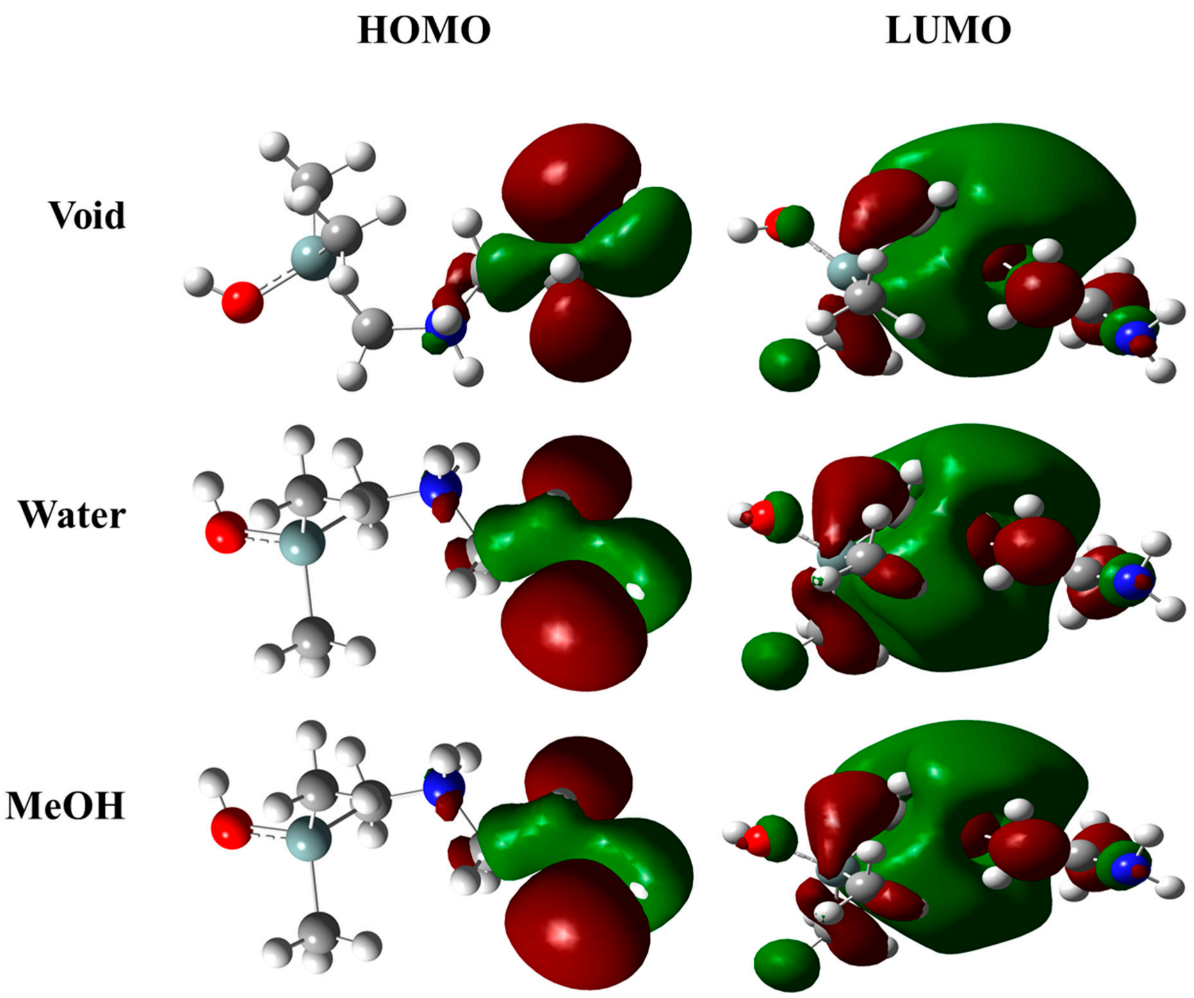

Figure S4. HOMO and LUMO images for H-Silanol molecule

S4. X-ray crystalography

Table S1. Bond distances (Å), angles (°) and hydrogen bonds for **a3**

Cu1-Cu11	0.000(4)	C1-C21	1.393(16)
Cu1-Cl1	2.295(4)	C1-C61	1.416(15)
Cu1-Cl11	2.295(4)	C1-C6	1.416(15)
Cu1-N21	2.054(10)	C2-C31	1.345(18)
Cu1-N2	2.054(10)	C2-C3	1.40(2)
O1-C1	1.311(13)	C3-C4	1.35(2)
N1-C7	1.296(16)	C4-C51	1.679(19)
N1-C8	1.479(16)	C4-C5	1.348(19)
N2-C9	1.500(15)	C5-C6	1.394(17)
N2-C10	1.384(14)	C6-C7	1.440(18)
N2-C101	1.384(14)	C8-C9	1.529(19)
C1-C2	1.393(16)	C8-C91	1.529(19)

Cu1^{1}-Cu1-Cl1	0(10)	C3^{1}-C2-C1	124.6(13)

Cu1^1-Cu1-N2	0(10)	C3^1-C2-C3	21(4)
Cl1-Cu1-Cl1^1	20.2(3)	C2^1-C3-C2	12(3)
N2-Cu1-Cl1	92.0(3)	C2^1-C3-C4	119.2(13)
N21-Cu1-Cl1^1	92.0(3)	C4-C3-C2	120.6(13)
N2-Cu1-Cl1^1	96.5(3)	C3-C4-C3^1	19(3)
N21-Cu1-Cl1	96.5(3)	C3-C4-C5^1	106.3(17)
N21-Cu1-N2	25.4(6)	C3-C4-C5	119.2(12)
Cu1^1-Cl1-Cu1	0.00(6)	C3^1-C4-C5^1	92.8(15)
C7-N1-C8	118.5(12)	C5-C4-C3^1	115.4(13)
C9-N2-Cu11	102.7(7)	C5-C4-C5^1	36.2(12)
C10^1-N2-Cu1^1	121.1(9)	C4-C5-C6	122.4(13)
C10-N2-Cu1	121.1(9)	C1-C6-C5^1	105.9(10)
C10-N2-Cu1^1	121.1(9)	C1-C6-C7	124.1(11)
C10^1-N2-Cu1	121.1(9)	C5-C6-C1	118.9(12)
C10-N2-C9	112.7(10)	C5-C6-C5^1	37.6(12)
C10^1-N2-C9	112.7(10)	C5-C6-C7	117.1(12)
C10^1-N2-C10	0.0(11)	C7-C6-C5^1	117.8(11)
O1^1-C1-O1	14.9(13)	N1-C7-N1^1	31.0(9)
O1^1-C1-C2^1	119.3(11)	N1^1-C7-C6^1	96.2(10)
O11-C1-C2	121.1(10)	N1-C7-C6	123.4(12)
O1-C1-C21	121.1(10)	N1-C7-C6^1	116.7(12)
O1-C1-C2	119.3(11)	C6-C7-N1^1	112.9(12)
O1-C1-C6^1	119.8(9)	C6-C7-C6^1	23.5(9)
O1^1-C1-C6^1	123.9(10)	N1-C8-N1^1	26.0(8)
O1-C1-C6	123.9(10)	N1-C8-C9^1	106.4(13)
O1^1-C1-C6	119.8(9)	N1-C8-C9	106.4(13)
C2^1-C1-C2	12(3)	C9-C8-N1^1	91.4(11)
C2-C1-C6	116.9(11)	C9^1-C8-N1^1	91.4(11)
C2^1-C1-C6	113.6(10)	C9^1-C8-C9	0.0(9)
C2-C1-C6^1	113.6(10)	N2-C9-N2^1	35.0(9)
C2^1-C1-C6^1	116.9(11)	N2-C9-C8	105.6(9)
C6^1-C1-C6	27.1(10)	N2^1-C9-C8	123.0(10)
C1-C2-C3	120.4(16)	N2^1-C10-N2	38.1(10)

^{1} + *x*, ½ – *y*, + *z*

D-H···A	d(D-H)/Å	d(H···A)/Å	d(D···A)/Å	D-H···A/°	Symmetry code
N2-H···Cl1^1	0.96	2.1	2.802(12)	128.9	1 – *x*, – *y*, 1 – *z*
N2-H···Cl1^2	0.96	2.71	3.509(12)	141.6	1 – *x*, – ½ + *y*, 1 – *z*
N2-H···O1^2	0.96	2.47	3.233(18)	136.6	1 – *x*, – ½ + *y*, 1 – *z*
C10-H···O1^3	0.95	2.34	3.227(16)	154.2	1 – *x*, 1 – *y*, 1 – *z*
C10-H···O1^4	0.95	2.65	3.550(16)	157.5	1 – *x*, ½ + *y*, 1 – *z*
C10-H···Cl1	0.98	2.77	3.284(14)	113.2	-

Single crystal X-ray difraction has releaved that compound **a3** has a molecular crystal structure. Its asymetric unit comprises one complex molecule [CuHL]. Cu^{2+} atom exhibited sligthly distorted N_2OCl square planes coordination provided by HL unit, which plays the

role of three-dentate ligand. The central atom Cu^{2+} is displaced from the mean plane of coordination atom at 0.266(6) Å.

Table S2. Bond distances (Å), angles (°) and hydrogen bonds for **a4**

Cu1-O1	1.932(7)	C1-C6	1.404(4)
Cu1-O2^{1}	2.424(7)	C2-C3	1.404(4)
Cu1-O2	1.966(7)	C3-C4	1.404(4)
Cu1-N1	1.962(8)	C4-C5	1.404(4)
Cu1-N2	2.024(9)	C4-C11	1.453(10)
O1-C1	1.330(11)	C5-C6	1.404(4)
O2-C12	1.286(12)	C6-C7	1.452(10)
O3-C12	1.247(13)	C8-C9	1.532(15)
N1-C7	1.277(12)	C12-C13	1.497(15)
N1-C8	1.472(9)	N3-C14	1.301(14)
N2-C9	1.472(9)	N3-C15	1.300(14)
N2-C10	1.470(13)	C14-C14^{2}	1.47(2)
C1-C2	1.404(4)	C15-C15^{2}	1.46(2)

O1-Cu1-O2	90.2(3)	O1-C1-C6	124.6(7)
O1-Cu1-O2^{1}	99.4(3)	C2-C1-C6	117.9(8)
O1-Cu1-N1	92.1(3)	C3-C2-C1	120.2(9)
O1-Cu1-N2	173.5(3)	C2-C3-C4	123.5(9)
O2-Cu1-O2^{1}	76.2(3)	C3-C4-C11	122.1(9)
O2-Cu1-N2	93.6(3)	C5-C4-C3	114.4(9)
N1-Cu1-O2	177.3(3)	C5-C4-C11	123.5(10)
N1-Cu1-O2^{1}	104.8(3)	C6-C5-C4	123.8(9)
N1-Cu1-N2	84.0(3)	C1-C6-C7	123.1(7)
N2-Cu1-O2^{1}	86.7(3)	C5-C6-C1	119.9(8)
C1-O1-Cu1	127.4(5)	C5-C6-C7	117.0(7)
Cu1-O2-Cu1^{1}	103.8(3)	N1-C7-C6	125.2(8)
C12-O2-Cu1^{1}	142.5(7)	N1-C8-C9	107.0(7)
C12-O2-Cu1	113.6(6)	N2-C9-C8	105.9(8)
C7-N1-Cu1	127.3(6)	O2-C12-C13	118.0(10)
C7-N1-C8	120.7(8)	O3-C12-O2	122.2(10)
C8-N1-Cu1	111.9(6)	O3-C12-C13	119.7(10)
C9-N2-Cu1	107.6(6)	C15-N3-C14	102.5(17)
C10-N2-Cu1	120.2(7)	N3-C14-C14^{2}	119.8(13)
C10-N2-C9	111.1(9)	N3-C15-C15^{2}	114.9(15)
O1-C1-C2	117.4(7)		

$^{1}1 - x, 2 - y, 1 - z$; $^{2}1 - x, + y, ½ - z$

**D-H···A**	**d(D-H)/Å**	**d(H···A)/Å**	**d(D-A)/Å**	**D-H···A/°**	**Symmetry code**
N2-H···O1*w*	1	1.95	2.900(12)	158.2	-
C8-H···O3^{1}	0.99	2.56	3.249(11)	126.7	$½ - x, ½ + y, + z$
O1*w*-H···O3^{2}	0.85	1.95	2.757(11)	157.3	$+ x, 1 + y, + z$

O1*w*-H···O1[3]	0.85	2.09	2.822(10)	143.6	$1-x, 2-y, 1-z$

The asymmetric unit of the crystals **a4** and **a5** is build up from similar dinuclear neutral complexes [$Cu_2(HL_1)_2Ac_2$] and [$Cu_2(HL_2)_2Ac_2$], respectively and interstitial water molecules in 1:1 ratio. In the centro-symetric dinuclear units, Cu(II) atoms are bridged by two μ-oxo oxygen atoms of two acetate anions at 3.465(2) Å, 3.4236(7) Å separations for **a4** and **a5**, respectively. The coordination of each copper atom is completed by N_2O coordination set provided by HL_1^- and HL_2^- (for a4 and a5, respectively) three-deentate anion ligands. It is to be mentioned, that the molecular structure is stabilized by hydrogen bonding involving co-crystalized water molecule (see **Figure 1 a4, a5**).

Table S3. Bond distances (Å), angles (°) and hydrogen bonds for **a5**

Cu1-O1	1.929(2)	N2-C9	1.483(4)
Cu1-O3	1.9676(17)	N2-C10	1.468(4)
Cu1-O3[1]	2.4001(19)	C1-C2	1.424(4)
Cu1-N1	1.950(2)	C1-C6	1.419(4)
Cu1-N2	2.046(2)	C2-C3	1.369(5)
O1-C1	1.303(3)	C3-C4	1.385(5)
O2-C2	1.371(4)	C4-C5	1.357(5)
O2-C11	1.423(4)	C5-C6	1.410(4)
O3-C12	1.276(3)	C6-C7	1.430(4)
O4-C12	1.230(3)	C8-C9	1.499(4)
N1-C7	1.286(4)	C12-C13	1.516(4)
N1-C8	1.471(4)		

O1-Cu1-O3[1]	94.88(8)	C10-N2-C9	111.6(2)
O1-Cu1-O3	90.95(8)	O1-C1-C2	118.6(3)
O1-Cu1-N1	92.25(9)	O1-C1-C6	125.1(3)
O1-Cu1-N2	173.39(9)	C6-C1-C2	116.3(3)
O3-Cu1-O3[1]	77.22(8)	O2-C2-C1	113.0(3)
O3-Cu1-N2	93.59(9)	C3-C2-O2	125.2(3)
N1-Cu1-O3[1]	105.86(9)	C3-C2-C1	121.8(3)
N1-Cu1-O3	175.34(9)	C2-C3-C4	120.7(4)
N1-Cu1-N2	82.95(10)	C5-C4-C3	119.8(3)
N2-Cu1-O3[1]	90.82(9)	C4-C5-C6	121.3(4)
C1-O1-Cu1	127.55(19)	C1-C6-C7	122.3(3)
C2-O2-C11	118.5(3)	C5-C6-C1	120.1(3)
Cu1-O3-Cu1[1]	102.78(8)	C5-C6-C7	117.5(3)
C12-O3-Cu1	115.29(18)	N1-C7-C6	125.9(3)
C12-O3-Cu1[1]	141.89(18)	N1-C8-C9	107.1(3)
C7-N1-Cu1	126.8(2)	N2-C9-C8	106.9(3)
C7-N1-C8	119.3(3)	O3-C12-C13	115.9(3)
C8-N1-Cu1	113.91(19)	O4-C12-O3	123.8(3)
C9-N2-Cu1	106.08(18)	O4-C12-C13	120.2(3)

C10-N2-Cu1	120.9(2)		

[1] $1-x, 1-y, 1-z$

D-H···A	d(D-H)/Å	d(H···A)/Å	d(D-A)/Å	D-H···A/°	Symmetry code
N2-H···O1w[1]	0.98	1.93	2.902(4)	171.4	$1-x, 1-y, 1-z$
C7-H···O4[2]	0.93	2.5	3.390(4)	159.5	$½+x, 3/2-y, 1-z$

Table S4. Bond distances (Å), angles (°) and hydrogen bonds for **a6**

Cu1-O1	1.934(2)	C1-C6	1.426(4)
Cu1-O2	1.916(2)	C2-C3	1.363(5)
Cu1-O3	2.395(3)	C3-C4	1.394(5)
Cu1-N1	1.946(3)	C4-C5	1.362(5)
Cu1-N2	1.956(3)	C5-C6	1.399(5)
Cl1-C4	1.750(4)	C6-C7	1.447(4)
Cl2-C13	1.745(4)	C8-C9	1.442(5)
O1-C1	1.307(4)	C10-C11	1.438(5)
O2-C16	1.298(4)	C11-C12	1.400(5)
O3-C17	1.383(5)	C11-C16	1.425(4)
N1-C7	1.268(4)	C12-C13	1.353(5)
N1-C8	1.467(4)	C13-C14	1.399(5)
N2-C9	1.461(4)	C14-C15	1.363(5)
N2-C10	1.272(4)	C15-C16	1.416(5)
C1-C2	1.415(4)		

O1-Cu1-O3	98.07(10)	C3-C4-Cl1	120.4(3)
O1-Cu1-N1	92.68(10)	C5-C4-Cl1	119.5(3)
O1-Cu1-N2	168.58(11)	C5-C4-C3	120.0(3)
O2-Cu1-O1	89.81(9)	C4-C5-C6	121.1(3)
O2-Cu1-O3	91.36(10)	C1-C6-C7	122.6(3)
O2-Cu1-N1	174.44(10)	C5-C6-C1	120.2(3)
O2-Cu1-N2	93.02(10)	C5-C6-C7	117.2(3)
N1-Cu1-O3	93.22(11)	N1-C7-C6	125.7(3)
N1-Cu1-N2	83.59(11)	C9-C8-N1	110.6(3)
N2-Cu1-O3	92.93(11)	C8-C9-N2	111.6(3)
C1-O1-Cu1	127.2(2)	N2-C10-C11	126.1(3)
C16-O2-Cu1	127.4(2)	C12-C11-C10	117.6(3)
C17-O3-Cu1	128.0(3)	C12-C11-C16	119.7(3)
C7-N1-Cu1	126.9(2)	C16-C11-C10	122.7(3)
C7-N1-C8	119.6(3)	C13-C12-C11	122.0(3)
C8-N1-Cu1	113.2(2)	C12-C13-Cl2	120.9(3)
C9-N2-Cu1	113.1(2)	C12-C13-C14	119.8(3)
C10-N2-Cu1	125.7(2)	C14-C13-Cl2	119.2(3)
C10-N2-C9	121.1(3)	C15-C14-C13	119.3(3)
O1-C1-C2	119.4(3)	C14-C15-C16	123.2(3)
O1-C1-C6	124.5(3)	O2-C16-C11	124.5(3)

C2-C1-C6	116.1(3)	O2-C16-C15	119.6(3)
C3-C2-C1	122.6(3)	C15-C16-C11	116.0(3)
C2-C3-C4	119.8(3)		

D-H···A	d(D-H)/Å	d(H···A)/Å	d(D-A)/Å	D-H···A/°	Symmetry code
O3-H···O1[1]	0.861(9)	1.928(12)	2.783(3)	172(3)	$1-x, 1-y, 1-z$
C8-H···Cl1[2]	0.97	2.95	3.842(5)	153.7	$+x, 1+y, +z$
C8-H···Cl2[3]	0.97	2.94	3.626(5)	128.7	$+x, +y, -1+z$
C9-H···O2[4]	0.97	2.63	3.400(5)	136.4	$2-x, 1-y, 1-z$

In molecular complex **a6** [CuL$_3$MeOH] the Cu(II) is five-coordinated, in distorted square-pyramidal coordination formed by tetradentate Schiff base ligand at the base and MeOH in apical coordination. Cu(II) atom is displaced from the mean plane N$_2$O$_2$ atoms at 0.1401(13) Å to towards oxygen atom of coordinated MeOH.

Table S5. Bond distances (Å), angles (°) and hydrogen bonds for **b2**

Fe1-O1	1.946(5)	C2-C3	1.368(10)
Fe1-O1*w*	2.031(5)	C2-C7	1.414(10)
Fe1-O2*w*	2.068(5)	C3-C4	1.429(10)
Fe1-O3	1.981(5)	C4-C5	1.385(12)
Fe1-O4	1.960(5)	C4-C8	1.384(11)
Fe1-O6	1.963(5)	C5-C6	1.369(13)
O1-C1	1.286(8)	C6-C7	1.380(11)
O2-C1	1.248(8)	C9-C10	1.509(13)
O3-C3	1.324(8)	C11-C12	1.487(15)
O4-C18	1.276(10)	C12-C13	1.386(14)
O5-C18	1.235(10)	C12-C17	1.422(11)
O6-C17	1.303(10)	C13-C14	1.337(15)
N1-C8	1.323(11)	C14-C15	1.359(14)
N1-C9	1.461(11)	C15-C16	1.395(12)
N2-C10	1.450(12)	C16-C17	1.400(12)
N2-C11	1.267(12)	C16-C18	1.485(12)
C1-C2	1.484(10)		

O1-Fe1-O1*w*	92.5(2)	O3-C3-C4	115.9(7)
O1-Fe1-O2*w*	87.0(2)	C2-C3-C4	122.4(7)
O1-Fe1-O3	86.4(2)	C5-C4-C3	117.3(8)
O1-Fe1-O4	98.9(2)	C8-C4-C3	124.5(8)
O1-Fe1-O6	170.6(2)	C8-C4-C5	118.2(8)
O1*w*-Fe1-O2*w*	176.7(2)	C6-C5-C4	122.2(8)
O3-Fe1-O1*w*	85.0(2)	C5-C6-C7	118.9(8)
O3-Fe1-O2*w*	91.7(2)	C6-C7-C2	122.4(8)
O4-Fe1-O1*w*	90.2(2)	N1-C8-C4	123.5(8)
O4-Fe1-O2*w*	93.1(2)	N1-C9-C10	109.2(8)

O4-Fe1-O3	173.0(2)	N2-C10-C9	110.1(8)
O4-Fe1-O6	88.6(2)	N2-C11-C12	125.0(8)
O6-Fe1-O1*w*	93.2(2)	C13-C12-C11	121.1(10)
O6-Fe1-O2*w*	87.0(2)	C13-C12-C17	119.6(11)
O6-Fe1-O3	86.6(2)	C17-C12-C11	119.1(8)
C1-O1-Fe1	130.4(5)	C14-C13-C12	121.6(11)
C3-O3-Fe1	125.8(4)	C13-C14-C15	119.4(10)
C18-O4-Fe1	131.1(5)	C14-C15-C16	123.0(10)
C17-O6-Fe1	126.9(5)	C15-C16-C17	117.8(9)
C8-N1-C9	126.0(8)	C15-C16-C18	118.2(9)
C11-N2-C10	123.7(8)	C17-C16-C18	124.0(8)
O1-C1-C2	119.1(6)	O6-C17-C12	118.0(8)
O2-C1-O1	119.3(6)	O6-C17-C16	123.5(7)
O2-C1-C2	121.5(7)	C16-C17-C12	118.5(9)
C3-C2-C1	125.0(6)	O4-C18-C16	120.1(8)
C3-C2-C7	116.8(7)	O5-C18-O4	121.5(8)
C7-C2-C1	117.8(6)	O5-C18-C16	118.4(8)
O3-C3-C2	121.7(6)		

D-H···A	d(D-H)/Å	d(H···A)/Å	d(D···A)/Å	D-H···A/°	Symmetry code
O1*w*-H···O5[1]	0.85	1.78	2.581(8)	154.7	$+x, +y, -z$
O1*w*-H···O3*w*	0.85	1.91	2.733(10)	162.8	–
O2*w*-H···O2[2]	0.85	1.78	2.626(7)	176.9	$-½+x, {}^{3}/_{2}-y, ¼+z$
O3-H···N1	0.88	1.81	2.583(8)	146.3	–
O3*w*-H···N1	0.86	2.43	3.177(12)	145.5	–
O6-H···N2	0.87	1.7	2.564(9)	172.3	–
C8-H···O1*w*[2]	0.93	2.57	3.347(10)	141.7	$-½+x, {}^{3}/_{2}-y, ¼+z$
C10-H···O1[3]	0.97	2.6	3.470(10)	148.9	${}^{3}/_{2}-x, -½+y, ¼-z$
C10-H···O1[2]	0.97	2.63	3.503(11)	149.8	$-½+x, {}^{3}/_{2}-y, ¼+z$
C11-H···O2[3]	0.93	2.42	3.138(11)	133.8	${}^{3}/_{2}-x, -½+y, ¼-z$

Single crystal X-ray analysis has revealed that compound **b2** has a molecular crystal structure. Its asymetric unit comprises one complex molecule [$FeH2L_4(2H_2O)$] and interstitial water molecules in 1:1 ratio. Fe^{2+} has a squared-pyramidal geometry, the base of the pyramid consists of four oxygen atoms, and two water molecules are coordinated in the apical position, $H_2L_4^{2-}$ unit has the role of tetra-dentate ligand. The asymmetric unit is stabilized by intra-molecular hydrogen bonds O-H···N.

Table S6. Bond distances (Å), angles (°) and hydrogen bonds for **b3**

Ni1-O1[1]	1.865(4)	C1-C2	1.410(8)
Ni1-O1	1.865(4)	C1-C6	1.419(9)
Ni1-N1	1.841(6)	C2-C3	1.393(7)
Ni1-N1[1]	1.841(6)	C2-C9	1.490(9)

O1-C1	1.321(9)	C3-C4	1.372(9)
O2-C9	1.349(8)	C4-C5	1.360(9)
O3-C9	1.207(8)	C5-C6	1.409(7)
N1-C7	1.312(9)	C6-C7	1.419(10)
N1-C8	1.469(8)	C8-C8^{1}	1.445(12)

O1-Ni1-O1^{1}	86.6(3)	C3-C2-C1	120.6(7)
N1-Ni1-O1	94.1(2)	C3-C2-C9	116.7(7)
N1^{1}-Ni1-O1^{1}	94.1(2)	C4-C3-C2	121.4(7)
N1^{1}-Ni1-O1	177.4(3)	C5-C4-C3	119.6(6)
N1-Ni1-O1^{1}	177.4(3)	C4-C5-C6	121.2(7)
N1^{1}-Ni1-N1	85.3(4)	C5-C6-C1	120.0(7)
C1-O1-Ni1	128.2(4)	C5-C6-C7	118.3(8)
C7-N1-Ni1	126.9(5)	C7-C6-C1	121.6(6)
C7-N1-C8	119.5(6)	N1-C7-C6	125.6(7)
C8-N1-Ni1	113.6(5)	C81-C8-N1	107.5(5)
O1-C1-C2	119.7(6)	O2-C9-C2	116.1(6)
O1-C1-C6	123.0(6)	O3-C9-O2	119.4(7)
C2-C1-C6	117.2(7)	O3-C9-C2	124.4(7)
C1-C2-C9	122.8(5)		

^{1} ½ – x, + y, 1 – z

**D-H···A**	**d(D-H)/Å**	**d(H···A)/Å**	**d(D-A)/Å**	**D-H···A/°**	**Symmetry code**
O2-H···O1	0.82	1.76	2.512(5)	152	-
C8-H···O2^{1}	0.97	2.51	3.295(7)	137.7	+ x, – 1 + y, + z

In molecular complex **b3** [NiH_2L_5] the Ni(II) is four-coordinated, Ni^{2+} atom exhibited sligthly distorted N_2O_2 square planes coordination provided by $H_2L_5^{2-}$ unit, which plays the role of tetra-dentate ligand.

Table S7. Bond distances (Å), angles (°) and hydrogen bonds for **b4**

Pt1-Cl1	2.320(4)	N2-C10	1.466(4)
Pt1-O3	2.015(10)	C1-C2	1.3978(18)
Pt1-N1	1.954(6)	C1-C6	1.3978(18)
Pt1-N2	2.060(6)	C2-C3	1.3978(18)
O1-C11	1.313(15)	C2-C11	1.467(5)
O2-C11	1.191(16)	C3-C4	1.3978(18)
O3-C1	1.310(10)	C4-C5	1.3978(18)
N1-C7	1.277(8)	C5-C6	1.3978(18)
N1-C8	1.466(4)	C6-C7	1.467(5)
N2-C9	1.466(4)	C8-C9	1.504(13)

O3-Pt1-Cl1	87.6(3)	C1-C2-C11	124.3(6)

O3-Pt1-N2	176.5(3)	C3-C2-C1	119.9(3)
N1-Pt1-Cl1	178.7(3)	C3-C2-C11	115.7(6)
N1-Pt1-O3	92.6(3)	C4-C3-C2	122.4(3)
N1-Pt1-N2	84.32(19)	C5-C4-C3	115.8(4)
N2-Pt1-Cl1	95.6(2)	C4-C5-C6	123.1(4)
C1-O3-Pt1	125.1(6)	C1-C6-C5	119.2(3)
C7-N1-Pt1	126.5(4)	C1-C6-C7	125.3(3)
C7-N1-C8	120.3(5)	C5-C6-C7	115.4(3)
C8-N1-Pt1	113.2(4)	N1-C7-C6	124.9(5)
C9-N2-Pt1	107.4(5)	N1-C8-C9	108.7(5)
C10-N2-Pt1	118.5(5)	N2-C9-C8	110.5(6)
C10-N2-C9	108.0(8)	O1-C11-C2	115.8(9)
O3-C1-C2	116.7(4)	O2-C11-O1	120.7(10)
O3-C1-C6	124.0(5)	O2-C11-C2	123.4(12)

**D-H···A**	**d(D-H)/Å**	**d(H···A)/Å**	**d(D-A)/Å**	**D-H···A/°**	**Symmetry code**
O1-H···O3	0.82	1.72	2.465(13)	150.5	-
N2-H···Cl1[1]	0.98	2.56	3.439(7)	149.3	$\frac{1}{2}+x, \frac{1}{2}-y, +z$
C7-H···O1[2]	0.93	2.43	3.283(11)	152.9	$+x, -1+y, +z$
C8-H···O1[2]	0.97	2.42	3.275(12)	147.3	$+x, -1+y, +z$
C8-H···Cl1[1]	0.97	2.96	3.699(9)	133.8	$\frac{1}{2}+x, \frac{1}{2}-y, +z$
C9-H···Cl1[3]	0.97	2.81	3.717(10)	156.6	$-\frac{1}{2}+x, \frac{1}{2}-y, +z$
C10-H···Cl1	0.96	2.75	3.367(13)	123.1	-

Single crystal X-ray difraction has releaved that compound **b4** has a molecular crystal structure. Its asymmetric unit comprises one complex molecule [PtH_2L_6Cl]. Pt^{2+} atom exhibited sligthly distorted N_2OCl square planes coordination provided by $H_2L_6^-$ unit, which plays the role of three-dentate ligand.

Table S8. Bond distances (Å), angles (°) and hydrogen bonds for **c2**

Cl1-C2	1.736(2)	C2-C3	1.382(4)
Cl2-C4	1.739(3)	C3-C4	1.380(4)
O1-C1	1.355(3)	C4-C5	1.377(4)
N1-C7	1.475(3)	C5-C6	1.385(3)
N1-C8	1.462(3)	C6-C7	1.507(3)
C1-C2	1.391(3)	C7-C7[1]	1.543(5)
C1-C6	1.408(3)	C8-C8[1]	1.510(6)

C8-N1-C7	112.87(18)	C5-C4-C3	121.3(2)
O1-C1-C2	118.9(2)	C4-C5-C6	120.7(2)
O1-C1-C6	122.4(2)	C1-C6-C7	119.8(2)
C2-C1-C6	118.7(2)	C5-C6-C1	119.0(2)
C1-C2-Cl1	118.4(2)	C5-C6-C7	121.1(2)

C3-C2-Cl1	119.42(19)	N1-C7-C6	109.34(17)
C3-C2-C1	122.1(2)	N1-C7-C7[1]	109.91(15)
C4-C3-C2	118.1(2)	C6-C7-C7[1]	109.86(17)
C3-C4-Cl2	119.0(2)	N1-C8-C8[1]	108.90(19)
C5-C4-Cl2	119.7(2)		

[1] $^3/_2 - x, +y, 1-z$

D-H···A	d(D-H)/Å	d(H···A)/Å	d(D-A)/Å	D-H···A/°	Symmetry code
O1-H···N1	0.82	1.9	2.613(3)	145.6	-
N1-H···Cl1[1]	0.77(3)	2.97(3)	3.616(2)	144(3)	$½ + x, 1 - y, + z$
N1-H···O1[1]	0.77(3)	2.64(3)	3.215(3)	133(3)	$½ + x, 1 - y, + z$

The results of the X-ray analysis on the synglecrystal, for the compounds **c2** and **c3**, showed that the fragment containing En cyclizes, at the level of the C=C bond, with the breaking of the double bond and the formation of a protonated piperazine fragment, the positive charge being counterbalanced by the negative charge of the phenolic O atom. A hydrogen bridge, N1-H···O1, is thus formed, through which the proton can migrate between O1 and N1, forming a 6-membered ring that gives stability to the compound.

Table S9. Bond distances (Å), angles (°) and hydrogen bonds for **c3**

Br1-C2	1.895(3)	C2-C3	1.388(4)
Br2-C4	1.896(3)	C3-C4	1.383(4)
O1-C1	1.365(3)	C4-C5	1.377(4)
N1-C7	1.479(4)	C5-C6	1.396(4)
N1-C8	1.470(4)	C6-C7	1.508(4)
C1-C2	1.389(4)	C7-C7[1]	1.553(5)
C1-C6	1.404(4)	C8-C8[1]	1.501(6)

C8-N1-C7	112.4(2)	C5-C4-C3	121.5(3)
O1-C1-C2	119.2(3)	C4-C5-C6	120.5(3)
O1-C1-C6	121.8(3)	C1-C6-C7	120.4(3)
C2-C1-C6	119.0(3)	C5-C6-C1	119.0(3)
C1-C2-Br1	118.4(2)	C5-C6-C7	120.4(3)
C3-C2-Br1	119.5(2)	N1-C7-C6	109.4(2)
C3-C2-C1	122.1(3)	N1-C7-C7[1]	109.84(18)
C4-C3-C2	117.9(3)	C6-C7-C7[1]	109.2(2)
C3-C4-Br2	119.1(2)	N1-C8-C8[1]	108.6(2)
C5-C4-Br2	119.4(3)		

[1] $½ - x, +y, 1 - z$

D-H···A	d(D-H)/Å	d(H···A)/Å	d(D-A)/Å	D-H···A/°	Symmetry code
O1-H···N1	0.79(3)	1.91(3)	2.618(3)	148(3)	-
N1-H···Br1[1]	0.77(3)	3.02(3)	3.669(3)	144(3)	$-½ + x, 1 - y, + z$

C8-H···Br1[2]	0.99	3.1	3.771(3)	126.1	$1-x, -½+y, 3/2-z$
C8-H···Br2[3]	0.99	3.05	3.931(3)	149	$+x, -1+y, +z$

S5. Computational calculations

Table S10. Wiberg bond order for AEAMDS

Vacuum		Water		Methanol	
**Bond**	**bond order**	**Bond**	**bond order**	**Bond**	**bond order**
1(Si)-2(O)	1.12912066	1(Si)-2(O)	1.12373283	1(Si)-2(O)	1.12159698
1(Si)-15(C)	0.95401237	1(Si)-15(C)	0.95267512	1(Si)-15(C)	0.95416892
1(Si)-19(C)	0.9521031	1(Si)-19(C)	0.95137094	1(Si)-19(C)	0.95071275
1(Si)-22(H)	0.0562742	1(Si)-22(H)	0.05611163	1(Si)-22(H)	0.05620549
1(Si)-23(C)	0.90921598	1(Si)-23(C)	0.91428017	1(Si)-23(C)	0.9144618
1(Si)-24(H)	0.05013429	1(Si)-24(H)	0.05120158	1(Si)-24(H)	0.05136584
1(Si)-25(H)	0.05628438	1(Si)-25(H)	0.05675983	1(Si)-25(H)	0.05662647
1(Si)-28(N)	0.07705491	1(Si)-28(N)	0.07836969	1(Si)-28(N)	0.07798263
2(O)-3(Si)	1.12911869	2(O)-3(Si)	1.12346145	2(O)-3(Si)	1.12159661
3(Si)-4(C)	0.95210196	3(Si)-4(C)	0.9512968	3(Si)-4(C)	0.95070465
3(Si)-8(C)	0.9540146	3(Si)-8(C)	0.95288486	3(Si)-8(C)	0.95419926
3(Si)-12(C)	0.90921563	3(Si)-12(C)	0.91442091	3(Si)-12(C)	0.91443959
3(Si)-13(H)	0.05628428	3(Si)-13(H)	0.05690831	3(Si)-13(H)	0.05662571
3(Si)-26(N)	0.07705417	3(Si)-26(N)	0.07722959	3(Si)-26(N)	0.07795215
4(C)-5(H)	0.95948998	4(C)-5(H)	0.96060379	4(C)-5(H)	0.96008495
4(C)-6(H)	0.96428853	4(C)-6(H)	0.96337017	4(C)-6(H)	0.96311081
4(C)-7(H)	0.96300778	4(C)-7(H)	0.96293445	4(C)-7(H)	0.96267356
8(C)-9(H)	0.96013582	8(C)-9(H)	0.95939373	8(C)-9(H)	0.95997508
8(C)-10(H)	0.96291704	8(C)-10(H)	0.96288552	8(C)-10(H)	0.96273022
8(C)-11(H)	0.9591975	8(C)-11(H)	0.9583451	8(C)-11(H)	0.95876947
12(C)-13(H)	0.94428689	12(C)-13(H)	0.94277417	12(C)-13(H)	0.94302828
12(C)-14(H)	0.93852368	12(C)-14(H)	0.93744953	12(C)-14(H)	0.93748504
12(C)-26(N)	1.11194169	12(C)-26(N)	1.11218595	12(C)-26(N)	1.11156817
12(C)-27(H)	0.05910119	12(C)-27(H)	0.0591818	12(C)-27(H)	0.05910127
15(C)-16(H)	0.96291725	15(C)-16(H)	0.96276372	15(C)-16(H)	0.96272758
15(C)-17(H)	0.95919778	15(C)-17(H)	0.95828657	15(C)-17(H)	0.95876739
15(C)-18(H)	0.96013549	15(C)-18(H)	0.95936908	15(C)-18(H)	0.95996537
19(C)-20(H)	0.96428864	19(C)-20(H)	0.96350495	19(C)-20(H)	0.96311565
19(C)-21(H)	0.96300755	19(C)-21(H)	0.96295728	19(C)-21(H)	0.96268411
19(C)-22(H)	0.95949048	19(C)-22(H)	0.96072217	19(C)-22(H)	0.96009537
23(C)-24(H)	0.93852391	23(C)-24(H)	0.93755166	23(C)-24(H)	0.93748684
23(C)-25(H)	0.94428654	23(C)-25(H)	0.94294927	23(C)-25(H)	0.94302992
23(C)-28(N)	1.11194184	23(C)-28(N)	1.1114109	23(C)-28(N)	1.11154135
23(C)-29(H)	0.05910121	23(C)-29(H)	0.05907016	23(C)-29(H)	0.05909873
23(C)-36(C)	0.07080552	23(C)-36(C)	0.07076186	23(C)-36(C)	0.0706528
26(N)-27(H)	1.02358939	26(N)-27(H)	1.02182672	26(N)-27(H)	1.02197713
26(N)-30(C)	1.12175731	26(N)-30(C)	1.11917552	26(N)-30(C)	1.11905663

28(N)-29(H)	1.02358954	28(N)-29(H)	1.02199728	28(N)-29(H)	1.02198036
28(N)-36(C)	1.12175695	28(N)-36(C)	1.1188779	28(N)-36(C)	1.11905769
30(C)-31(H)	0.92189702	30(C)-31(H)	0.92205331	30(C)-31(H)	0.92204204
30(C)-32(H)	0.9236495	30(C)-32(H)	0.92506936	30(C)-32(H)	0.92496525
30(C)-33(C)	1.03432722	30(C)-33(C)	1.03610674	30(C)-33(C)	1.03610791
33(C)-34(H)	0.93195804	33(C)-34(H)	0.93199306	33(C)-34(H)	0.93203229
33(C)-35(H)	0.93501514	33(C)-35(H)	0.93631157	33(C)-35(H)	0.93621492
33(C)-45(N)	1.1411093	33(C)-45(N)	1.13651988	33(C)-45(N)	1.13659617
36(C)-37(H)	0.92364969	36(C)-37(H)	0.92509808	36(C)-37(H)	0.92497498
36(C)-38(H)	0.92189717	36(C)-38(H)	0.92200028	36(C)-38(H)	0.92204188
36(C)-39(C)	1.03432735	36(C)-39(C)	1.03619087	36(C)-39(C)	1.03610749
39(C)-40(H)	0.93195776	39(C)-40(H)	0.93203037	39(C)-40(H)	0.93202792
39(C)-41(H)	0.93501508	39(C)-41(H)	0.93629449	39(C)-41(H)	0.93621615
39(C)-42(N)	1.14111006	39(C)-42(N)	1.13642954	39(C)-42(N)	1.1365999
42(N)-43(H)	1.04469216	42(N)-43(H)	1.04234142	42(N)-43(H)	1.04239486
42(N)-44(H)	1.04648043	42(N)-44(H)	1.04424438	42(N)-44(H)	1.04432173
45(N)-46(H)	1.04469214	45(N)-46(H)	1.04232179	45(N)-46(H)	1.04239609
45(N)-47(H)	1.04648071	45(N)-47(H)	1.04424507	45(N)-47(H)	1.04432248

Table S11. Second Order Perturbation Theory Analysis of Fock Matrix in NBO Basis for:

AEAMDS - vacuum

Donor NBO(i)	Acceptor NBO(j)	E(2)
BD(1)C12-H13	BD*(1)N26-C30	3.26
BD(1)C12-N26	BD*(1)C30-C33	1.84
BD(1)C15-H17	BD*(1)Si1-C23	1.58
BD(1)C15-H18	BD*(1)Si1-O2	1.75
BD(1)Si1-C15	BD*(1)Si1-C23	1.54
BD(1)C19-H22	BD*(1)Si1-O2	1.94
BD(1)C23-H25	BD*(1)Si1-C15	1.53
BD(1)C23-H25	BD*(1)N28-C36	3.26
BD(1)C23-N28	BD*(1)C36-C39	1.84
BD(1)N26-H27	BD*(1)Si3-C12	1.83
BD(1)N26-H27	BD*(1)C30-H32	2.45
BD(1)N26-C30	BD*(1)C33-N45	1.58
BD(1)N28-H29	BD*(1)Si1-C23	1.83
BD(1)N28-H29	BD*(1)C36-H37	2.45
BD(1)N28-C36	BD*(1)C39-N42	1.58
BD(1)C30-H31	BD*(1)C33-H35	2.45
BD(1)C30-H32	BD*(1)N26-H27	3.03
BD(1)C30-H32	BD*(1)C33-H34	2.69
BD(1)C30-C33	BD*(1)C12-N26	3.2
BD(1)C30-C33	BD*(1)N45-H46	1.8
BD(1)C33-H34	BD*(1)C30-H32	2.5
BD(1)C33-H35	BD*(1)C30-H31	2.88
BD(1)C33-H35	BD*(1)N45-H47	2.99
BD(1)C33-N45	BD*(1)N26-C30	1.91
BD(1)C36-H37	BD*(1)N28-H29	3.03
BD(1)C36-H37	BD*(1)C39-H40	2.69
BD(1)C36-H38	BD*(1)C39-H41	2.45
BD(1)C36-C39	BD*(1)C23-N28	3.2
BD(1)C36-C39	BD*(1)N42-H43	1.8
BD(1)C39-H40	BD*(1)C36-H37	2.5
BD(1)C39-H41	BD*(1)C36-H38	2.88
BD(1)C39-H41	BD*(1)N42-H44	2.99
BD(1)C39-N42	BD*(1)N28-C36	1.91
BD(1)N42-H43	BD*(1)C36-C39	2.62
BD(1)N42-H44	BD*(1)C39-H41	2.12
BD(1)N45-H46	BD*(1)C30-C33	2.62
BD(1)N45-H47	BD*(1)C33-H35	2.12
CR(2)Si1	BD*(1)Si1-O2	2.28
CR(2)Si1	BD*(1)O2-Si3	2.4
CR(2)Si3	BD*(1)Si1-O2	2.4
CR(2)Si3	BD*(1)O2-Si3	2.28
BD(1)Si1-C19	BD*(1)Si1-O2	2.92
LP(1)O2	RY*(2)Si1	1.64
LP(1)O2	RY*(2)Si3	1.62
LP(1)O2	BD*(1)Si1-C19	5.4
LP(1)O2	BD*(1)Si1-C23	5.35
LP(1)O2	BD*(1)Si3-C4	5.48

LP(1)O2	BD*(1)Si3-C12	5.28
LP(2)O2	RY*(1)Si1	1.69
LP(2)O2	RY*(1)Si3	1.68
LP(2)O2	BD*(1)Si1-C15	7.31
LP(2)O2	BD*(1)Si1-C19	2.18
LP(2)O2	BD*(1)Si1-C23	1.61
LP(2)O2	BD*(1)Si3-C4	2.1
LP(2)O2	BD*(1)Si3-C8	7.32
LP(2)O2	BD*(1)Si3-C12	1.68
LP(1)N26	RY*(2)H27	1.5
LP(1)N26	BD*(1)C12-H14	6.35
LP(1)N26	BD*(1)C30-H31	8.16
LP(1)N28	RY*(2)H29	1.5
LP(1)N28	BD*(1)C23-H24	6.35
LP(1)N28	BD*(1)C36-H38	8.16
LP(1)N42	BD*(1)C39-H40	7.87
LP(1)N45	BD*(1)C33-H34	7.87
BD(1)Si1-C23	RY*(1)N28	1.85
BD(1)Si1-C23	BD*(1)Si1-O2	2.8
BD(1)Si1-C23	BD*(1)N28-H29	2.11
BD(1)O2-Si3	BD*(1)Si1-O2	1.94
BD(1)Si1-O2	BD*(1)O2-Si3	1.94
BD(1)Si3-C4	BD*(1)O2-Si3	2.93
BD(1)Si3-C8	BD*(1)O2-Si3	2.74
BD(1)Si3-C8	BD*(1)Si3-C12	1.54
BD(1)Si3-C12	RY*(1)N26	1.85
BD(1)Si3-C12	BD*(1)O2-Si3	2.8
BD(1)Si3-C12	BD*(1)N26-H27	2.11
BD(1)Si1-C15	BD*(1)Si1-O2	2.74
BD(1)C4-H5	BD*(1)O2-Si3	1.94
BD(1)C8-H9	BD*(1)O2-Si3	1.75
BD(1)C8-H11	BD*(1)Si3-C12	1.58
BD(1)C12-H13	BD*(1)Si3-C8	1.53

AEAMDS - water

Donor NBO(i)	Acceptor NBO(j)	E(2)
BD(1)C15-H18	BD*(1)Si1-O2	1.81
BD(1)C19-H22	BD*(1)Si1-O2	1.89
BD(1)C23-H25	BD*(1)Si1-C15	1.59
BD(1)C23-H25	BD*(1)N28-C36	3.25
BD(1)Si1-C15	BD*(1)Si1-C23	1.5
BD(1)C23-N28	BD*(1)C36-C39	1.86
BD(1)N26-H27	BD*(1)Si3-C12	1.99
BD(1)N26-H27	BD*(1)C30-H32	2.51
BD(1)N26-C30	BD*(1)C33-N45	1.6
BD(1)N28-H29	BD*(1)Si1-C23	1.96
BD(1)N28-H29	BD*(1)C36-H37	2.51
BD(1)N28-C36	BD*(1)C39-N42	1.6
BD(1)C30-H31	BD*(1)C33-H35	2.49
BD(1)C30-H32	BD*(1)N26-H27	2.85
BD(1)C30-H32	BD*(1)C33-H34	2.61
BD(1)C30-C33	BD*(1)C12-N26	3.14
BD(1)C30-C33	BD*(1)N45-H46	1.65
BD(1)C33-H34	BD*(1)C30-H32	2.58
BD(1)C33-H35	BD*(1)C30-H31	2.82
BD(1)C33-H35	BD*(1)N45-H47	2.77
BD(1)C33-N45	BD*(1)N26-C30	1.96
BD(1)C36-H37	BD*(1)N28-H29	2.85
BD(1)C36-H37	BD*(1)C39-H40	2.61
BD(1)C36-H38	BD*(1)C39-H41	2.49
BD(1)C36-C39	BD*(1)C23-N28	3.13
BD(1)C36-C39	BD*(1)N42-H43	1.65
BD(1)C39-H40	BD*(1)C36-H37	2.58
BD(1)C39-H41	BD*(1)C36-H38	2.83
BD(1)C39-H41	BD*(1)N42-H44	2.77
BD(1)C39-N42	BD*(1)N28-C36	1.97
BD(1)N42-H43	BD*(1)C36-C39	2.69
BD(1)N42-H44	BD*(1)C39-H41	2.17
BD(1)N45-H46	BD*(1)C30-C33	2.69
BD(1)N45-H47	BD*(1)C33-H35	2.17
CR(2)Si1	BD*(1)Si1-O2	2.32
CR(2)Si1	BD*(1)O2-Si3	2.25
CR(2)Si3	BD*(1)Si1-O2	2.24
CR(2)Si3	BD*(1)O2-Si3	2.31
BD(1)Si1-C19	BD*(1)Si1-O2	2.87
LP(1)O2	RY*(2)Si1	1.58
LP(1)O2	RY*(2)Si3	1.68
LP(1)O2	BD*(1)Si1-C15	3.43
LP(1)O2	BD*(1)Si1-C23	5.63
LP(1)O2	BD*(1)Si3-C8	2.48
LP(1)O2	BD*(1)Si3-C12	5.86
LP(2)O2	RY*(1)Si1	1.77
LP(2)O2	RY*(1)Si3	1.84
LP(2)O2	BD*(1)Si1-C15	3.98
LP(2)O2	BD*(1)Si1-C19	6.77
LP(2)O2	BD*(1)Si3-C4	6.12
LP(2)O2	BD*(1)Si3-C8	4.96
LP(1)N26	BD*(1)C12-H14	6.39
LP(1)N26	BD*(1)C30-H31	7.72
LP(1)N28	BD*(1)C23-H24	6.29
LP(1)N28	BD*(1)C36-H38	7.7
LP(1)N42	BD*(1)C39-H40	7.23
LP(1)N45	BD*(1)C33-H34	7.23

BD(1)Si1-C23	RY*(1)N28	1.79
BD(1)Si1-C23	BD*(1)Si1-O2	2.68
BD(1)Si1-C23	BD*(1)N28-H29	2.03
BD(1)O2-Si3	BD*(1)Si1-O2	1.83
BD(1)Si3-C4	BD*(1)O2-Si3	2.91
BD(1)Si1-O2	BD*(1)O2-Si3	1.83
BD(1)Si3-C8	BD*(1)O2-Si3	3.01
BD(1)Si3-C8	BD*(1)Si3-C12	1.51
BD(1)Si3-C12	RY*(1)N26	1.76
BD(1)Si3-C12	BD*(1)O2-Si3	2.69
BD(1)Si3-C12	BD*(1)N26-H27	2.05
BD(1)C4-H5	BD*(1)O2-Si3	1.9
BD(1)C8-H9	BD*(1)O2-Si3	1.8
BD(1)Si1-C15	BD*(1)Si1-O2	3.05
BD(1)C8-H11	BD*(1)Si3-C12	1.6
BD(1)C12-H13	BD*(1)Si3-C8	1.61
BD(1)C12-H13	BD*(1)N26-C30	3.3
BD(1)C12-N26	BD*(1)C30-C33	1.85
BD(1)C15-H17	BD*(1)Si1-C23	1.6

AEAMDS - methanol

Donor NBO(i)	Acceptor NBO(j)	E(2)
BD(1)Si1-C15	BD*(1)Si1-O2	2.76
BD(1)C12-N26	BD*(1)C30-C33	1.85
BD(1)C15-H17	BD*(1)Si1-C23	1.59
BD(1)C15-H18	BD*(1)Si1-O2	1.77
BD(1)C19-H22	BD*(1)Si1-O2	1.92
BD(1)Si1-C15	BD*(1)Si1-C19	1.5
BD(1)C23-H25	BD*(1)Si1-C15	1.59
BD(1)C23-H25	BD*(1)N28-C36	3.26
BD(1)C23-N28	BD*(1)C36-C39	1.85
BD(1)N26-H27	BD*(1)Si3-C12	1.97
BD(1)Si1-C15	BD*(1)Si1-C23	1.54
BD(1)N26-H27	BD*(1)C30-H32	2.51
BD(1)N26-C30	BD*(1)C33-N45	1.6
BD(1)N28-H29	BD*(1)Si1-C23	1.96
BD(1)N28-H29	BD*(1)C36-H37	2.51
BD(1)N28-C36	BD*(1)C39-N42	1.6
BD(1)C30-H31	BD*(1)C33-H35	2.49
BD(1)C30-H32	BD*(1)N26-H27	2.86
BD(1)C30-H32	BD*(1)C33-H34	2.61
BD(1)C30-C33	BD*(1)C12-N26	3.14
BD(1)C30-C33	BD*(1)N45-H46	1.66
BD(1)C33-H34	BD*(1)C30-H32	2.58
BD(1)C33-H35	BD*(1)C30-H31	2.83
BD(1)C33-H35	BD*(1)N45-H47	2.78
BD(1)C33-N45	BD*(1)N26-C30	1.96

BD(1)C36-H37	BD*(1)N28-H29	2.86
BD(1)C36-H37	BD*(1)C39-H40	2.61
BD(1)C36-H38	BD*(1)C39-H41	2.49
BD(1)C36-C39	BD*(1)C23-N28	3.14
BD(1)C36-C39	BD*(1)N42-H43	1.66
BD(1)C39-H40	BD*(1)C36-H37	2.58
BD(1)C39-H41	BD*(1)C36-H38	2.83
BD(1)C39-H41	BD*(1)N42-H44	2.78
BD(1)C39-N42	BD*(1)N28-C36	1.96
BD(1)N42-H43	BD*(1)C36-C39	2.69
BD(1)N42-H44	BD*(1)C39-H41	2.17
BD(1)N45-H46	BD*(1)C30-C33	2.69
BD(1)N45-H47	BD*(1)C33-H35	2.17
CR(2)Si1	BD*(1)Si1-O2	2.31
CR(2)Si1	BD*(1)O2-Si3	2.25
CR(2)Si3	BD*(1)Si1-O2	2.25
CR(2)Si3	BD*(1)O2-Si3	2.31
LP(1)O2	RY*(2)Si1	1.59
LP(1)O2	RY*(2)Si3	1.58
LP(1)O2	BD*(1)Si1-C19	5.41
LP(1)O2	BD*(1)Si1-C23	4.47
LP(1)O2	BD*(1)Si3-C4	5.51
LP(1)O2	BD*(1)Si3-C12	4.39
LP(2)O2	RY*(1)Si1	1.7
LP(2)O2	RY*(1)Si3	1.68
LP(2)O2	BD*(1)Si1-C15	7.28
LP(2)O2	BD*(1)Si1-C19	1.96
LP(2)O2	BD*(1)Si1-C23	1.72
BD(1)Si1-C19	BD*(1)Si1-O2	3.12
LP(2)O2	BD*(1)Si3-C4	1.86
LP(2)O2	BD*(1)Si3-C8	7.28
LP(2)O2	BD*(1)Si3-C12	1.82
LP(1)N26	BD*(1)C12-H14	6.32
LP(1)N26	BD*(1)C30-H31	7.73
LP(1)N28	BD*(1)C23-H24	6.31
LP(1)N28	BD*(1)C36-H38	7.73
LP(1)N42	BD*(1)C39-H40	7.25
LP(1)N45	BD*(1)C33-H34	7.25
BD(1)Si1-C23	RY*(1)N28	1.78
BD(1)Si1-C23	BD*(1)Si1-O2	2.73
BD(1)Si1-C23	BD*(1)N28-H29	2.03
BD(1)O2-Si3	BD*(1)Si1-O2	1.85
BD(1)Si3-C4	BD*(1)O2-Si3	3.12
BD(1)Si1-O2	BD*(1)O2-Si3	1.85
BD(1)Si3-C8	BD*(1)O2-Si3	2.75
BD(1)Si3-C8	BD*(1)Si3-C4	1.5

BD(1)Si3-C8	BD*(1)Si3-C12	1.54
BD(1)Si3-C12	RY*(1)N26	1.78
BD(1)Si3-C12	BD*(1)O2-Si3	2.73
BD(1)Si3-C12	BD*(1)N26-H27	2.04
BD(1)C4-H5	BD*(1)O2-Si3	1.92
BD(1)C8-H9	BD*(1)O2-Si3	1.77
BD(1)C8-H11	BD*(1)Si3-C12	1.59
BD(1)C12-H13	BD*(1)Si3-C8	1.59
BD(1)C12-H13	BD*(1)N26-C30	3.27

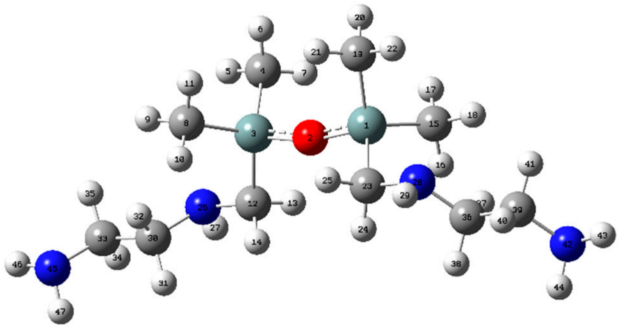

Figure S5. AEAMDS – Atoms labels

Table S12. Wiberg bond order for APTMDS

Vacuum		Water		Methanol	
**Bond**	**bond order**	**Bond**	**bond order**	**Bond**	**bond order**
1(Si)-2(O)	1.12976248	1(Si)-2(O)	1.1201774	1(Si)-2(O)	1.12063325
1(Si)-4(C)	0.95079672	1(Si)-4(C)	0.95279289	1(Si)-4(C)	0.9526888
1(Si)-12(C)	0.94823757	1(Si)-12(C)	0.94910284	1(Si)-12(C)	0.94902269
1(Si)-20(C)	0.91582389	1(Si)-20(C)	0.91594023	1(Si)-20(C)	0.91594935
1(Si)-21(H)	0.05380026	1(Si)-21(H)	0.0538076	1(Si)-21(H)	0.05380094
1(Si)-22(H)	0.05436743	1(Si)-22(H)	0.05428096	1(Si)-22(H)	0.0542905
1(Si)-32(C)	0.06039288	1(Si)-32(C)	0.06072579	1(Si)-32(C)	0.06072583
2(O)-3(Si)	1.12903873	2(O)-3(Si)	1.12003204	2(O)-3(Si)	1.12042926
3(Si)-8(C)	0.94777339	3(Si)-8(C)	0.94915984	3(Si)-8(C)	0.9490949
3(Si)-16(C)	0.950709	3(Si)-16(C)	0.9526878	3(Si)-16(C)	0.95255628
3(Si)-23(C)	0.91623915	3(Si)-23(C)	0.91598705	3(Si)-23(C)	0.91600624
3(Si)-26(C)	0.06070231	3(Si)-26(C)	0.06072331	3(Si)-26(C)	0.06073963
4(C)-5(H)	0.96319916	4(C)-5(H)	0.96201015	4(C)-5(H)	0.96208915
4(C)-6(H)	0.96274985	4(C)-6(H)	0.96286937	4(C)-6(H)	0.96286361
4(C)-7(H)	0.9633855	4(C)-7(H)	0.96328781	4(C)-7(H)	0.96329229
8(C)-9(H)	0.96321386	8(C)-9(H)	0.96307636	8(C)-9(H)	0.96310852
8(C)-10(H)	0.96301838	8(C)-10(H)	0.96281157	8(C)-10(H)	0.96283626
8(C)-11(H)	0.96283981	8(C)-11(H)	0.96162788	8(C)-11(H)	0.96171637
12(C)-13(H)	0.96246126	12(C)-13(H)	0.96137986	12(C)-13(H)	0.96142664
12(C)-14(H)	0.96290224	12(C)-14(H)	0.96285111	12(C)-14(H)	0.96281655
12(C)-15(H)	0.96245949	12(C)-15(H)	0.96267028	12(C)-15(H)	0.96264487
16(C)-17(H)	0.96264075	16(C)-17(H)	0.96274176	16(C)-17(H)	0.96272623
16(C)-18(H)	0.9631659	16(C)-18(H)	0.9632806	16(C)-18(H)	0.96327683
16(C)-19(H)	0.96307434	16(C)-19(H)	0.96189167	16(C)-19(H)	0.96196829
20(C)-21(H)	0.93127735	20(C)-21(H)	0.93337519	20(C)-21(H)	0.93331779

20(C)-22(H)	0.93674198	20(C)-22(H)	0.936266	20(C)-22(H)	0.9363105
20(C)-32(C)	1.04676952	20(C)-32(C)	1.04575545	20(C)-32(C)	1.04580266
23(C)-24(H)	0.93631417	23(C)-24(H)	0.93562327	23(C)-24(H)	0.93564601
23(C)-25(H)	0.93138931	23(C)-25(H)	0.9337759	23(C)-25(H)	0.93365162
23(C)-26(C)	1.04663244	23(C)-26(C)	1.04570371	23(C)-26(C)	1.0457166
26(C)-27(H)	0.92510686	26(C)-27(H)	0.92463792	26(C)-27(H)	0.92466395
26(C)-28(H)	0.92579	26(C)-28(H)	0.92454715	26(C)-28(H)	0.924605
26(C)-29(C)	1.03368541	26(C)-29(C)	1.03517569	26(C)-29(C)	1.03511344
29(C)-30(H)	0.93535776	29(C)-30(H)	0.93583925	29(C)-30(H)	0.93581527
29(C)-31(H)	0.93365008	29(C)-31(H)	0.93321947	29(C)-31(H)	0.93323961
29(C)-41(N)	1.13691549	29(C)-41(N)	1.13321965	29(C)-41(N)	1.13336348
32(C)-33(H)	0.92515596	32(C)-33(H)	0.92473963	32(C)-33(H)	0.92475761
32(C)-34(H)	0.92577368	32(C)-34(H)	0.92435067	32(C)-34(H)	0.92441244
32(C)-35(C)	1.03378801	32(C)-35(C)	1.03526637	32(C)-35(C)	1.03522201
35(C)-36(H)	0.93367273	35(C)-36(H)	0.93319791	35(C)-36(H)	0.9332133
35(C)-37(H)	0.93541088	35(C)-37(H)	0.9358462	35(C)-37(H)	0.93582903
35(C)-38(N)	1.13675534	35(C)-38(N)	1.13330663	35(C)-38(N)	1.13345907
38(N)-39(H)	1.04604726	38(N)-39(H)	1.04391624	38(N)-39(H)	1.04397848
38(N)-40(H)	1.04534423	38(N)-40(H)	1.0432803	38(N)-40(H)	1.0433405
41(N)-42(H)	1.04535605	41(N)-42(H)	1.04330661	41(N)-42(H)	1.04336211
41(N)-43(H)	1.04615228	41(N)-43(H)	1.0439534	41(N)-43(H)	1.04402796

Table S13. Second Order Perturbation Theory Analysis of Fock Matrix in NBO Basis for:

APTMDS - vacuum

Donor NBO(i)	Acceptor NBO(j)	E(2)
BD(1)C20-C32	RY*(1)C35	1.52
BD(1)C23-C26	RY*(1)C29	1.52
BD(1)Si1-C4	BD*(1)Si1-O2	2.26
BD(1)C16-H19	BD*(1)O2-Si3	1.73
BD(1)C20-H21	BD*(1)C32-H34	3.02
BD(1)C20-H22	BD*(1)C32-H33	3.14
BD(1)C23-H24	BD*(1)C26-H27	3.14
BD(1)C23-H25	BD*(1)C26-H28	3.01
BD(1)C26-H27	BD*(1)C23-H24	2.83
BD(1)C26-H27	BD*(1)C29-H30	2.68
BD(1)C26-H28	BD*(1)C23-H25	2.7
BD(1)C26-H28	BD*(1)C29-N41	3.54
BD(1)C26-C29	BD*(1)Si3-C23	1.79
BD(1)C26-C29	BD*(1)N41-H42	1.78
BD(1)C29-H30	BD*(1)C26-H27	2.8
BD(1)C29-H30	BD*(1)N41-H43	2.97
BD(1)C29-H31	BD*(1)C23-C26	3.53
BD(1)C32-H33	BD*(1)C20-H22	2.82
BD(1)C32-H33	BD*(1)C35-H37	2.69
BD(1)C32-H34	BD*(1)C20-H21	2.71
BD(1)C32-H34	BD*(1)C35-N38	3.54
BD(1)C32-C35	BD*(1)Si1-C20	1.79
BD(1)C32-C35	BD*(1)N38-H40	1.78
BD(1)C35-H36	BD*(1)C20-C32	3.53
BD(1)C35-H37	BD*(1)C32-H33	2.79
BD(1)C35-H37	BD*(1)N38-H39	2.97
BD(1)N38-H39	BD*(1)C35-H37	2.13
BD(1)N38-H40	BD*(1)C32-C35	2.75
BD(1)N41-H42	BD*(1)C26-C29	2.75
BD(1)N41-H43	BD*(1)C29-H30	2.13
CR(2)Si1	BD*(1)Si1-O2	2.41
CR(2)Si1	BD*(1)O2-Si3	2.36
CR(2)Si3	BD*(1)Si1-O2	2.36
CR(2)Si3	BD*(1)O2-Si3	2.41
LP(1)O2	RY*(2)Si1	1.92
LP(1)O2	RY*(2)Si3	1.85
LP(1)O2	BD*(1)Si1-C4	5.85
LP(1)O2	BD*(1)Si1-C20	3.59
LP(1)O2	BD*(1)Si3-C16	5.17
LP(1)O2	BD*(1)Si3-C23	4.8
LP(2)O2	RY*(1)Si1	1.99

LP(2)O2	RY*(1)Si3	1.89
LP(2)O2	BD*(1)Si1-C12	7.07
LP(2)O2	BD*(1)Si1-C20	3.69
LP(2)O2	BD*(1)Si3-C8	7.45
LP(2)O2	BD*(1)Si3-C23	2.44
LP(1)N38	BD*(1)C35-H36	7.38
BD(1)Si1-C12	BD*(1)Si1-O2	2.65
LP(1)N41	BD*(1)C29-H31	7.39
BD(1)Si1-C20	BD*(1)Si1-O2	3.19
BD(1)Si1-C20	BD*(1)C32-C35	3.48
BD(1)O2-Si3	BD*(1)Si1-O2	1.94
BD(1)Si1-O2	BD*(1)O2-Si3	1.94
BD(1)Si3-C8	BD*(1)O2-Si3	2.6
BD(1)Si3-C16	BD*(1)O2-Si3	2.28
BD(1)Si3-C23	BD*(1)O2-Si3	3.23
BD(1)Si3-C23	BD*(1)C26-C29	3.48
BD(1)C4-H5	BD*(1)Si1-O2	1.74
BD(1)C8-H11	BD*(1)O2-Si3	1.77
BD(1)C12-H13	BD*(1)Si1-O2	1.78

APTMDS - water

BD(1)C20-C32	RY*(1)C35	1.52
BD(1)C23-C26	RY*(1)C29	1.52
BD(1)Si1-O2	RY*(7)Si3	1.55
BD(1)C12-H13	BD*(1)Si1-O2	1.84
BD(1)C16-H19	BD*(1)O2-Si3	1.78
BD(1)Si1-C4	BD*(1)Si1-O2	2.28
BD(1)C20-H21	BD*(1)C32-H34	2.92
BD(1)C20-H22	BD*(1)C32-H33	3.06
BD(1)C23-H24	BD*(1)C26-H27	3.05
BD(1)C23-H25	BD*(1)C26-H28	2.93
BD(1)C26-H27	BD*(1)C23-H24	2.88
BD(1)C26-H27	BD*(1)C29-H30	2.68
BD(1)C26-H28	BD*(1)C23-H25	2.84
BD(1)C26-H28	BD*(1)C29-N41	3.63
BD(1)C26-C29	BD*(1)Si3-C23	1.82
BD(1)C26-C29	BD*(1)N41-H42	1.64
BD(1)C29-H30	BD*(1)C26-H27	2.79
BD(1)C29-H30	BD*(1)N41-H43	2.79
BD(1)C29-H31	BD*(1)C23-C26	3.65
BD(1)C32-H33	BD*(1)C20-H22	2.86
BD(1)C32-H33	BD*(1)C35-H37	2.68
BD(1)C32-H34	BD*(1)C20-H21	2.86
BD(1)C32-H34	BD*(1)C35-N38	3.63
BD(1)C32-C35	BD*(1)Si1-C20	1.81
BD(1)C32-C35	BD*(1)N38-H40	1.64
BD(1)C35-H36	BD*(1)C20-C32	3.66
BD(1)C35-H37	BD*(1)C32-H33	2.79
BD(1)C35-H37	BD*(1)N38-H39	2.8
BD(1)N38-H39	BD*(1)C35-H37	2.2
BD(1)N38-H40	BD*(1)C32-C35	2.88
BD(1)N41-H42	BD*(1)C26-C29	2.88
BD(1)N41-H43	BD*(1)C29-H30	2.2
CR(2)Si1	BD*(1)Si1-O2	2.47
CR(2)Si1	BD*(1)O2-Si3	2.21
CR(2)Si3	BD*(1)Si1-O2	2.21
CR(2)Si3	BD*(1)O2-Si3	2.47
LP(1)O2	RY*(2)Si1	1.85
LP(1)O2	RY*(2)Si3	1.83
LP(1)O2	BD*(1)Si1-C4	4.91
LP(1)O2	BD*(1)Si1-C20	3.98
LP(1)O2	BD*(1)Si3-C16	4.6
LP(1)O2	BD*(1)Si3-C23	4.51
LP(2)O2	RY*(1)Si1	1.92
LP(2)O2	RY*(1)Si3	1.85
LP(2)O2	BD*(1)Si1-C12	7.22
LP(2)O2	BD*(1)Si1-C20	3.07
LP(2)O2	BD*(1)Si3-C8	7.36
LP(2)O2	BD*(1)Si3-C23	2.49
LP(1)N38	BD*(1)C35-H36	6.89
BD(1)Si1-C12	BD*(1)Si1-O2	2.71
LP(1)N41	BD*(1)C29-H31	6.89
BD(1)Si1-C20	BD*(1)Si1-O2	3.35
BD(1)Si1-C20	BD*(1)C32-C35	3.47
BD(1)O2-Si3	RY*(7)Si1	1.76
BD(1)O2-Si3	BD*(1)Si1-O2	1.83
BD(1)Si1-O2	BD*(1)O2-Si3	1.83
BD(1)Si3-C8	BD*(1)O2-Si3	2.68
BD(1)Si3-C16	BD*(1)O2-Si3	2.3
BD(1)Si3-C23	BD*(1)O2-Si3	3.37
BD(1)Si3-C23	BD*(1)C26-C29	3.46
BD(1)C4-H5	BD*(1)Si1-O2	1.79
BD(1)C8-H11	BD*(1)O2-Si3	1.85

APTMDS - methanol

**Donor NBO(i)**	**Acceptor NBO(j)**	**E(2)**
BD(1)C20-C32	RY*(1)C35	1.52
BD(1)C23-C26	RY*(1)C29	1.52
BD(1)Si1-O2	RY*(7)Si3	1.55
BD(1)C12-H13	BD*(1)Si1-O2	1.84
BD(1)C16-H19	BD*(1)O2-Si3	1.78
BD(1)Si1-C4	BD*(1)Si1-O2	2.28
BD(1)C20-H21	BD*(1)C32-H34	2.92
BD(1)C20-H22	BD*(1)C32-H33	3.06

BD(1)C23-H24	BD*(1)C26-H27	3.06
BD(1)C23-H25	BD*(1)C26-H28	2.93
BD(1)C26-H27	BD*(1)C23-H24	2.88
BD(1)C26-H27	BD*(1)C29-H30	2.67
BD(1)C26-H28	BD*(1)C23-H25	2.83
BD(1)C26-H28	BD*(1)C29-N41	3.62
BD(1)C26-C29	BD*(1)Si3-C23	1.81
BD(1)C26-C29	BD*(1)N41-H42	1.64
BD(1)C29-H30	BD*(1)C26-H27	2.79
BD(1)C29-H30	BD*(1)N41-H43	2.8
BD(1)C29-H31	BD*(1)C23-C26	3.65
BD(1)C32-H33	BD*(1)C20-H22	2.86
BD(1)C32-H33	BD*(1)C35-H37	2.68
BD(1)C32-H34	BD*(1)C20-H21	2.86
BD(1)C32-H34	BD*(1)C35-N38	3.63
BD(1)C32-C35	BD*(1)Si1-C20	1.81
BD(1)C32-C35	BD*(1)N38-H40	1.64
BD(1)C35-H36	BD*(1)C20-C32	3.65
BD(1)C35-H37	BD*(1)C32-H33	2.79
BD(1)C35-H37	BD*(1)N38-H39	2.8
BD(1)N38-H39	BD*(1)C35-H37	2.2
BD(1)N38-H40	BD*(1)C32-C35	2.88
BD(1)N41-H42	BD*(1)C26-C29	2.88
BD(1)N41-H43	BD*(1)C29-H30	2.2
CR(2)Si1	BD*(1)Si1-O2	2.46
CR(2)Si1	BD*(1)O2-Si3	2.22
CR(2)Si3	BD*(1)Si1-O2	2.22
CR(2)Si3	BD*(1)O2-Si3	2.47
LP(1)O2	RY*(2)Si1	1.87
LP(1)O2	RY*(2)Si3	1.82
LP(1)O2	BD*(1)Si1-C4	5
LP(1)O2	BD*(1)Si1-C20	3.87
LP(1)O2	BD*(1)Si3-C16	4.55
LP(1)O2	BD*(1)Si3-C23	4.63
LP(2)O2	RY*(1)Si1	1.93
LP(2)O2	RY*(1)Si3	1.85
LP(2)O2	BD*(1)Si1-C12	7.18
LP(2)O2	BD*(1)Si1-C20	3.2
LP(2)O2	BD*(1)Si3-C8	7.38
LP(2)O2	BD*(1)Si3-C23	2.37
LP(1)N38	BD*(1)C35-H36	6.91
BD(1)Si1-C12	BD*(1)Si1-O2	2.71
LP(1)N41	BD*(1)C29-H31	6.9
BD(1)Si1-C20	BD*(1)Si1-O2	3.34
BD(1)Si1-C20	BD*(1)C32-C35	3.48
BD(1)O2-Si3	RY*(7)Si1	1.76
BD(1)O2-Si3	BD*(1)Si1-O2	1.84
BD(1)Si1-O2	BD*(1)O2-Si3	1.83
BD(1)Si3-C8	BD*(1)O2-Si3	2.67
BD(1)Si3-C16	BD*(1)O2-Si3	2.29
BD(1)Si3-C23	BD*(1)O2-Si3	3.37
BD(1)Si3-C23	BD*(1)C26-C29	3.46
BD(1)C4-H5	BD*(1)Si1-O2	1.78
BD(1)C8-H11	BD*(1)O2-Si3	1.84

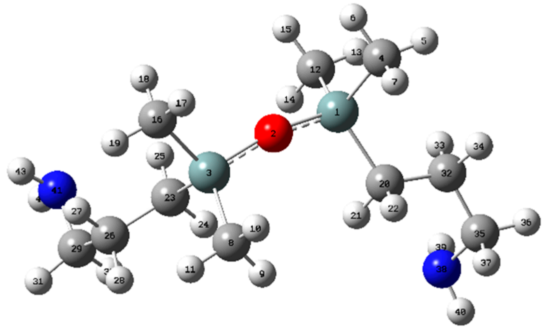

Figure S6. APTMDS – Atoms labels

Table S14. Wiberg bond order for ClMTMDS

Vacuum		Water		Methanol	
**Bond**	**bond order**	**Bond**	**bond order**	**Bond**	**bond order**
1(Si)-2(O)	1.1400146	1(Si)-2(O)	1.13655584	1(Si)-2(O)	1.13748427
1(Si)-12(C)	0.95830373	1(Si)-12(C)	0.95881853	1(Si)-12(C)	0.95886238
1(Si)-16(C)	0.96402099	1(Si)-16(C)	0.96371834	1(Si)-16(C)	0.96359277
1(Si)-20(C)	0.90778637	1(Si)-20(C)	0.90788235	1(Si)-20(C)	0.90777761
1(Si)-21(H)	0.05179981	1(Si)-21(H)	0.05143908	1(Si)-21(H)	0.05140356

1(Si)-27(Cl)	0.09508472	1(Si)-27(Cl)	0.09144385	1(Si)-27(Cl)	0.09156713
2(O)-3(Si)	1.14002035	2(O)-3(Si)	1.1365708	2(O)-3(Si)	1.13748612
3(Si)-4(C)	0.96402108	3(Si)-4(C)	0.96371619	3(Si)-4(C)	0.96359574
3(Si)-8(C)	0.95830266	3(Si)-8(C)	0.95881143	3(Si)-8(C)	0.95885699
3(Si)-23(C)	0.90778595	3(Si)-23(C)	0.90788642	3(Si)-23(C)	0.90777981
3(Si)-26(Cl)	0.09508269	3(Si)-26(Cl)	0.09145242	3(Si)-26(Cl)	0.09156828
4(C)-5(H)	0.95861945	4(C)-5(H)	0.95940102	4(C)-5(H)	0.95958214
4(C)-6(H)	0.95852747	4(C)-6(H)	0.95854177	4(C)-6(H)	0.95866943
4(C)-7(H)	0.95875291	4(C)-7(H)	0.96044973	4(C)-7(H)	0.96042939
8(C)-9(H)	0.96169327	8(C)-9(H)	0.9609803	8(C)-9(H)	0.9610274
8(C)-10(H)	0.96125819	8(C)-10(H)	0.95932484	8(C)-10(H)	0.95946696
8(C)-11(H)	0.95875557	8(C)-11(H)	0.95904605	8(C)-11(H)	0.95908321
12(C)-13(H)	0.95875556	12(C)-13(H)	0.95904956	12(C)-13(H)	0.95908495
12(C)-14(H)	0.96169272	12(C)-14(H)	0.96097949	12(C)-14(H)	0.96102772
12(C)-15(H)	0.96125847	12(C)-15(H)	0.95932922	12(C)-15(H)	0.95946907
16(C)-17(H)	0.95875203	16(C)-17(H)	0.96044477	16(C)-17(H)	0.96042855
16(C)-18(H)	0.95861971	16(C)-18(H)	0.95939593	16(C)-18(H)	0.95958133
16(C)-19(H)	0.95852747	16(C)-19(H)	0.95853998	16(C)-19(H)	0.95866717
20(C)-21(H)	0.96372651	20(C)-21(H)	0.96347943	20(C)-21(H)	0.96354921
20(C)-22(H)	0.96374451	20(C)-22(H)	0.96391818	20(C)-22(H)	0.96389523
20(C)-27(Cl)	1.212278	20(C)-27(Cl)	1.19722847	20(C)-27(Cl)	1.19767786
23(C)-24(H)	0.96372636	23(C)-24(H)	0.96348061	23(C)-24(H)	0.963549
23(C)-25(H)	0.96374434	23(C)-25(H)	0.96392041	23(C)-25(H)	0.96389584
23(C)-26(Cl)	1.2122759	23(C)-26(Cl)	1.19722131	23(C)-26(Cl)	1.19767833

Table S15. Second Order Perturbation Theory Analysis of Fock Matrix in NBO Basis for:

ClMTMDS – vacuum

Donor NBO(i)	Acceptor NBO(j)	E(2)
BD(1)C16-H18	BD*(1)Si1-C20	1.67
BD(1)C16-H19	BD*(1)Si1-O2	2.13
CR(2)Si1	BD*(1)Si1-O2	2.5
CR(2)Si1	BD*(1)O2-Si3	2.4
CR(2)Si3	BD*(1)Si1-O2	2.4
CR(2)Si3	BD*(1)O2-Si3	2.5
CR(1)C20	BD*(1)C20-Cl27	2.74
CR(1)C23	BD*(1)C23-Cl26	2.74
LP(1)O2	RY*(2)Si1	2.35
LP(1)O2	RY*(2)Si3	2.35
LP(1)O2	BD*(1)Si1-C12	3.33
LP(1)O2	BD*(1)Si1-C16	6.95
LP(1)O2	BD*(1)Si3-C4	6.95
LP(1)O2	BD*(1)Si3-C8	3.33
LP(2)O2	RY*(1)Si1	2
LP(2)O2	RY*(1)Si3	2
LP(2)O2	BD*(1)Si1-C12	3.16
LP(2)O2	BD*(1)Si1-C20	7.63
LP(2)O2	BD*(1)Si3-C8	3.16
LP(2)O2	BD*(1)Si3-C23	7.63
LP(2)Cl26	BD*(1)Si3-C23	1.95
LP(2)Cl26	BD*(1)C23-H25	1.57
LP(3)Cl26	BD*(1)C23-H24	3.52
LP(3)Cl26	BD*(1)C23-H25	2.75
LP(2)Cl27	BD*(1)Si1-C20	1.95
BD(1)Si1-C12	BD*(1)C20-Cl27	1.86
LP(2)Cl27	BD*(1)C20-H22	1.57
LP(3)Cl27	BD*(1)C20-H21	3.52
LP(3)Cl27	BD*(1)C20-H22	2.75
BD(1)Si1-O2	BD*(1)O2-Si3	2.02
BD(1)Si1-C16	BD*(1)Si1-O2	3.06
BD(1)Si1-C20	BD*(1)Si1-O2	2.66

BD(1)Si1-C20	BD*(1)Si1-C12	1.5
BD(1)O2-Si3	BD*(1)Si1-O2	2.02
BD(1)Si3-C4	BD*(1)O2-Si3	3.06
BD(1)Si3-C8	BD*(1)O2-Si3	2.42
BD(1)Si3-C8	BD*(1)C23-Cl26	1.86
BD(1)Si3-C23	BD*(1)O2-Si3	2.66
BD(1)Si3-C23	BD*(1)Si3-C8	1.5
BD(1)Si1-C12	BD*(1)Si1-O2	2.42
BD(1)C4-H5	BD*(1)Si3-C23	1.67
BD(1)C4-H6	BD*(1)O2-Si3	2.13
BD(1)C4-H7	BD*(1)Si3-C8	1.57
BD(1)C8-H10	BD*(1)O2-Si3	1.89
BD(1)C12-H15	BD*(1)Si1-O2	1.89
BD(1)C16-H17	BD*(1)Si1-C12	1.57

ClMTMDS – water

Donor NBO(i)	Acceptor NBO(j)	E(2)
BD(1)C8-H10	BD*(1)O2-Si3	2.03
BD(1)C12-H15	BD*(1)Si1-O2	2.02
BD(1)C16-H17	BD*(1)Si1-C12	1.52
BD(1)C16-H18	BD*(1)Si1-C20	1.67
BD(1)C16-H19	BD*(1)Si1-O2	2.09
BD(1)Si1-C12	BD*(1)Si1-O2	2.77
CR(2)Si1	BD*(1)Si1-O2	2.52
CR(2)Si1	BD*(1)O2-Si3	2.33
CR(2)Si3	BD*(1)Si1-O2	2.33
CR(2)Si3	BD*(1)O2-Si3	2.52
CR(1)C20	BD*(1)C20-Cl27	2.86
CR(1)C23	BD*(1)C23-Cl26	2.86
LP(1)O2	BD*(1)Si1-C16	3.44
LP(1)O2	BD*(1)Si1-C20	6.29
LP(1)O2	BD*(1)Si3-C4	3.2
LP(1)O2	BD*(1)Si3-C23	6.38
LP(2)O2	RY*(2)Si1	1.52
LP(2)O2	RY*(2)Si3	1.6
LP(2)O2	BD*(1)Si1-C12	6.97
LP(2)O2	BD*(1)Si1-C16	4.04
LP(2)O2	BD*(1)Si3-C4	4.29
LP(2)O2	BD*(1)Si3-C8	6.83
LP(2)Cl26	BD*(1)Si3-C23	1.97
LP(3)Cl26	BD*(1)C23-H24	2.75
LP(3)Cl26	BD*(1)C23-H25	3.08
LP(2)Cl27	BD*(1)Si1-C20	1.97
LP(3)Cl27	BD*(1)C20-H21	2.75
LP(3)Cl27	BD*(1)C20-H22	3.08
BD(1)Si1-C12	BD*(1)C20-Cl27	1.99
BD(1)Si1-C16	BD*(1)Si1-O2	3.07
BD(1)Si1-C20	BD*(1)Si1-O2	2.37
BD(1)O2-Si3	BD*(1)Si1-O2	1.92
BD(1)Si1-O2	BD*(1)O2-Si3	1.92
BD(1)Si3-C4	BD*(1)O2-Si3	3.07
BD(1)Si3-C8	BD*(1)O2-Si3	2.77
BD(1)Si3-C8	BD*(1)C23-Cl26	1.99
BD(1)Si3-C23	BD*(1)O2-Si3	2.37
BD(1)C4-H5	BD*(1)Si3-C23	1.67
BD(1)C4-H6	BD*(1)O2-Si3	2.09
BD(1)C4-H7	BD*(1)Si3-C8	1.52

ClMTMDS - methanol

Donor NBO(i)	Acceptor NBO(j)	E(2)
BD(1)C8-H10	BD*(1)O2-Si3	2.02
BD(1)C12-H15	BD*(1)Si1-O2	2.02
BD(1)C16-H17	BD*(1)Si1-C12	1.51
BD(1)C16-H18	BD*(1)Si1-C20	1.66
BD(1)C16-H19	BD*(1)Si1-O2	2.08
BD(1)Si1-C12	BD*(1)Si1-O2	2.74
CR(2)Si1	BD*(1)Si1-O2	2.51
CR(2)Si1	BD*(1)O2-Si3	2.36
CR(2)Si3	BD*(1)Si1-O2	2.36
CR(2)Si3	BD*(1)O2-Si3	2.51
CR(1)C20	BD*(1)C20-Cl27	2.86
CR(1)C23	BD*(1)C23-Cl26	2.86
LP(1)O2	BD*(1)Si1-C16	3.75
LP(1)O2	BD*(1)Si1-C20	6.33
LP(1)O2	BD*(1)Si3-C4	3.42
LP(1)O2	BD*(1)Si3-C23	6.48
LP(2)O2	RY*(2)Si1	1.59
LP(2)O2	RY*(2)Si3	1.69
LP(2)O2	BD*(1)Si1-C12	7.11
LP(2)O2	BD*(1)Si1-C16	3.74
LP(2)O2	BD*(1)Si3-C4	4.08
LP(2)O2	BD*(1)Si3-C8	6.93
LP(2)Cl26	BD*(1)Si3-C23	1.98
LP(3)Cl26	BD*(1)C23-H24	2.81
LP(3)Cl26	BD*(1)C23-H25	3.04
LP(2)Cl27	BD*(1)Si1-C20	1.98
LP(3)Cl27	BD*(1)C20-H21	2.81
LP(3)Cl27	BD*(1)C20-H22	3.04
BD(1)Si1-C12	BD*(1)C20-Cl27	1.98
BD(1)Si1-C16	BD*(1)Si1-O2	3.04
BD(1)Si1-C20	BD*(1)Si1-O2	2.39
BD(1)O2-Si3	BD*(1)Si1-O2	1.94
BD(1)Si1-O2	BD*(1)O2-Si3	1.94
BD(1)Si3-C4	BD*(1)O2-Si3	3.04

BD(1)Si3-C8	BD*(1)O2-Si3	2.74
BD(1)Si3-C8	BD*(1)C23-Cl26	1.98
BD(1)Si3-C23	BD*(1)O2-Si3	2.39

BD(1)C4-H5	BD*(1)Si3-C23	1.66
BD(1)C4-H6	BD*(1)O2-Si3	2.08
BD(1)C4-H7	BD*(1)Si3-C8	1.51

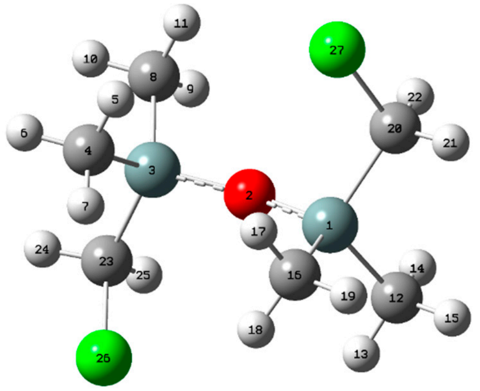

Figure S7. ClMTMDS – atoms labels

Table S16. Wiberg bond order for Silanol

Vacuum		Water		Methanol	
**Bond**	**bond order**	**Bond**	**bond order**	**Bond**	**bond order**
1(Si)-2(O)	1.16542152	1(Si)-2(O)	1.15468383	1(Si)-2(O)	1.15491916
1(Si)-3(C)	0.95443132	1(Si)-3(C)	0.95668923	1(Si)-3(C)	0.95664315
1(Si)-7(C)	0.95377317	1(Si)-7(C)	0.95471471	1(Si)-7(C)	0.95466418
1(Si)-11(C)	0.91665407	1(Si)-11(C)	0.91945161	1(Si)-11(C)	0.91938253
1(Si)-13(H)	0.05691297	1(Si)-13(H)	0.05695471	1(Si)-13(H)	0.05695933
1(Si)-14(N)	0.07577149	1(Si)-14(N)	0.07643425	1(Si)-14(N)	0.07639908
1(Si)-25(H)	0.06426339	1(Si)-25(H)	0.06302769	1(Si)-25(H)	0.06307128
2(O)-25(H)	1.18337529	2(O)-25(H)	1.1762816	2(O)-25(H)	1.17653584
3(C)-4(H)	0.96352698	3(C)-4(H)	0.96333756	3(C)-4(H)	0.96332925
3(C)-5(H)	0.95915636	3(C)-5(H)	0.95797135	3(C)-5(H)	0.95801613
3(C)-6(H)	0.95937338	3(C)-6(H)	0.95927442	3(C)-6(H)	0.95926615
7(C)-8(H)	0.96448739	7(C)-8(H)	0.96304921	7(C)-8(H)	0.96313301
7(C)-9(H)	0.96336984	7(C)-9(H)	0.96347758	7(C)-9(H)	0.9634713
7(C)-10(H)	0.9584978	7(C)-10(H)	0.9599632	7(C)-10(H)	0.9598965
11(C)-12(H)	0.93823999	11(C)-12(H)	0.93780518	11(C)-12(H)	0.93782626
11(C)-13(H)	0.94335095	11(C)-13(H)	0.94286301	11(C)-13(H)	0.94287588
11(C)-14(N)	1.11323591	11(C)-14(N)	1.11337591	11(C)-14(N)	1.11337164
14(N)-15(H)	1.02351643	14(N)-15(H)	1.02179386	14(N)-15(H)	1.02185857
14(N)-16(C)	1.12178356	14(N)-16(C)	1.11886063	14(N)-16(C)	1.11896822
16(C)-17(H)	0.92358195	16(C)-17(H)	0.92487599	16(C)-17(H)	0.92481042
16(C)-18(H)	0.92200013	16(C)-18(H)	0.92218378	16(C)-18(H)	0.92218837
16(C)-19(C)	1.03439816	16(C)-19(C)	1.03613762	16(C)-19(C)	1.03607642
19(C)-20(H)	0.93192588	19(C)-20(H)	0.93198719	19(C)-20(H)	0.9320026
19(C)-21(H)	0.93504953	19(C)-21(H)	0.93628089	19(C)-21(H)	0.93622903
19(C)-22(N)	1.14129787	19(C)-22(N)	1.13656204	19(C)-22(N)	1.13671316
22(N)-23(H)	1.04463288	22(N)-23(H)	1.04230904	22(N)-23(H)	1.04238023

22(N)-24(H)	1.04631796	22(N)-24(H)	1.04418308	22(N)-24(H)	1.04426126

Table S17. Second Order Perturbation Theory Analysis of Fock Matrix in NBO Basis for:

Silanol – vacuum

Donor NBO(i)	Acceptor NBO(j)	E(2)
BD(1)Si1-C3	BD*(1)Si1-C7	1.47
LP(1)O2	RY*(2)Si1	2.46
LP(1)O2	RY*(1)H25	1.85
LP(1)O2	BD*(1)Si1-C3	1.62
BD(1)Si1-C3	BD*(1)Si1-C11	1.31
LP(1)O2	BD*(1)Si1-C11	3.34
LP(2)O2	RY*(1)Si1	3.34
LP(2)O2	RY*(2)H25	1.95
LP(2)O2	BD*(1)Si1-C3	5.85
LP(2)O2	BD*(1)Si1-C7	6.76
LP(1)N14	RY*(2)H15	1.48
LP(1)N14	BD*(1)C11-H12	6.41
LP(1)N14	BD*(1)C16-H17	1.43
LP(1)N14	BD*(1)C16-H18	8.15
LP(1)N22	RY*(1)H23	1.37
LP(1)N22	BD*(1)C19-H20	7.89
BD(1)Si1-C7	BD*(1)Si1-O2	3.04
BD(1)Si1-C7	BD*(1)Si1-C3	1.46
BD(1)Si1-C11	RY*(1)N14	1.82
BD(1)Si1-C11	BD*(1)Si1-O2	2.24
BD(1)Si1-C11	BD*(1)N14-H15	2.19
BD(1)O2-H25	RY*(3)Si1	2.39
BD(1)C3-H5	BD*(1)Si1-C11	1.53
BD(1)C3-H6	BD*(1)Si1-O2	1.83
BD(1)C7-H8	BD*(1)Si1-C11	1.31
BD(1)C7-H10	BD*(1)Si1-O2	2.06
BD(1)C11-H13	BD*(1)Si1-C3	1.7
BD(1)Si1-O2	BD*(1)Si1-C11	1.61
BD(1)C11-H13	BD*(1)N14-C16	3.34
BD(1)C11-N14	BD*(1)C16-C19	1.84
BD(1)N14-H15	BD*(1)Si1-C11	1.82
BD(1)N14-H15	BD*(1)C16-H17	2.45
BD(1)N14-C16	RY*(1)C11	1.4
BD(1)N14-C16	BD*(1)C19-N22	1.58
BD(1)C16-H17	BD*(1)N14-H15	3.03
BD(1)C16-H17	BD*(1)C19-H20	2.69
BD(1)C16-H18	BD*(1)C19-H21	2.46
BD(1)C16-C19	BD*(1)C11-N14	3.19
BD(1)C16-C19	BD*(1)N22-H23	1.81
BD(1)C19-H20	BD*(1)C16-H17	2.51
BD(1)C19-H21	BD*(1)C16-H18	2.88
BD(1)C19-H21	BD*(1)N22-H24	2.99
BD(1)C19-N22	BD*(1)N14-C16	1.91
BD(1)N22-H23	BD*(1)C16-C19	2.62
BD(1)N22-H24	BD*(1)C19-H21	2.12
CR(2)Si1	BD*(1)Si1-O2	2.71
BD(1)Si1-C3	BD*(1)Si1-O2	3.19

Silanol - water

Donor NBO(i)	Acceptor NBO(j)	E(2)
BD(1)C7-H9	BD*(1)Si1-C3	1.28
BD(1)Si1-C3	BD*(1)Si1-C7	1.45
LP(1)O2	RY*(2)Si1	2.68
LP(1)O2	RY*(1)H25	1.65
LP(1)O2	BD*(1)Si1-C3	1.49
LP(1)O2	BD*(1)Si1-C11	3.03
BD(1)Si1-C3	BD*(1)Si1-C11	1.33
LP(2)O2	RY*(1)Si1	3.28
LP(2)O2	RY*(2)H25	1.7
LP(2)O2	BD*(1)Si1-C3	5.76
LP(2)O2	BD*(1)Si1-C7	6.55
LP(1)N14	RY*(2)H15	1.37
LP(1)N14	BD*(1)C11-H12	6.43
LP(1)N14	BD*(1)C16-H17	1.37
LP(1)N14	BD*(1)C16-H18	7.72
LP(1)N22	BD*(1)C19-H20	7.23
BD(1)Si1-C7	BD*(1)Si1-O2	3.01
BD(1)Si1-C7	BD*(1)Si1-C3	1.43
BD(1)Si1-C11	RY*(1)N14	1.73
BD(1)Si1-C11	BD*(1)Si1-O2	2.49
BD(1)Si1-C11	BD*(1)N14-H15	2.12
BD(1)O2-H25	RY*(3)Si1	2.27
BD(1)C3-H5	BD*(1)Si1-C11	1.59
BD(1)C3-H6	BD*(1)Si1-O2	1.86
BD(1)C7-H8	BD*(1)Si1-C11	1.38
BD(1)C7-H10	BD*(1)Si1-O2	2.03
BD(1)Si1-O2	BD*(1)Si1-C11	1.62
BD(1)C11-H13	BD*(1)Si1-C3	1.71
BD(1)C11-H13	BD*(1)N14-C16	3.34
BD(1)C11-N14	BD*(1)C16-C19	1.85
BD(1)N14-H15	BD*(1)Si1-C11	1.93

BD(1)N14-H15	BD*(1)C16-H17	2.51
BD(1)N14-C16	RY*(1)C11	1.37
BD(1)N14-C16	BD*(1)C19-N22	1.6
BD(1)C16-H17	BD*(1)N14-H15	2.86
BD(1)C16-H17	BD*(1)C19-H20	2.6
BD(1)C16-H18	BD*(1)C19-H21	2.49
BD(1)C16-C19	BD*(1)C11-N14	3.13
BD(1)C16-C19	BD*(1)N22-H23	1.66
BD(1)C19-H20	BD*(1)C16-H17	2.58
BD(1)C19-H21	BD*(1)C16-H18	2.83
BD(1)C19-H21	BD*(1)N22-H24	2.77
BD(1)C19-N22	BD*(1)N14-C16	1.96
BD(1)N22-H23	BD*(1)C16-C19	2.69
BD(1)N22-H24	BD*(1)C19-H21	2.18
CR(2)Si1	BD*(1)Si1-O2	2.75
BD(1)Si1-C3	BD*(1)Si1-O2	3.13

Silanol - methanol

Donor NBO(i)	Acceptor NBO(j)	E(2)
BD(1)C7-H9	BD*(1)Si1-C3	1.28
BD(1)Si1-C3	BD*(1)Si1-C7	1.45
LP(1)O2	RY*(2)Si1	2.67
LP(1)O2	RY*(1)H25	1.65
LP(1)O2	BD*(1)Si1-C3	1.5
BD(1)Si1-C3	BD*(1)Si1-C11	1.33
LP(1)O2	BD*(1)Si1-C11	3.04
LP(2)O2	RY*(1)Si1	3.28
LP(2)O2	RY*(2)H25	1.71
LP(2)O2	BD*(1)Si1-C3	5.74
LP(2)O2	BD*(1)Si1-C7	6.56
LP(1)N14	RY*(2)H15	1.38
LP(1)N14	BD*(1)C11-H12	6.43
LP(1)N14	BD*(1)C16-H17	1.37
LP(1)N14	BD*(1)C16-H18	7.73
LP(1)N22	BD*(1)C19-H20	7.25
BD(1)Si1-C7	BD*(1)Si1-O2	3.01
BD(1)Si1-C7	BD*(1)Si1-C3	1.43
BD(1)Si1-C11	RY*(1)N14	1.73
BD(1)Si1-C11	BD*(1)Si1-O2	2.48
BD(1)Si1-C11	BD*(1)N14-H15	2.12
BD(1)O2-H25	RY*(3)Si1	2.27
BD(1)C3-H5	BD*(1)Si1-C11	1.59
BD(1)C3-H6	BD*(1)Si1-O2	1.86
BD(1)C7-H8	BD*(1)Si1-C11	1.38
BD(1)C7-H10	BD*(1)Si1-O2	2.03
BD(1)Si1-O2	BD*(1)Si1-C11	1.62
BD(1)C11-H13	BD*(1)Si1-C3	1.71
BD(1)C11-H13	BD*(1)N14-C16	3.34
BD(1)C11-N14	BD*(1)C16-C19	1.85
BD(1)N14-H15	BD*(1)Si1-C11	1.93
BD(1)N14-H15	BD*(1)C16-H17	2.51
BD(1)N14-C16	RY*(1)C11	1.37
BD(1)N14-C16	BD*(1)C19-N22	1.6
BD(1)C16-H17	BD*(1)N14-H15	2.86
BD(1)C16-H17	BD*(1)C19-H20	2.61
BD(1)C16-H18	BD*(1)C19-H21	2.49
BD(1)C16-C19	BD*(1)C11-N14	3.13
BD(1)C16-C19	BD*(1)N22-H23	1.66
BD(1)C19-H20	BD*(1)C16-H17	2.58
BD(1)C19-H21	BD*(1)C16-H18	2.83
BD(1)C19-H21	BD*(1)N22-H24	2.78
BD(1)C19-N22	BD*(1)N14-C16	1.96
BD(1)N22-H23	BD*(1)C16-C19	2.69
BD(1)N22-H24	BD*(1)C19-H21	2.18
CR(2)Si1	BD*(1)Si1-O2	2.75
BD(1)Si1-C3	BD*(1)Si1-O2	3.13

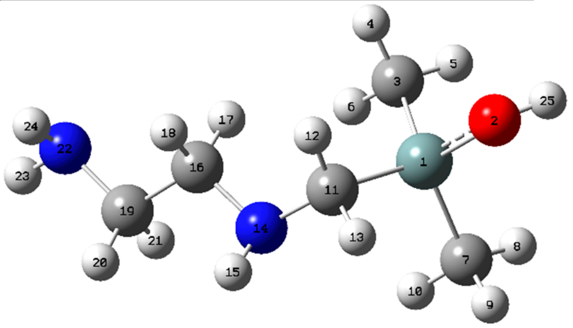

Figure S8. Silanol – atoms labels

Table S18. Wiberg bond order for H-Silanol

Vacuum	Water	Methanol

Bond	bond order	Bond	bond order	Bond	bond order
1(Si)-2(O)	1.22197541	1(Si)-2(O)	1.18001643	1(Si)-2(O)	1.181631
1(Si)-3(C)	0.98428817	1(Si)-3(C)	0.97751099	1(Si)-3(C)	0.97795759
1(Si)-7(C)	0.96628469	1(Si)-7(C)	0.97254643	1(Si)-7(C)	0.97238366
1(Si)-11(C)	0.85265559	1(Si)-11(C)	0.86848576	1(Si)-11(C)	0.8675374
1(Si)-25(H)	0.06312142	1(Si)-25(H)	0.06328369	1(Si)-25(H)	0.06334039
2(O)-25(H)	1.15962802	2(O)-25(H)	1.16625792	2(O)-25(H)	1.16621663
3(C)-4(H)	0.95581243	3(C)-4(H)	0.95842974	3(C)-4(H)	0.95829358
3(C)-5(H)	0.95040526	3(C)-5(H)	0.95465869	3(C)-5(H)	0.95436943
3(C)-6(H)	0.95327891	3(C)-6(H)	0.95606049	3(C)-6(H)	0.95595071
7(C)-8(H)	0.94745229	7(C)-8(H)	0.95519654	7(C)-8(H)	0.95501097
7(C)-9(H)	0.95839002	7(C)-9(H)	0.95873793	7(C)-9(H)	0.95880014
7(C)-10(H)	0.95779494	7(C)-10(H)	0.95644976	7(C)-10(H)	0.95644059
11(C)-12(H)	0.94940658	11(C)-12(H)	0.94619146	11(C)-12(H)	0.94633902
11(C)-13(H)	0.94256187	11(C)-13(H)	0.94131798	11(C)-13(H)	0.94151697
11(C)-14(N)	1.00274458	11(C)-14(N)	1.00540466	11(C)-14(N)	1.00517199
14(N)-15(H)	0.94635423	14(N)-15(H)	0.93845831	14(N)-15(H)	0.93878058
14(N)-16(C)	0.98531098	14(N)-16(C)	0.99253276	14(N)-16(C)	0.99227986
14(N)-26(H)	0.94555165	14(N)-26(H)	0.94204714	14(N)-26(H)	0.94222407
16(C)-17(H)	0.92374402	16(C)-17(H)	0.92443926	16(C)-17(H)	0.9243407
16(C)-18(H)	0.92873112	16(C)-18(H)	0.92813208	16(C)-18(H)	0.92819867
16(C)-19(C)	1.03234306	16(C)-19(C)	1.03574828	16(C)-19(C)	1.03561754
19(C)-20(H)	0.92872738	19(C)-20(H)	0.92985573	19(C)-20(H)	0.92986323
19(C)-21(H)	0.93249322	19(C)-21(H)	0.93253662	19(C)-21(H)	0.93250682
19(C)-22(N)	1.15735513	19(C)-22(N)	1.14313727	19(C)-22(N)	1.14368423
22(N)-23(H)	1.03324487	22(N)-23(H)	1.03661627	22(N)-23(H)	1.03647469
22(N)-24(H)	1.03836274	22(N)-24(H)	1.04032272	22(N)-24(H)	1.04028864

Table S19. Second Order Perturbation Theory Analysis of Fock Matrix in NBO Basis for:

H-silanol - vacuum

Donor NBO(i)	Acceptor NBO(j)	E(2)
BD(1)N14-H26	BD*(1)C11-H12	1.32
BD(1)N14-H26	BD*(1)C16-H18	1.39
BD(1)Si1-C3	BD*(1)Si1-O2	3.16
LP(1)O2	RY*(2)Si1	2.75
LP(1)O2	RY*(1)H25	1.93
LP(1)O2	BD*(1)Si1-C3	3.88
LP(1)O2	BD*(1)Si1-C11	3.88
LP(2)O2	RY*(1)Si1	3.89
BD(1)Si1-C3	BD*(1)Si1-C11	1.59
LP(2)O2	RY*(2)H25	1.68
LP(2)O2	BD*(1)Si1-C3	3.35
LP(2)O2	BD*(1)Si1-C7	9.25
LP(2)O2	BD*(1)Si1-C11	1.42
LP(1)N22	RY*(1)H23	1.43
LP(1)N22	BD*(1)N14-C16	1.7
LP(1)N22	BD*(1)C16-C19	3.1
LP(1)N22	BD*(1)C19-H20	7.11
BD(1)Si1-C7	BD*(1)Si1-O2	2.3
BD(1)Si1-C7	BD*(1)Si1-C11	2.08
BD(1)Si1-C11	BD*(1)Si1-O2	2.06
BD(1)Si1-C11	BD*(1)Si1-C7	1.58
BD(1)Si1-C11	BD*(1)N14-H15	2.26
BD(1)O2-H25	RY*(3)Si1	2.34
BD(1)C3-H4	BD*(1)Si1-C7	1.64
BD(1)C3-H5	BD*(1)Si1-C11	2.02
BD(1)C3-H6	BD*(1)Si1-O2	2.18
BD(1)C7-H8	BD*(1)Si1-C11	1.71
BD(1)C7-H9	BD*(1)Si1-C3	1.41
BD(1)C7-H10	BD*(1)Si1-O2	1.99

BD(1)C11-H12	BD*(1)N14-H26	2.38
BD(1)Si1-O2	BD*(1)Si1-C11	1.55
BD(1)C11-H13	BD*(1)N14-C16	3.63
BD(1)N14-H15	BD*(1)Si1-C11	1.4
BD(1)N14-H15	BD*(1)C16-H17	1.44
BD(1)C16-H17	BD*(1)N14-H15	2.3
BD(1)C16-H17	BD*(1)C19-H20	2.26
BD(1)C16-H18	BD*(1)N14-H26	2.41
BD(1)C16-H18	BD*(1)C19-H21	2.17
BD(1)C16-C19	RY*(1)N22	1.45
BD(1)C16-C19	BD*(1)C11-N14	2.76
BD(1)C16-C19	BD*(1)N22-H23	1.5
BD(1)C19-H20	BD*(1)C16-H17	2.56
BD(1)C19-H21	BD*(1)C16-H18	2.77
BD(1)C19-H21	BD*(1)N22-H24	2.45
BD(1)C19-N22	BD*(1)N14-C16	2.99
BD(1)N22-H23	BD*(1)C16-C19	2.2
BD(1)N22-H24	RY*(2)C19	1.64
BD(1)N22-H24	BD*(1)C19-H21	1.84
CR(2)Si1	BD*(1)Si1-O2	2.5

H-silanol - water

Donor NBO(i)	Acceptor NBO(j)	E(2)
BD(1)Si1-C3	BD*(1)Si1-C11	1.59
LP(1)O2	RY*(2)Si1	2.97
LP(1)O2	RY*(1)H25	1.74
LP(1)O2	BD*(1)Si1-C3	2.77
LP(1)O2	BD*(1)Si1-C7	2.45
LP(2)O2	RY*(1)Si1	3.31
LP(2)O2	RY*(2)H25	1.65
LP(2)O2	BD*(1)Si1-C7	4.69
LP(2)O2	BD*(1)Si1-C11	7.91
LP(1)N22	BD*(1)C16-C19	1.79
LP(1)N22	BD*(1)C19-H20	6.94
BD(1)Si1-C7	BD*(1)Si1-O2	2.79
BD(1)Si1-C7	BD*(1)Si1-C11	1.97
BD(1)Si1-C11	BD*(1)Si1-O2	3.38
BD(1)Si1-C11	BD*(1)Si1-C7	1.56
BD(1)Si1-C11	BD*(1)N14-H15	1.95
BD(1)O2-H25	RY*(3)Si1	2.3
BD(1)C3-H4	BD*(1)Si1-C7	1.62
BD(1)C3-H5	BD*(1)Si1-C11	1.87
BD(1)C3-H6	BD*(1)Si1-O2	1.66
BD(1)C7-H8	BD*(1)Si1-C11	1.71
BD(1)C7-H9	BD*(1)Si1-C3	1.53
BD(1)C7-H10	BD*(1)Si1-O2	2.13
BD(1)C11-H12	BD*(1)N14-H26	2.34

BD(1)C11-H13	BD*(1)N14-C16	3.65
BD(1)C11-N14	BD*(1)C16-C19	1.31
BD(1)N14-H15	BD*(1)Si1-C11	1.67
BD(1)N14-H15	BD*(1)C16-H17	1.54
BD(1)N14-H26	BD*(1)C11-H12	1.36
BD(1)N14-H26	BD*(1)C16-H18	1.41
BD(1)C16-H17	BD*(1)N14-H15	2.18
BD(1)C16-H17	BD*(1)C19-H20	2.26
BD(1)C16-H18	BD*(1)N14-H26	2.39
BD(1)C16-H18	BD*(1)C19-H21	2.19
BD(1)C16-C19	BD*(1)C11-N14	2.84
BD(1)C16-C19	BD*(1)N22-H23	1.59
BD(1)C19-H20	BD*(1)C16-H17	2.55
BD(1)C19-H21	BD*(1)C16-H18	2.73
BD(1)C19-H21	BD*(1)N22-H24	2.56
BD(1)C19-N22	BD*(1)N14-C16	2.83
BD(1)N22-H23	BD*(1)C16-C19	2.6
BD(1)Si1-C3	BD*(1)Si1-O2	1.96
BD(1)N22-H24	RY*(2)C19	1.75
BD(1)N22-H24	BD*(1)C19-H21	1.98
CR(2)Si1	BD*(1)Si1-O2	2.46

H-silanol - methanol

Donor NBO(i)	Acceptor NBO(j)	E(2)
BD(1)Si1-C3	BD*(1)Si1-C11	1.6
LP(1)O2	RY*(2)Si1	2.97
LP(1)O2	RY*(1)H25	1.74
LP(1)O2	BD*(1)Si1-C3	2.77
LP(1)O2	BD*(1)Si1-C7	2.52
LP(2)O2	RY*(1)Si1	3.32
LP(2)O2	RY*(2)H25	1.65
LP(2)O2	BD*(1)Si1-C7	4.6
LP(2)O2	BD*(1)Si1-C11	8.01
LP(1)N22	BD*(1)C16-C19	1.83
LP(1)N22	BD*(1)C19-H20	6.97
BD(1)Si1-C7	BD*(1)Si1-O2	2.79
BD(1)Si1-C7	BD*(1)Si1-C11	1.98
BD(1)Si1-C11	BD*(1)Si1-O2	3.37
BD(1)Si1-C11	BD*(1)Si1-C7	1.56
BD(1)Si1-C11	BD*(1)N14-H15	1.96
BD(1)O2-H25	RY*(3)Si1	2.31
BD(1)C3-H4	BD*(1)Si1-C7	1.62
BD(1)C3-H5	BD*(1)Si1-C11	1.89
BD(1)C3-H6	BD*(1)Si1-O2	1.66
BD(1)C7-H8	BD*(1)Si1-C11	1.7
BD(1)C7-H9	BD*(1)Si1-C3	1.52
BD(1)C7-H10	BD*(1)Si1-O2	2.14

BD(1)C11-H12	BD*(1)N14-H26	2.34
BD(1)C11-H13	BD*(1)N14-C16	3.65
BD(1)C11-N14	BD*(1)C16-C19	1.3
BD(1)N14-H15	BD*(1)Si1-C11	1.65
BD(1)N14-H15	BD*(1)C16-H17	1.54
BD(1)N14-H26	BD*(1)C11-H12	1.35
BD(1)N14-H26	BD*(1)C16-H18	1.41
BD(1)C16-H17	BD*(1)N14-H15	2.19
BD(1)C16-H17	BD*(1)C19-H20	2.26
BD(1)C16-H18	BD*(1)N14-H26	2.39
BD(1)C16-H18	BD*(1)C19-H21	2.19

BD(1)C16-C19	BD*(1)C11-N14	2.84
BD(1)C16-C19	BD*(1)N22-H23	1.59
BD(1)C19-H20	BD*(1)C16-H17	2.54
BD(1)C19-H21	BD*(1)C16-H18	2.73
BD(1)C19-H21	BD*(1)N22-H24	2.56
BD(1)C19-N22	BD*(1)N14-C16	2.84
BD(1)Si1-C3	BD*(1)Si1-O2	1.94
BD(1)N22-H23	BD*(1)C16-C19	2.59
BD(1)N22-H24	RY*(2)C19	1.76
BD(1)N22-H24	BD*(1)C19-H21	1.98
CR(2)Si1	BD*(1)Si1-O2	2.46

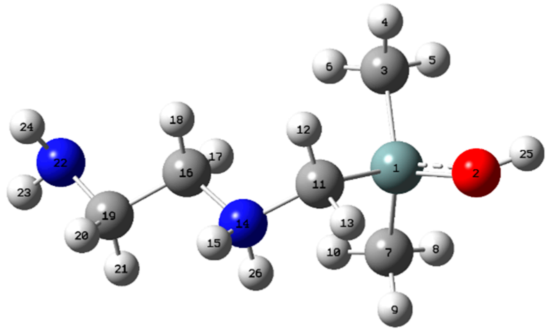

Figure S9. H-silanol – atoms labels

Table S20. Wiberg bond order for SB-Silanol

Vacuum		Water		Methanol	
**Bond**	**bond order**	**Bond**	**bond order**	**Bond**	**bond order**
1(Si)-2(O)	1.16694022	1(Si)-2(O)	1.15489924	1(Si)-2(O)	1.15524189
1(Si)-3(C)	0.95371994	1(Si)-3(C)	0.95689588	1(Si)-3(C)	0.95678904
1(Si)-7(C)	0.95391811	1(Si)-7(C)	0.95528676	1(Si)-7(C)	0.95526087
1(Si)-11(C)	0.91642559	1(Si)-11(C)	0.91888869	1(Si)-11(C)	0.9188322
1(Si)-13(H)	0.05652049	1(Si)-13(H)	0.05692933	1(Si)-13(H)	0.05693229
1(Si)-14(N)	0.07765552	1(Si)-14(N)	0.07530103	1(Si)-14(N)	0.07529211
2(O)-23(H)	1.1829781	2(O)-23(H)	1.17606529	2(O)-23(H)	1.1763087
3(C)-4(H)	0.96357784	3(C)-4(H)	0.96330324	3(C)-4(H)	0.96330527
3(C)-5(H)	0.95908621	3(C)-5(H)	0.95782882	3(C)-5(H)	0.95788405
3(C)-6(H)	0.95989837	3(C)-6(H)	0.9593161	3(C)-6(H)	0.95932717
7(C)-8(H)	0.96422283	7(C)-8(H)	0.96278427	7(C)-8(H)	0.96285994
7(C)-9(H)	0.96333271	7(C)-9(H)	0.96337433	7(C)-9(H)	0.96335997
7(C)-10(H)	0.9585505	7(C)-10(H)	0.96004558	7(C)-10(H)	0.95997624
11(C)-12(H)	0.93874693	11(C)-12(H)	0.93810819	11(C)-12(H)	0.93810207
11(C)-13(H)	0.94346028	11(C)-13(H)	0.942539	11(C)-13(H)	0.94254349
11(C)-14(N)	1.11009882	11(C)-14(N)	1.11184942	11(C)-14(N)	1.11190026
14(N)-15(H)	1.02382033	14(N)-15(H)	1.02136282	14(N)-15(H)	1.02141735
14(N)-16(C)	1.12209626	14(N)-16(C)	1.12078598	14(N)-16(C)	1.12086365
16(C)-19(C)	1.02084728	16(C)-17(H)	0.92551404	16(C)-17(H)	0.92549294
19(C)-20(H)	0.92084042	16(C)-18(H)	0.924039	16(C)-18(H)	0.92403009
19(C)-21(H)	0.92059746	16(C)-19(C)	1.02220371	16(C)-19(C)	1.02216287

19(C)-22(N)	1.14378292	19(C)-20(H)	0.92064346	19(C)-20(H)	0.92064753
19(C)-24(C)	0.13107594	19(C)-21(H)	0.92075514	19(C)-21(H)	0.92073643
22(N)-24(C)	2.04302307	19(C)-22(N)	1.14144326	19(C)-22(N)	1.14152429
24(C)-25(H)	0.90895511	22(N)-24(C)	2.03943305	22(N)-24(C)	2.03959325
24(C)-26(C)	1.09372838	24(C)-26(C)	1.09854466	24(C)-25(H)	0.90884686
26(C)-27(C)	1.40600875	26(C)-27(C)	1.39950214	24(C)-26(C)	1.09835775
26(C)-28(C)	1.30668036	26(C)-28(C)	1.31123059	26(C)-27(C)	1.39978711
27(C)-29(C)	1.43064223	27(C)-29(C)	1.43751209	26(C)-28(C)	1.31106653
27(C)-30(H)	0.91151551	27(C)-30(H)	0.91283531	27(C)-29(C)	1.43718052
27(C)-32(C)	0.10870605	28(C)-31(C)	1.29495104	27(C)-30(H)	0.9127805
28(C)-31(C)	1.29429921	28(C)-32(C)	0.09897199	28(C)-31(C)	1.29491537
28(C)-35(O)	1.44587412	28(C)-35(O)	1.43528358	28(C)-35(O)	1.43561554
29(C)-32(C)	1.47180623	29(C)-32(C)	1.46792741	29(C)-32(C)	1.46817832
29(C)-33(H)	0.9232673	29(C)-33(H)	0.92112485	29(C)-33(H)	0.92119037
31(C)-32(C)	1.37068447	31(C)-32(C)	1.3708791	31(C)-32(C)	1.37078831
31(C)-37(C)	1.09381509	31(C)-37(C)	1.09834571	31(C)-37(C)	1.09830063
32(C)-34(H)	0.9084927	32(C)-34(H)	0.90615071	32(C)-34(H)	0.90622836
35(O)-36(H)	1.03688856	35(O)-36(H)	1.0369953	35(O)-36(H)	1.03699826
37(C)-38(O)	1.44713321	37(C)-38(O)	1.47217672	37(C)-38(O)	1.47130621
37(C)-40(O)	2.18533942	37(C)-40(O)	2.15053556	37(C)-40(O)	2.1515904
38(O)-39(H)	1.12038491	38(O)-39(H)	1.10650327	38(O)-39(H)	1.10698243

Table S21. Second Order Perturbation Theory Analysis of Fock Matrix in NBO Basis for:

SB-Silanol - vacuum

Donor NBO(i)	Acceptor NBO(j)	E(2)
BD(1)C26-C27	BD*(1)C24-C26	3.16
BD(1)C26-C27	BD*(1)C26-C28	3.9
BD(1)C26-C27	BD*(1)C27-C29	3.1
BD(1)C26-C27	BD*(1)C28-O35	3.1
BD(2)C26-C27	BD*(2)N22-C24	14.53
BD(2)C26-C27	BD*(2)C28-C31	27.22
BD(2)C26-C27	BD*(2)C29-C32	17.25
BD(1)C26-C28	BD*(1)C26-C27	3.97
BD(1)C26-C28	BD*(1)C28-C31	4
BD(1)C27-C29	BD*(1)C24-C26	3.3
BD(1)C27-C29	BD*(1)C26-C27	3.28
BD(1)C27-C29	BD*(1)C29-C32	3.28
BD(1)C27-H30	BD*(1)C26-C28	5.15
BD(1)C27-H30	BD*(1)C29-C32	3.82
BD(1)C28-C31	BD*(1)C26-C28	4.13
BD(1)C28-C31	BD*(1)C31-C32	4.82
BD(2)C28-C31	BD*(2)C26-C27	13.33
BD(2)C28-C31	BD*(2)C29-C32	24.7
BD(2)C28-C31	BD*(2)C37-O40	26.37
BD(1)C29-C32	BD*(1)C27-C29	3.11
BD(1)C29-C32	BD*(1)C31-C32	3.8
BD(1)C29-C32	BD*(1)C31-C37	3.48
BD(2)C29-C32	BD*(2)C26-C27	22.69
BD(2)C29-C32	BD*(2)C28-C31	15.17
BD(1)C29-H33	BD*(1)C26-C27	3.92
BD(1)C29-H33	BD*(1)C31-C32	4.45
BD(1)C31-C32	BD*(1)C28-C31	4.57
BD(1)C31-C32	BD*(1)C28-O35	3.48
BD(1)C31-C32	BD*(1)C29-C32	3.52
BD(1)C32-H34	BD*(1)C27-C29	3.72
BD(1)C32-H34	BD*(1)C28-C31	4.49
BD(1)O35-H36	BD*(1)C26-C28	5.29
BD(1)O38-H39	BD*(1)C37-O40	4.12
CR(2)Si1	LP*(1)Si1	5.13
BD(1)C3-H6	LP*(1)Si1	3.49
CR(1)O40	RY*(1)C37	6.22
LP*(1)Si1	RY*(3)Si1	5.35
BD(1)C7-H10	LP*(1)Si1	3.83
LP(1)N14	BD*(1)C11-H12	6.15
LP(1)N14	BD*(1)C16-H18	7.89
LP(1)N22	RY*(1)C24	5.43

LP(1)N22	BD*(1)C19-H21	5.79
LP(1)N22	BD*(1)C24-H25	11.29
LP(1)O35	RY*(1)C28	3.81
LP(1)O35	BD*(1)C28-C31	7.64
LP(2)O35	BD*(2)C28-C31	40.17
LP(1)O38	BD*(1)C31-C37	6.07
LP(2)O38	BD*(2)C37-O40	43.29
LP(1)O40	RY*(1)C37	12.78
LP(1)O40	BD*(1)C31-C37	4.51
BD(1)C11-H13	BD*(1)N14-C16	3.24
LP(1)O40	BD*(1)O35-H36	4.27
LP(2)O40	BD*(1)C31-C37	12.23
LP(2)O40	BD*(1)O35-H36	19.93
LP(2)O40	BD*(1)C37-O38	28.55
BD*(2)C28-C31	BD*(2)C26-C27	123.99
BD*(2)C28-C31	BD*(2)C29-C32	216.92
BD*(2)C29-C32	RY*(3)C29	3.1
BD*(2)C29-C32	BD*(2)C26-C27	219.01
BD(1)O2-H23	LP*(1)Si1	26.29
CR(1)O2	LP*(1)Si1	17.56
LP(1)O2	LP*(1)Si1	23.11
LP(1)O2	BD*(1)Si1-C11	4.53
LP(2)O2	RY*(1)Si1	3.36
LP(2)O2	BD*(1)Si1-C3	6.3
LP(2)O2	BD*(1)Si1-C7	6.5
LP(3)O2	LP*(1)Si1	264.27
LP(3)O2	BD*(1)Si1-C11	5.78
LP(3)O2	BD*(1)O2-H23	3.21
BD(1)C16-C19	BD*(1)C11-N14	3.14
BD(1)C19-H20	BD*(2)N22-C24	3.56
BD(1)C19-N22	BD*(1)C24-C26	5
BD(2)N22-C24	BD*(1)C19-H20	3.7
BD(2)N22-C24	BD*(2)C26-C27	7.33
BD(1)C24-H25	BD*(1)C26-C27	3.77
BD(1)C24-C26	BD*(1)C19-N22	4.53
BD(1)C24-C26	BD*(1)C26-C27	3.27

SB-Silanol - water

Donor NBO(i)	Acceptor NBO(j)	E(2)
BD(1)C24-C26	BD*(1)C26-C27	3.27
BD(1)C24-C26	BD*(1)C28-C31	3
BD(1)C26-C27	BD*(1)C24-C26	3.18
BD(1)C26-C27	BD*(1)C26-C28	3.96
BD(1)C26-C27	BD*(1)C27-C29	3.08
BD(1)C26-C27	BD*(1)C28-O35	3.15
BD(2)C26-C27	BD*(2)N22-C24	15.23
BD(2)C26-C27	BD*(2)C28-C31	27.27
BD(2)C26-C27	BD*(2)C29-C32	16.28
BD(1)C26-C28	BD*(1)C26-C27	4.02
BD(1)C26-C28	BD*(1)C28-C31	4.01
BD(1)C27-C29	BD*(1)C24-C26	3.35
BD(1)C27-C29	BD*(1)C26-C27	3.28
BD(1)C27-C29	BD*(1)C29-C32	3.12
BD(1)C27-H30	BD*(1)C26-C28	5.1
BD(1)C27-H30	BD*(1)C29-C32	3.8
BD(1)C28-C31	BD*(1)C26-C28	4.14
BD(1)C28-C31	BD*(1)C31-C32	4.75
BD(2)C28-C31	BD*(2)C26-C27	13.5
BD(2)C28-C31	BD*(2)C29-C32	24.16
BD(2)C28-C31	BD*(2)C37-O40	27.84
BD(1)C29-C32	BD*(1)C27-C29	3.01
BD(1)C29-C32	BD*(1)C31-C32	3.59
BD(1)C29-C32	BD*(1)C31-C37	3.53
BD(2)C29-C32	BD*(2)C26-C27	24.24
BD(2)C29-C32	BD*(2)C28-C31	15.58
BD(1)C29-H33	BD*(1)C26-C27	4.05
BD(1)C29-H33	BD*(1)C31-C32	4.45
BD(1)C31-C32	BD*(1)C28-C31	4.58
BD(1)C31-C32	BD*(1)C28-O35	3.55
BD(1)C31-C32	BD*(1)C29-C32	3.3
BD(1)C31-C37	BD*(1)C31-C32	3
BD(1)C32-H34	BD*(1)C27-C29	3.8
BD(1)C32-H34	BD*(1)C28-C31	4.63
BD(1)O35-H36	BD*(1)C26-C28	5.25
BD(1)O38-H39	BD*(1)C37-O40	4.19
CR(2)Si1	LP*(1)Si1	5.08
BD(1)C3-H6	LP*(1)Si1	3.5
CR(1)O35	RY*(1)C28	3.02
BD(1)C7-H10	LP*(1)Si1	3.75
CR(1)O40	RY*(1)C37	6.01
LP*(1)Si1	RY*(3)Si1	5.08
LP(1)N14	BD*(1)C11-H12	6.4
LP(1)N14	BD*(1)C16-H18	7.69
LP(1)N22	RY*(1)C24	5.49
LP(1)N22	BD*(1)C19-H21	5.6
LP(1)N22	BD*(1)C24-H25	11.01
LP(1)O35	RY*(1)C28	3.84
LP(1)O35	BD*(1)C28-C31	7.46
LP(2)O35	BD*(2)C28-C31	38.86
LP(1)O38	RY*(2)C37	3.06
LP(1)O38	BD*(1)C31-C37	6.49
LP(2)O38	BD*(2)C37-O40	46.8
LP(1)O40	RY*(1)C37	12.65

LP(1)O40	BD*(1)C31-C37	4.52
LP(1)O40	BD*(1)O35-H36	4.27
LP(2)O40	BD*(1)C31-C37	11.44
LP(2)O40	BD*(1)O35-H36	21.01
BD(1)C11-H13	BD*(1)N14-C16	3.36
LP(2)O40	BD*(1)C37-O38	26.84
BD*(2)C28-C31	BD*(2)C26-C27	137.15
BD*(2)C28-C31	BD*(2)C29-C32	148.83
BD*(2)C37-O40	BD*(2)C28-C31	140.21
BD(1)O2-H23	LP*(1)Si1	27.33
CR(1)O2	LP*(1)Si1	17.25
LP(1)O2	LP*(1)Si1	21.96
LP(1)O2	BD*(1)Si1-C11	4.12
LP(2)O2	RY*(1)Si1	3.3
LP(2)O2	BD*(1)Si1-C3	5.86
LP(2)O2	BD*(1)Si1-C7	6.55
LP(3)O2	LP*(1)Si1	260.17
LP(3)O2	BD*(1)Si1-C11	5.89
LP(3)O2	BD*(1)O2-H23	3.41
BD(1)C16-C19	BD*(1)C11-N14	3.1
BD(1)C19-H20	BD*(2)N22-C24	3.51
BD(1)C19-N22	BD*(1)C24-C26	5.08
BD(2)N22-C24	BD*(1)C19-H20	3.65
BD(2)N22-C24	BD*(2)C26-C27	7.08
BD(1)C24-H25	BD*(1)C26-C27	3.82
BD(1)C24-C26	BD*(1)C19-N22	4.44

SB-Silanol - methanol

Donor NBO(i)	Acceptor NBO(j)	E(2)
BD(1)C24-C26	BD*(1)C26-C27	3.27
BD(1)C24-C26	BD*(1)C28-C31	3
BD(1)C26-C27	BD*(1)C24-C26	3.18
BD(1)C26-C27	BD*(1)C26-C28	3.96
BD(1)C26-C27	BD*(1)C27-C29	3.08
BD(1)C26-C27	BD*(1)C28-O35	3.15
BD(2)C26-C27	BD*(2)N22-C24	15.21
BD(2)C26-C27	BD*(2)C28-C31	27.28
BD(2)C26-C27	BD*(2)C29-C32	16.31
BD(1)C26-C28	BD*(1)C26-C27	4.01
BD(1)C26-C28	BD*(1)C28-C31	4.01
BD(1)C27-C29	BD*(1)C24-C26	3.35
BD(1)C27-C29	BD*(1)C26-C27	3.28
BD(1)C27-C29	BD*(1)C29-C32	3.12
BD(1)C27-H30	BD*(1)C26-C28	5.1
BD(1)C27-H30	BD*(1)C29-C32	3.8
BD(1)C28-C31	BD*(1)C26-C28	4.14
BD(1)C28-C31	BD*(1)C31-C32	4.76
BD(2)C28-C31	BD*(2)C26-C27	13.49
BD(2)C28-C31	BD*(2)C29-C32	24.17
BD(2)C28-C31	BD*(2)C37-O40	27.81
BD(1)C29-C32	BD*(1)C27-C29	3.02
BD(1)C29-C32	BD*(1)C31-C32	3.6
BD(1)C29-C32	BD*(1)C31-C37	3.53
BD(2)C29-C32	BD*(2)C26-C27	24.18
BD(2)C29-C32	BD*(2)C28-C31	15.56
BD(1)C29-H33	BD*(1)C26-C27	4.04
BD(1)C29-H33	BD*(1)C31-C32	4.45
BD(1)C31-C32	BD*(1)C28-C31	4.58
BD(1)C31-C32	BD*(1)C28-O35	3.55
BD(1)C31-C32	BD*(1)C29-C32	3.3
BD(1)C31-C37	BD*(1)C31-C32	3
BD(1)C32-H34	BD*(1)C27-C29	3.79
BD(1)C32-H34	BD*(1)C28-C31	4.62
BD(1)O35-H36	BD*(1)C26-C28	5.25
BD(1)O38-H39	BD*(1)C37-O40	4.19
CR(2)Si1	LP*(1)Si1	5.08
BD(1)C3-H6	LP*(1)Si1	3.5
CR(1)O35	RY*(1)C28	3.02
BD(1)C7-H10	LP*(1)Si1	3.75
CR(1)O40	RY*(1)C37	6.01
LP*(1)Si1	RY*(3)Si1	5.09
LP(1)N14	BD*(1)C11-H12	6.4
LP(1)N14	BD*(1)C16-H18	7.7
LP(1)N22	RY*(1)C24	5.49
LP(1)N22	BD*(1)C19-H21	5.6
LP(1)N22	BD*(1)C24-H25	11.02
LP(1)O35	RY*(1)C28	3.84
LP(1)O35	BD*(1)C28-C31	7.47
LP(2)O35	BD*(2)C28-C31	38.9
LP(1)O38	RY*(2)C37	3.05
LP(1)O38	BD*(1)C31-C37	6.48
LP(2)O38	BD*(2)C37-O40	46.72
LP(1)O40	RY*(1)C37	12.65
LP(1)O40	BD*(1)C31-C37	4.52
LP(1)O40	BD*(1)O35-H36	4.27
LP(2)O40	BD*(1)C31-C37	11.46
LP(2)O40	BD*(1)O35-H36	20.98
BD(1)C11-H13	BD*(1)N14-C16	3.36
LP(2)O40	BD*(1)C37-O38	26.89
BD*(2)C28-C31	BD*(2)C26-C27	136.38
BD*(2)C28-C31	BD*(2)C29-C32	150.35
BD*(2)C37-O40	BD*(2)C28-C31	141.23
BD(1)O2-H23	LP*(1)Si1	27.29

CR(1)O2	LP*(1)Si1	17.27
LP(1)O2	LP*(1)Si1	21.99
LP(1)O2	BD*(1)Si1-C11	4.13
LP(2)O2	RY*(1)Si1	3.3
LP(2)O2	BD*(1)Si1-C3	5.86
LP(2)O2	BD*(1)Si1-C7	6.55
LP(3)O2	LP*(1)Si1	260.31
LP(3)O2	BD*(1)Si1-C11	5.88
LP(3)O2	BD*(1)O2-H23	3.4
BD(1)C16-C19	BD*(1)C11-N14	3.1
BD(1)C19-H20	BD*(2)N22-C24	3.51
BD(1)C19-N22	BD*(1)C24-C26	5.08
BD(2)N22-C24	BD*(1)C19-H20	3.65
BD(2)N22-C24	BD*(2)C26-C27	7.09
BD(1)C24-H25	BD*(1)C26-C27	3.82
BD(1)C24-C26	BD*(1)C19-N22	4.44

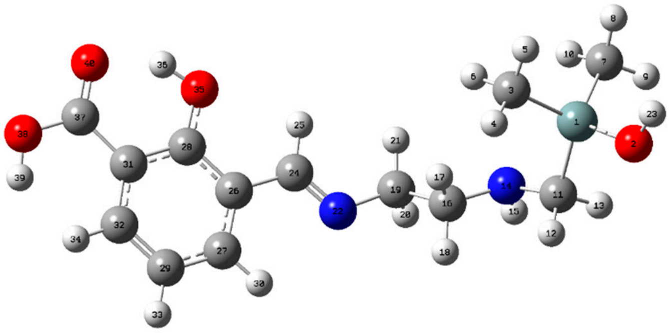

Figure S10. SB-Silanol – atoms labels

Table S22. Wiberg bond order for SB-H-Silanol

Vacuum		Water		Methanol	
Bond	bond order	Bond	bond order	Bond	bond order
1(Si)-11(C)	0.85409934	1(Si)-2(O)	1.18106349	1(C)-2(C)	1.46286659
1(Si)-2(O)	1.22070301	1(Si)-3(C)	0.97312186	1(C)-6(C)	1.37513636
1(Si)-3(C)	0.98329413	1(Si)-7(C)	0.97159763	1(C)-38(H)	0.90606456
1(Si)-7(C)	0.96662316	1(Si)-11(C)	0.874969	2(C)-3(C)	1.44217499
2(O)-23(H)	1.16005256	2(O)-23(H)	1.16542443	2(C)-37(H)	0.92078372
3(C)-4(H)	0.95590281	3(C)-4(H)	0.95949395	3(C)-4(C)	1.3934664
3(C)-5(H)	0.95091875	3(C)-5(H)	0.95341024	3(C)-36(H)	0.91289121
3(C)-6(H)	0.95366183	3(C)-6(H)	0.95493259	4(C)-5(C)	1.30828258
7(C)-10(H)	0.95782221	7(C)-8(H)	0.95428853	4(C)-7(C)	1.10565931
7(C)-8(H)	0.94747271	7(C)-9(H)	0.96015127	5(C)-6(C)	1.29318552
7(C)-9(H)	0.95823066	7(C)-10(H)	0.95688201	5(C)-15(O)	1.44093315
11(C)-12(H)	0.94919571	11(C)-12(H)	0.94511773	6(C)-16(C)	1.09571346
11(C)-13(H)	0.94243719	11(C)-13(H)	0.94075388	7(C)-8(N)	2.02335443
11(C)-14(N)	1.0034048	11(C)-14(N)	1.00747889	7(C)-35(H)	0.90847003
14(N)-15(H)	0.94568111	14(N)-15(H)	0.93801468	8(N)-9(C)	1.14415298
14(N)-16(C)	0.98645362	14(N)-16(C)	0.99448828	9(C)-10(C)	1.02788076
14(N)-41(H)	0.9451119	14(N)-41(H)	0.94146436	9(C)-32(H)	0.91913688
16(C)-17(H)	0.92480472	16(C)-17(H)	0.92449622	9(C)-33(H)	0.9184702
16(C)-18(H)	0.9268517	16(C)-18(H)	0.92755291	10(C)-11(N)	0.99427303
16(C)-19(C)	1.02909985	16(C)-19(C)	1.02757029	10(C)-30(H)	0.92452283
19(C)-20(H)	0.91907936	19(C)-20(H)	0.91945926	10(C)-31(H)	0.92752419
19(C)-21(H)	0.91601081	19(C)-21(H)	0.91859749	11(N)-12(C)	1.00719525
19(C)-22(N)	1.16093977	19(C)-22(N)	1.14367082	11(N)-29(H)	0.9382314

22(N)-24(C)	1.98809784	22(N)-24(C)	2.02389122	11(N)-41(H)	0.94162707
24(C)-25(H)	0.90580208	24(C)-25(H)	0.90841909	12(C)-13(Si)	0.87425305
24(C)-26(C)	1.12685551	24(C)-26(C)	1.10540954	12(C)-27(H)	0.94525816
26(C)-27(C)	1.3793229	26(C)-27(C)	1.39381949	12(C)-28(H)	0.94079034
26(C)-28(C)	1.29580328	26(C)-28(C)	1.30842481	13(Si)-14(C)	0.97148082
27(C)-29(C)	1.45522674	27(C)-29(C)	1.44181989	13(Si)-18(O)	1.18234762
27(C)-30(H)	0.91404191	27(C)-30(H)	0.91288332	13(Si)-19(C)	0.97348028
28(C)-31(C)	1.28976997	28(C)-31(C)	1.29326698	14(C)-24(H)	0.95387151
28(C)-35(O)	1.46412067	28(C)-35(O)	1.44042739	14(C)-25(H)	0.96003617
29(C)-32(C)	1.44624734	29(C)-32(C)	1.46333798	14(C)-26(H)	0.95706446
29(C)-33(H)	0.92122559	29(C)-33(H)	0.92075029	15(O)-39(H)	1.03398605
31(C)-32(C)	1.39330992	31(C)-32(C)	1.37456566	16(C)-17(O)	2.15106771
31(C)-37(C)	1.07761516	31(C)-37(C)	1.09637596	16(C)-20(O)	1.47461564
32(C)-34(H)	0.90788674	32(C)-34(H)	0.9059146	18(O)-34(H)	1.1652356
35(O)-36(H)	1.01852786	35(O)-36(H)	1.03414103	19(C)-21(H)	0.95922385
37(C)-38(O)	1.47180887	37(C)-38(O)	1.4747782	19(C)-22(H)	0.95356263
37(C)-40(O)	2.17973784	37(C)-40(O)	2.14984751	19(C)-23(H)	0.95486596
38(O)-39(H)	1.11611837	38(O)-39(H)	1.10613996	20(O)-40(H)	1.10644721

Table S23. Second Order Perturbation Theory Analysis of Fock Matrix in NBO Basis for:

SB-H-Silanol - vacuum

Donor NBO(i)	Acceptor NBO(j)	E(2)
BD(1)C24-C26	BD*(1)C19-N22	4.69
BD(1)C24-C26	BD*(1)C26-C27	3.34
BD(1)C26-C27	BD*(1)C24-C26	3.36
BD(1)C26-C27	BD*(1)C26-C28	4.12
BD(1)C26-C27	BD*(1)C27-C29	3.06
BD(2)C26-C27	LP*(1)C28	66.25
BD(2)C26-C27	BD*(2)N22-C24	19.34
BD(2)C26-C27	BD*(2)C29-C32	15.85
BD(1)C26-C28	BD*(1)C26-C27	4.15
BD(1)C26-C28	BD*(1)C28-C31	3.84
BD(1)C27-C29	BD*(1)C24-C26	3.46
BD(1)C27-C29	BD*(1)C26-C27	3.28
BD(1)C27-C29	BD*(1)C29-C32	3.12
BD(1)C27-H30	BD*(1)C26-C28	5.04
BD(1)C27-H30	BD*(1)C29-C32	3.82
BD(1)C28-C31	BD*(1)C26-C28	4.04
BD(1)C28-C31	BD*(1)C31-C32	4.72
BD(1)C29-C32	BD*(1)C27-C29	3.02
BD(1)C29-C32	BD*(1)C31-C32	3.65
BD(1)C29-C32	BD*(1)C31-C37	3.53
BD(2)C29-C32	LP(1)C31	44.96
BD(2)C29-C32	BD*(2)C26-C27	25.68
BD(1)C29-H33	BD*(1)C26-C27	4.05
BD(1)C29-H33	BD*(1)C31-C32	4.39
BD(1)C31-C32	BD*(1)C28-C31	4.51
BD(1)C31-C32	BD*(1)C28-O35	3.52
BD(1)C31-C32	BD*(1)C29-C32	3.34
BD(1)C31-C37	BD*(1)C31-C32	3.01
BD(1)C32-H34	BD*(1)C27-C29	3.73
BD(1)C32-H34	BD*(1)C28-C31	4.67
BD(1)O35-H36	BD*(1)C26-C28	5.56
BD(2)C37-O40	LP(1)C31	6.21
BD(1)O38-H39	BD*(1)C37-O40	4.25
CR(2)Si1	LP*(1)Si1	5.95
BD(1)C3-H6	LP*(1)Si1	3.99
BD(1)C7-H10	LP*(1)Si1	3.48
CR(1)O35	RY*(1)C28	3.12
CR(1)O40	RY*(1)C37	6.17
LP*(1)Si1	RY*(3)Si1	5.9
LP*(1)Si1	BD*(1)C11-N14	3.76
LP(1)N22	RY*(1)C24	5.27
LP(1)N22	BD*(1)C19-H21	6.34
LP(1)N22	BD*(1)C24-H25	10.18
LP*(1)C28	BD*(2)C26-C27	50.32
LP(1)C31	RY*(3)C32	3.25
LP(1)C31	BD*(2)C29-C32	71.44
LP(1)C31	BD*(2)C37-O40	67.89

BD(1)C11-H13	BD*(1)N14-C16	3.62
LP(1)O35	RY*(1)C28	3.97
LP(1)O35	BD*(1)C28-C31	7.76
LP(2)O35	LP*(1)C28	77.33
LP(1)O38	BD*(1)C31-C37	6.51
LP(2)O38	BD*(2)C37-O40	46.34
LP(1)O40	RY*(1)C37	12.18
LP(1)O40	BD*(1)C31-C37	4.75
LP(1)O40	BD*(1)O35-H36	5.06
LP(2)O40	BD*(1)C31-C37	12.02
LP(2)O40	BD*(1)O35-H36	23.88
LP(2)O40	BD*(1)C37-O38	27.6
BD*(2)N22-C24	BD*(2)C26-C27	92.23
BD*(2)C29-C32	RY*(3)C29	3
BD(1)O2-H23	LP*(1)Si1	29.63
CR(1)O2	LP*(1)Si1	18.83
LP(1)O2	LP*(1)Si1	24.34
LP(1)O2	BD*(1)Si1-C11	4.96
LP(2)O2	RY*(1)Si1	3.91
LP(2)O2	BD*(1)Si1-C3	3.62
LP(2)O2	BD*(1)Si1-C7	9.12
LP(3)O2	LP*(1)Si1	287.31
LP(3)O2	RY*(3)Si1	3.17
LP(3)O2	BD*(1)Si1-C11	6.53
LP(3)O2	BD*(1)O2-H23	4.64
BD(1)C19-N22	BD*(1)C24-C26	4.56
BD(2)N22-C24	BD*(1)C19-H20	4.6
BD(2)N22-C24	BD*(2)C26-C27	6
BD(1)C24-H25	BD*(1)C26-C27	3.94

SB-H-Silanol - water

Donor NBO(i)	Acceptor NBO(j)	E(2)
BD(1)C24-C26	BD*(1)C19-N22	4.56
BD(1)C24-C26	BD*(1)C26-C27	3.28
BD(1)C26-C27	BD*(1)C24-C26	3.21
BD(1)C26-C27	BD*(1)C26-C28	4.02
BD(1)C26-C27	BD*(1)C27-C29	3.05
BD(1)C26-C27	BD*(1)C28-O35	3.11
BD(2)C26-C27	BD*(2)N22-C24	16.44
BD(2)C26-C27	BD*(2)C28-C31	27.46
BD(2)C26-C27	BD*(2)C29-C32	15.93
BD(1)C26-C28	BD*(1)C26-C27	4.07
BD(1)C26-C28	BD*(1)C28-C31	3.96
BD(1)C27-C29	BD*(1)C24-C26	3.39
BD(1)C27-C29	BD*(1)C26-C27	3.26
BD(1)C27-C29	BD*(1)C29-C32	3.09
BD(1)C27-H30	BD*(1)C26-C28	5.09
BD(1)C27-H30	BD*(1)C29-C32	3.81
BD(1)C28-C31	BD*(1)C26-C28	4.11
BD(1)C28-C31	BD*(1)C31-C32	4.74
BD(2)C28-C31	BD*(2)C26-C27	13.42
BD(2)C28-C31	BD*(2)C29-C32	24.43
BD(2)C28-C31	BD*(2)C37-O40	27.75
BD(1)C29-C32	BD*(1)C31-C32	3.56
BD(1)C29-C32	BD*(1)C31-C37	3.54
BD(2)C29-C32	BD*(2)C26-C27	24.92
BD(2)C29-C32	BD*(2)C28-C31	15.58
BD(1)C29-H33	BD*(1)C26-C27	4.06
BD(1)C29-H33	BD*(1)C31-C32	4.44
BD(1)C31-C32	BD*(1)C28-C31	4.57
BD(1)C31-C32	BD*(1)C28-O35	3.56
BD(1)C31-C32	BD*(1)C29-C32	3.26
BD(1)C31-C37	BD*(1)C31-C32	3.02
BD(1)C32-H34	BD*(1)C27-C29	3.8
BD(1)C32-H34	BD*(1)C28-C31	4.65
BD(1)O35-H36	BD*(1)C26-C28	5.31
BD(1)C3-H6	LP*(1)Si1	3.7
BD(1)O38-H39	BD*(1)C37-O40	4.21
CR(2)Si1	LP*(1)Si1	5.53
BD(1)C7-H10	LP*(1)Si1	3.75
CR(1)O35	RY*(1)C28	3.05
CR(1)O40	RY*(1)C37	6
LP*(1)Si1	RY*(3)Si1	5.21
BD(1)C11-H13	BD*(1)N14-C16	3.68
LP*(1)Si1	BD*(1)C11-N14	4.28
LP(1)N22	RY*(1)C24	5.43
LP(1)N22	BD*(1)C19-H21	5.51
LP(1)N22	BD*(1)C24-H25	10.51
LP(1)O35	RY*(1)C28	3.88
LP(1)O35	BD*(1)C28-C31	7.51
LP(2)O35	BD*(2)C28-C31	39.37
LP(1)O38	RY*(2)C37	3.06
LP(1)O38	BD*(1)C31-C37	6.55
LP(2)O38	BD*(2)C37-O40	47.35
LP(1)O40	RY*(1)C37	12.58
LP(1)O40	BD*(1)C31-C37	4.55
LP(1)O40	BD*(1)O35-H36	4.37
LP(2)O40	BD*(1)C31-C37	11.4
LP(2)O40	BD*(1)O35-H36	21.54
LP(2)O40	BD*(1)C37-O38	26.73
BD*(2)C28-C31	BD*(2)C26-C27	144
BD*(2)C28-C31	BD*(2)C29-C32	145.4
BD*(2)C37-O40	BD*(2)C28-C31	141.92

BD(1)O2-H23	LP*(1)Si1	29.45
CR(1)O2	LP*(1)Si1	17.91
LP(1)O2	LP*(1)Si1	22.62
LP(1)O2	BD*(1)Si1-C11	4.24
LP(2)O2	RY*(1)Si1	3.68
LP(2)O2	BD*(1)Si1-C3	4.36
LP(2)O2	BD*(1)Si1-C7	8.12
LP(3)O2	LP*(1)Si1	274.59
LP(3)O2	BD*(1)Si1-C11	6.34
LP(3)O2	BD*(1)O2-H23	4.07
BD(1)C19-H20	BD*(2)N22-C24	3.26
BD(1)C19-N22	BD*(1)C24-C26	4.9
BD(2)N22-C24	BD*(1)C19-H20	3.85
BD(2)N22-C24	BD*(2)C26-C27	6.63
BD(1)C24-H25	BD*(1)C26-C27	3.85

SB-H-Silanol - methanol

Donor NBO(i)	Acceptor NBO(j)	E(2)
BD(1)C5-C6	BD*(1)C1-C6	4.74
BD(1)C5-C6	BD*(1)C4-C5	4.11
BD(2)C5-C6	BD*(2)C1-C2	24.47
BD(2)C5-C6	BD*(2)C3-C4	13.43
BD(2)C5-C6	BD*(2)C16-O17	27.58
BD(1)C6-C16	BD*(1)C1-C6	3.01
BD(2)C1-C2	BD*(2)C3-C4	24.93
BD(2)C1-C2	BD*(2)C5-C6	15.59
BD(2)C7-N8	BD*(2)C3-C4	6.61
BD(2)C7-N8	BD*(1)C9-H32	3.92
BD(1)C7-H35	BD*(1)C3-C4	3.85
BD(1)N8-C9	BD*(1)C4-C7	4.87
BD(1)C9-H32	BD*(2)C7-N8	3.3
BD(1)C1-C6	BD*(1)C1-C2	3.27
BD(1)C12-H28	BD*(1)C10-N11	3.66
BD(1)C14-H26	LP*(1)Si13	3.71
BD(1)C1-C6	BD*(1)C5-C6	4.57
BD(1)O15-H39	BD*(1)C4-C5	5.32
BD(1)C1-C6	BD*(1)C5-O15	3.55
BD(1)C19-H23	LP*(1)Si13	3.71
BD(1)O20-H40	BD*(1)C16-O17	4.2
BD(1)C1-H38	BD*(1)C2-C3	3.8
CR(2)Si13	LP*(1)Si13	5.54
CR(1)O15	RY*(1)C5	3.05
BD(1)C1-H38	BD*(1)C5-C6	4.66
CR(1)O17	RY*(1)C16	6.01
LP(1)N8	RY*(1)C7	5.43
LP(1)N8	BD*(1)C7-H35	10.51
LP(1)N8	BD*(1)C9-H33	5.53
LP*(1)Si13	RY*(3)Si13	5.24
LP*(1)Si13	BD*(1)N11-C12	4.22
LP(1)O15	RY*(1)C5	3.88
LP(1)O15	BD*(1)C5-C6	7.52
LP(2)O15	BD*(2)C5-C6	39.41
LP(1)O17	RY*(1)C16	12.58
LP(1)O17	BD*(1)C6-C16	4.55
LP(1)O17	BD*(1)O15-H39	4.37
LP(2)O17	BD*(1)C6-C16	11.43
LP(2)O17	BD*(1)O15-H39	21.5
LP(2)O17	BD*(1)C16-O20	26.77
LP(1)O20	RY*(2)C16	3.05
BD(1)C2-C3	BD*(1)C1-C2	3.09
LP(1)O20	BD*(1)C6-C16	6.54
LP(2)O20	BD*(2)C16-O17	47.12
BD*(2)C5-C6	BD*(2)C1-C2	146.6
BD*(2)C5-C6	BD*(2)C3-C4	144.72
BD*(2)C16-O17	BD*(2)C5-C6	149.29
BD(1)C1-C2	BD*(1)C1-C6	3.57
BD(1)C2-C3	BD*(1)C3-C4	3.26
BD(1)C2-C3	BD*(1)C4-C7	3.39
BD(1)O18-H34	LP*(1)Si13	29.51
CR(1)O18	LP*(1)Si13	17.94
LP(1)O18	LP*(1)Si13	22.74
LP(1)O18	BD*(1)C12-Si13	4.22
BD(1)C2-H37	BD*(1)C1-C6	4.44
LP(2)O18	RY*(1)Si13	3.69
LP(2)O18	BD*(1)Si13-C14	8.19
LP(2)O18	BD*(1)Si13-C19	4.25
LP(3)O18	LP*(1)Si13	275.18
BD(1)C2-H37	BD*(1)C3-C4	4.06
LP(3)O18	BD*(1)C12-Si13	6.32
LP(3)O18	BD*(1)O18-H34	4.1
BD(1)C3-C4	BD*(1)C2-C3	3.05
BD(1)C3-C4	BD*(1)C4-C5	4.03
BD(1)C3-C4	BD*(1)C4-C7	3.2
BD(1)C1-C2	BD*(1)C2-C3	3
BD(1)C3-C4	BD*(1)C5-O15	3.11
BD(2)C3-C4	BD*(2)C1-C2	15.94
BD(2)C3-C4	BD*(2)C5-C6	27.46
BD(2)C3-C4	BD*(2)C7-N8	16.49
BD(1)C3-H36	BD*(1)C1-C2	3.81
BD(1)C3-H36	BD*(1)C4-C5	5.09
BD(1)C4-C5	BD*(1)C3-C4	4.07
BD(1)C4-C5	BD*(1)C5-C6	3.96
BD(1)C1-C2	BD*(1)C6-C16	3.54

BD(1)C4-C7	BD*(1)C3-C4	3.27

BD(1)C4-C7	BD*(1)N8-C9	4.58

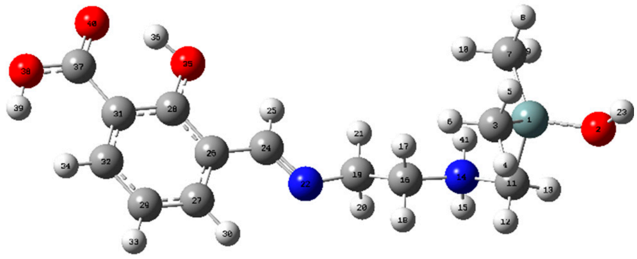

Figure S11. SB-H-Silanol – atoms labels for vacuum and water

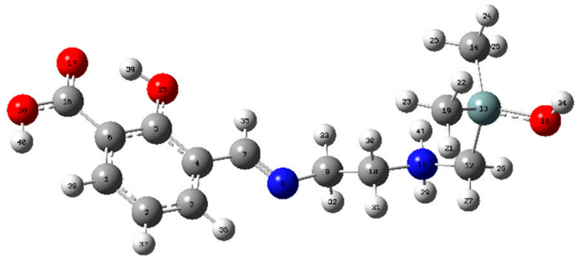

Figure S12. SB-H-Silanol – atoms labels for methanol

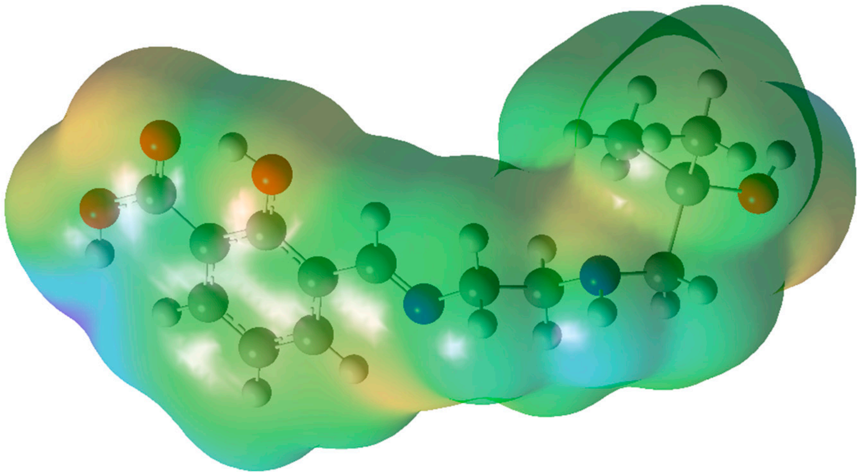

Figure S13. Calculation of the electrostatic potential surface (ESP), which disclosed five possible regions of protonation in the vicinity of the molecule (highlighted by yellow color). From these, one region was attributed to the secondary amino group.

Table S24. Geometry Optimization, XYZ Matrix for:

AEAMDS – Vacuum

Si	-1.64389	0.946078	-0.07534
O	0.000005	0.752967	-0.00012
Si	1.643893	0.946057	0.07535
C	2.07224	2.497606	1.047811
H	3.152653	2.551371	1.209003
H	1.760718	3.404456	0.521153
H	1.588108	2.49581	2.029105
C	2.313054	1.085008	-1.67775
H	3.39679	1.231954	-1.68223
H	2.089916	0.191134	-2.26715
H	1.861085	1.941476	-2.18803
C	2.315771	-0.56659	1.00956
H	1.90625	-0.49206	2.023892
H	1.918575	-1.50996	0.597039
C	-2.31286	1.084669	1.677859
H	-2.08966	0.190675	2.267062
H	-1.86084	1.941035	2.188271
H	-3.39659	1.231611	1.68249
C	-2.07234	2.49783	-1.04743
H	-1.76074	3.404568	-0.52063
H	-1.58833	2.496227	-2.02878
H	-3.15278	2.551641	-1.20848
C	-2.31588	-0.56638	-1.00979

H	-1.91863	-1.50983	-0.59751
H	-1.90647	-0.49163	-2.02415
N	3.791256	-0.56505	1.101288
H	4.069047	-0.95253	1.996813
N	-3.79137	-0.56481	-1.10135
H	-4.06927	-0.9521	-1.99692
C	4.454643	-1.32423	0.045532
H	4.142107	-2.38718	0.033852
H	4.175889	-0.91296	-0.9271
C	5.972896	-1.24582	0.183945
H	6.259692	-1.5898	1.193761
H	6.272394	-0.19678	0.113339
C	-4.45464	-1.32423	-0.04568
H	-4.17577	-0.91317	0.92701
H	-4.1421	-2.38718	-0.03427
C	-5.97291	-1.24579	-0.1839
H	-6.25982	-1.58954	-1.19376
H	-6.2724	-0.19677	-0.11303
N	-6.62029	-1.9898	0.901791
H	-7.62328	-1.83836	0.882343
H	-6.47697	-2.98773	0.777016
N	6.6204	-1.98959	-0.90184
H	7.623395	-1.83816	-0.88224
H	6.477067	-2.98754	-0.77731

There are not exist imaginary frequencies

AEAMDS – Water

Si	-1.55701	0.6486	-0.42466
O	0.001801	0.230075	-0.02117
Si	1.547993	0.495846	0.532505
C	1.503654	0.952747	2.357014
H	2.519179	1.040676	2.754354
H	0.996033	1.909026	2.514186
H	0.97933	0.190879	2.941686
C	2.328052	1.898266	-0.45107
H	3.340554	2.115438	-0.0983
H	2.383658	1.661007	-1.51733
H	1.735631	2.812089	-0.33985
C	2.442479	-1.16562	0.317417
H	1.918165	-1.86831	0.975409
H	2.311113	-1.55888	-0.70536
C	-2.30938	1.672728	0.963734
H	-2.33223	1.119503	1.906923
H	-1.72273	2.583357	1.121722
H	-3.3325	1.975865	0.722771
C	-1.55205	1.649687	-2.01737
H	-1.04685	2.610453	-1.88124
H	-1.04165	1.109199	-2.82024
H	-2.57602	1.85044	-2.34606
C	-2.45675	-0.99767	-0.71903
H	-2.28661	-1.70061	0.114345
H	-1.97035	-1.44835	-1.59185
N	3.864332	-1.12645	0.725184
H	4.116287	-2.03602	1.09927
N	-3.89494	-0.83053	-1.02648
H	-4.17331	-1.56337	-1.6716
C	4.78281	-0.82431	-0.37262
H	4.690888	-1.55235	-1.20048
H	4.533985	0.154037	-0.7916
C	6.229874	-0.79482	0.112684
H	6.462531	-1.75305	0.605413
H	6.330232	-0.01499	0.873051
C	-4.75902	-0.91878	0.150818
H	-4.47854	-0.13781	0.862079
H	-4.63823	-1.88261	0.680193
C	-6.22641	-0.73271	-0.22624
H	-6.49431	-1.47106	-0.99963
H	-6.35175	0.25662	-0.6755
N	-7.07543	-0.80943	0.972579
H	-8.03525	-0.5934	0.719804
H	-7.09286	-1.76798	1.311724
N	7.136282	-0.47692	-1.00135
H	8.081732	-0.36913	-0.64591
H	7.174436	-1.26737	-1.64

There are not exist imaginary frequencies

AEAMDS – Methanol

Si	-1.62613	0.838933	-0.05218
O	0.000125	0.492216	-0.00304
Si	1.626248	0.838543	0.052562
C	1.917825	2.459774	0.96223
H	2.989896	2.644345	1.077775
H	1.487191	3.305087	0.417314
H	1.471947	2.437928	1.96124
C	2.26862	0.97496	-1.71076
H	3.336035	1.213549	-1.72973
H	2.11881	0.044387	-2.26573
H	1.740716	1.772795	-2.24286
C	2.416065	-0.58484	1.030906
H	2.012856	-0.50433	2.047058
H	2.078691	-1.56488	0.65224
C	-2.26418	0.965899	1.713423

H	-2.11278	0.032437	2.263097
H	-1.73524	1.761062	2.24849
H	-3.33162	1.204047	1.736216
C	-1.91987	2.465014	-0.95245
H	-1.48812	3.307422	-0.40394
H	-1.47614	2.448553	-1.95252
H	-2.9922	2.650117	-1.06473
C	-2.41839	-0.57919	-1.03611
H	-2.08021	-1.56124	-0.66342
H	-2.01769	-0.4934	-2.05281
N	3.891063	-0.49198	1.110721
H	4.189324	-0.87078	2.004147
N	-3.89359	-0.48574	-1.11173
H	-4.19411	-0.85931	-2.0066
C	4.583712	-1.23275	0.056458
H	4.313347	-2.30554	0.059164
H	4.281826	-0.84016	-0.91779
C	6.098032	-1.09515	0.190257
H	6.400628	-1.42281	1.198424
H	6.363223	-0.03726	0.10799
C	-4.58371	-1.23254	-0.06007
H	-4.27937	-0.8456	0.915676
H	-4.31347	-2.30532	-0.06965
C	-6.09834	-1.09402	-0.1894
H	-6.40335	-1.41557	-1.19882
H	-6.36327	-0.03664	-0.10012
N	-6.77267	-1.83477	0.887568
H	-7.77181	-1.65691	0.844748
H	-6.66675	-2.83333	0.727107
N	6.774884	-1.82941	-0.88958
H	7.773916	-1.6518	-0.84338
H	6.668606	-2.82891	-0.73538

There are not exist imaginary frequencies

Silanol – Vacuum

Si	-1.70135	0.110146	-0.01906
O	-2.9955	-0.05913	-1.07868
C	-1.14545	1.908371	0.038785
H	-0.84012	2.26928	-0.94721
H	-1.95566	2.55444	0.394032
H	-0.30357	2.041478	0.724363
C	-2.15934	-0.45175	1.716165
H	-2.86493	0.230942	2.199863
H	-2.60928	-1.44878	1.703118
H	-1.25931	-0.50021	2.335947
C	-0.41211	-1.07054	-0.747
H	-0.23913	-0.87198	-1.8179
H	-0.86902	-2.06541	-0.69891
N	0.83926	-1.09226	0.039318
H	1.227048	-2.02948	0.017267
C	1.857905	-0.16117	-0.43746
H	1.459775	0.855784	-0.41352
H	2.145405	-0.35623	-1.48938
C	3.102893	-0.21107	0.444396
H	3.473396	-1.25095	0.484031
H	2.814759	0.058259	1.464031
N	4.09875	0.755692	-0.02973
H	4.875276	0.808667	0.621067
H	4.487162	0.451642	-0.91778
H	-3.78835	0.456517	-0.91928

There are not exist imaginary frequencies

Silanol – Water

Si	-1.70176	0.109676	-0.01904
O	-2.97288	-0.044	-1.11769
C	-1.14824	1.90532	0.07148
H	-0.80726	2.272967	-0.90038
H	-1.97583	2.541781	0.402112
H	-0.33211	2.031454	0.788979
C	-2.2214	-0.46969	1.692451
H	-2.99078	0.181481	2.118549
H	-2.61662	-1.48942	1.662888
H	-1.36288	-0.46208	2.370621
C	-0.40249	-1.0688	-0.73563
H	-0.22192	-0.86401	-1.80457
H	-0.85488	-2.06609	-0.6936
N	0.850426	-1.09884	0.0506
H	1.249225	-2.02995	-0.01778
C	1.857575	-0.14516	-0.41482
H	1.449545	0.867123	-0.35486
H	2.126587	-0.31196	-1.4748
C	3.118862	-0.21567	0.441884
H	3.492242	-1.2528	0.446004
H	2.856188	0.030788	1.47461
N	4.112626	0.760047	-0.03141
H	4.907449	0.765261	0.601073
H	4.481414	0.455643	-0.9289
H	-3.75987	0.481969	-0.94849

There are not exist imaginary frequencies

Silanol – Methanol

Si	-1.7015	0.109686	-0.01902

O	-2.97417	-0.04467	-1.11559
C	-1.14774	1.905411	0.069411
H	-0.80754	2.272175	-0.90305
H	-1.97482	2.542532	0.40012
H	-0.331	2.032164	0.786092
C	-2.21824	-0.46838	1.693818
H	-2.98445	0.184692	2.122735
H	-2.61638	-1.48698	1.665145
H	-1.35744	-0.46355	2.369099
C	-0.40306	-1.06932	-0.73606
H	-0.22292	-0.86507	-1.80515
H	-0.8558	-2.06642	-0.69341
N	0.84988	-1.09904	0.050059
H	1.248184	-2.03046	-0.01655
C	1.857429	-0.14629	-0.41603
H	1.449534	0.866114	-0.35811
H	2.12749	-0.31466	-1.47557
C	3.117845	-0.21523	0.442084
H	3.491476	-1.25235	0.448173
H	2.853749	0.032503	1.47411
N	4.111592	0.760255	-0.03133
H	4.905567	0.767522	0.602142
H	4.481529	0.455474	-0.92818
H	-3.76055	0.482307	-0.94707

There are not exist imaginary frequencies

SB-Silanol – Vacuum

Si	5.266953	0.629496	0.458055
O	6.509157	0.343009	1.553276
C	3.893998	1.622458	1.279232
H	3.498522	1.113684	2.162673
H	4.261584	2.604073	1.59721
H	3.065486	1.798484	0.586924
C	5.88835	1.565674	-1.04996
H	6.154833	2.60053	-0.81348
H	6.768823	1.080622	-1.48136
H	5.109372	1.587506	-1.81771
C	4.745377	-1.11752	-0.05325
H	4.572277	-1.76588	0.820902
H	5.611465	-1.54373	-0.57176
N	3.598991	-1.10417	-0.98891
H	3.711936	-1.85566	-1.66116
C	2.303371	-1.26573	-0.33517
H	2.184723	-0.47601	0.41211
H	2.205813	-2.22402	0.202595
C	1.168137	-1.15248	-1.35751
H	1.318492	-1.9179	-2.13063
H	1.211929	-0.16964	-1.84926
N	-0.11237	-1.41366	-0.72567
H	6.96004	1.094731	1.942658
C	-1.01803	-0.52703	-0.81917
H	-0.86354	0.412074	-1.36135
C	-2.34526	-0.70621	-0.20095
C	-2.65501	-1.86202	0.513384
C	-3.31984	0.310493	-0.32113
C	-3.89907	-2.03583	1.115205
H	-1.88452	-2.61974	0.590202
C	-4.60234	0.131937	0.265051
C	-4.85753	-1.04318	0.989337
H	-4.11433	-2.93388	1.680376
H	-5.81139	-1.18823	1.488356
O	-2.99488	1.418721	-0.99588
H	-3.77048	2.028852	-0.94695
C	-5.60412	1.207139	0.092809
O	-6.86301	1.001176	0.523611
H	-6.97874	0.092757	0.823296
O	-5.34429	2.277966	-0.42996

There are not exist imaginary frequencies

SB-Silanol – water

Si	5.274507	0.617655	0.46852
O	6.595021	0.25218	1.452503
C	3.94859	1.490141	1.477976
H	3.593623	0.86679	2.303519
H	4.344412	2.418787	1.902431
H	3.087921	1.75609	0.857136
C	5.793103	1.716744	-0.96551
H	6.128875	2.697252	-0.61417
H	6.607506	1.263148	-1.5381
H	4.951722	1.875551	-1.64644
C	4.764188	-1.09207	-0.17076
H	4.626057	-1.8048	0.65966
H	5.624041	-1.46025	-0.7416
N	3.595768	-1.05374	-1.07851
H	3.691728	-1.80399	-1.75586
C	2.316846	-1.23125	-0.39233
H	2.204702	-0.44458	0.358877
H	2.251513	-2.19312	0.14385
C	1.156793	-1.13543	-1.38808
H	1.299801	-1.90039	-2.16233
H	1.173013	-0.15324	-1.88174
N	-0.10977	-1.40803	-0.72725

H	7.023709	0.990971	1.894339
C	-1.02652	-0.53009	-0.81481
H	-0.88028	0.403118	-1.36826
C	-2.3481	-0.70714	-0.18559
C	-2.65454	-1.85436	0.547241
C	-3.3289	0.301308	-0.32043
C	-3.89833	-2.02651	1.14817
H	-1.8866	-2.61257	0.639153
C	-4.60991	0.126752	0.27094
C	-4.86321	-1.04092	1.007651
H	-4.11247	-2.91915	1.721421
H	-5.81933	-1.19281	1.496639
O	-3.01187	1.402808	-1.01588
H	-3.79913	1.999758	-0.98369
C	-5.61651	1.193112	0.088109
O	-6.86178	1.019086	0.549721
H	-6.99428	0.138721	0.923171
O	-5.3667	2.250725	-0.47916

There are not exist imaginary frequencies

SB-Silanol – methanol

Si	5.27411	0.617941	0.468622
O	6.59367	0.253365	1.453863
C	3.94713	1.491128	1.476231
H	3.592015	0.868702	2.3024
H	4.341864	2.420628	1.89984
H	3.086684	1.75579	0.854544
C	5.793303	1.715932	-0.96607
H	6.124962	2.698443	-0.61639
H	6.610656	1.263685	-1.53551
H	4.953188	1.870162	-1.6496
C	4.76409	-1.09207	-0.16997
H	4.626312	-1.80457	0.660695
H	5.62408	-1.46016	-0.74065
N	3.595693	-1.05387	-1.07761
H	3.692078	-1.80316	-1.75593
C	2.316739	-1.23227	-0.39191
H	2.204107	-0.44606	0.359699
H	2.251313	-2.19439	0.143821
C	1.156966	-1.13609	-1.38796
H	1.299877	-1.90112	-2.16218
H	1.173867	-0.15391	-1.88166
N	-0.10978	-1.4083	-0.72751
H	7.02229	0.991737	1.896276
C	-1.02629	-0.5302	-0.81532
H	-0.88001	0.40303	-1.36877
C	-2.34783	-0.70715	-0.18598
C	-2.65394	-1.85414	0.547186
C	-3.32873	0.30122	-0.3209
C	-3.89763	-2.02625	1.148429
H	-1.88573	-2.61208	0.639092
C	-4.60957	0.126803	0.270881
C	-4.86255	-1.04076	1.007937
H	-4.11148	-2.91877	1.721999
H	-5.81852	-1.1926	1.497339
O	-3.01189	1.402371	-1.01682
H	-3.79897	1.999526	-0.98472
C	-5.6161	1.19331	0.088191
O	-6.86126	1.019154	0.550694
H	-6.99273	0.138755	0.924226
O	-5.36656	2.250554	-0.47946

There are not exist imaginary frequencies

SB-H-Silanol – Vacuum

Si	5.224964	0.637735	0.586657
O	6.876125	0.523742	0.565725
C	4.497982	0.530302	2.303053
H	4.820947	-0.37737	2.819845
H	4.814088	1.384931	2.909294
H	3.403844	0.54161	2.291934
C	4.5866	2.122661	-0.36457
H	4.887868	3.041557	0.148259
H	5.002465	2.181765	-1.37537
H	3.493314	2.160695	-0.42846
C	4.855256	-0.94877	-0.45691
H	4.791221	-1.8459	0.160734
H	5.701167	-1.075	-1.13641
N	3.606115	-0.92282	-1.31838
H	3.653192	-1.67845	-2.00741
C	2.295886	-1.05094	-0.57555
H	2.240122	-0.22078	0.127578
H	2.332161	-1.98465	-0.01507
C	1.103297	-1.04689	-1.53423
H	1.233899	-1.8514	-2.27196
H	1.056078	-0.09164	-2.08458
N	-0.08247	-1.32063	-0.75455
H	7.439413	1.000467	1.179699
C	-1.07829	-0.53208	-0.90009
H	-1.03673	0.329548	-1.57503
C	-2.34456	-0.7143	-0.19303
C	-2.58064	-1.82759	0.618694
C	-3.3544	0.266347	-0.34208

C	-3.7882	-1.98916	1.284929
H	-1.79207	-2.5641	0.713018
C	-4.60159	0.094914	0.318428
C	-4.78274	-1.03036	1.130625
H	-3.95707	-2.85123	1.916874
H	-5.71143	-1.17294	1.674683
O	-3.09347	1.328364	-1.10419
H	-3.89703	1.913578	-1.07167
C	-5.63897	1.138942	0.117793
O	-6.85946	0.966529	0.639318
H	-6.95912	0.097545	1.043937
O	-5.41603	2.158074	-0.51388
H	3.593569	-0.0545	-1.86083

There are not exist imaginary frequencies

SB-H-Silanol – Water

Si	5.222865	0.558406	0.64398
O	6.826941	0.16666	0.907992
C	4.211342	0.397558	2.209517
H	4.231522	-0.62508	2.594865
H	4.615524	1.059474	2.981847
H	3.167985	0.685094	2.053099
C	5.027909	2.247018	-0.13925
H	5.375825	3.015638	0.557948
H	5.615077	2.335506	-1.05737
H	3.983696	2.47581	-0.37381
C	4.842977	-0.82607	-0.63507
H	4.807529	-1.80009	-0.14552
H	5.675567	-0.84548	-1.34048
N	3.588927	-0.7218	-1.47444
H	3.653774	-1.40681	-2.23179
C	2.297939	-0.94958	-0.73343
H	2.214044	-0.17169	0.022907
H	2.378779	-1.9171	-0.24057
C	1.093648	-0.92108	-1.67709
H	1.216398	-1.69732	-2.44123
H	1.025412	0.050569	-2.18602
N	-0.09123	-1.23501	-0.8978
H	7.27524	0.577667	1.653116
C	-1.07537	-0.42898	-0.97045
H	-1.03914	0.471482	-1.59062
C	-2.32783	-0.65276	-0.23225
C	-2.49721	-1.76726	0.5913
C	-3.38284	0.280237	-0.35267
C	-3.67778	-1.97794	1.296272
H	-1.67508	-2.4678	0.669919

C	-4.60044	0.064058	0.348898
C	-4.71585	-1.06636	1.171694
H	-3.78816	-2.84284	1.937167
H	-5.62086	-1.24873	1.740801
O	-3.19531	1.348985	-1.13762
H	-4.01932	1.893801	-1.08342
C	-5.68781	1.051561	0.18212
O	-6.87603	0.840061	0.761152
H	-6.92006	-0.0124	1.212103
O	-5.55433	2.076542	-0.4769
H	3.55983	0.19	-1.93763

There are not exist imaginary frequencies

SB-H-Silanol – Methanol

C	-4.73784	-1.10928	1.123052
C	-3.69767	-2.02162	1.222706
C	-2.51155	-1.78042	0.537241
C	-2.338	-0.63379	-0.24013
C	-3.39463	0.300435	-0.33374
C	-4.61892	0.051975	0.345113
C	-1.07987	-0.37834	-0.95792
N	-0.09639	-1.18714	-0.91224
C	1.093596	-0.84384	-1.67029
C	2.295677	-0.94031	-0.72865
N	3.593109	-0.72797	-1.46312
C	4.84265	-0.8525	-0.6193
Si	5.249902	0.541128	0.64196
C	5.040707	2.223999	-0.15065
O	-3.20272	1.400356	-1.07292
C	-5.70936	1.04039	0.203471
O	-5.5704	2.094202	-0.40678
O	6.858449	0.15223	0.877555
C	4.267482	0.386784	2.226485
O	-6.90644	0.795608	0.750237
H	4.306825	-0.63226	2.620164
H	4.675765	1.060102	2.986551
H	3.217875	0.659548	2.085589
H	5.402571	2.996352	0.535247
H	5.612788	2.305586	-1.07893
H	3.993403	2.45522	-0.36823
H	4.783477	-1.81959	-0.11856
H	5.676432	-0.89881	-1.32202
H	3.649579	-1.4111	-2.22288
H	2.236031	-0.18673	0.054088
H	2.346523	-1.92616	-0.26914
H	1.208851	-1.57708	-2.47708

H	1.038734	0.155598	-2.12481
H	7.318777	0.559946	1.61708
H	-1.03917	0.547786	-1.53886
H	-1.68805	-2.4813	0.595642
H	-3.81075	-2.9106	1.829282
H	-5.64721	-1.31532	1.677005
H	-4.02805	1.941439	-1.00331
H	-6.95366	-0.07872	1.156805
H	3.578871	0.185982	-1.92258

There are not exist imaginary frequencies

C	4.459511	1.752	-0.75713
H	5.515609	1.866781	-0.46174
H	4.45674	1.456974	-1.81216
N	3.68167	2.992094	-0.6386
H	3.798089	3.386125	0.290772
H	4.021056	3.688619	-1.2937
N	-4.39281	1.941942	1.601738
H	-4.64763	2.788947	2.098792
H	-5.17922	1.304726	1.691023

There are not exist imaginary frequencies

APTMDS – Vacuum

Si	1.498994	-1.02285	0.683972
O	0.009879	-1.30248	0.012585
Si	-1.46533	-1.02237	-0.68897
C	2.421304	-2.66316	0.726866
H	3.409253	-2.55724	1.186071
H	1.86466	-3.40716	1.304257
H	2.561886	-3.06264	-0.28189
C	-1.22875	-0.88033	-2.55302
H	-0.57431	-0.04017	-2.80432
H	-0.77471	-1.78905	-2.95947
H	-2.18165	-0.72669	-3.06944
C	1.267485	-0.37571	2.438522
H	2.227318	-0.22075	2.941412
H	0.730091	0.57722	2.443143
H	0.690824	-1.08474	3.040096
C	-2.57263	-2.49285	-0.29477
H	-2.12151	-3.42296	-0.6528
H	-2.72555	-2.59072	0.784045
H	-3.55622	-2.39821	-0.76549
C	2.413687	0.240209	-0.38231
H	1.809188	1.151901	-0.44545
H	2.461954	-0.15739	-1.40518
C	-2.18448	0.574298	0.019334
H	-1.4551	1.377784	-0.15196
H	-2.26427	0.470339	1.106936
C	-3.55324	1.009885	-0.53152
H	-4.27491	0.185578	-0.45131
H	-3.47858	1.241637	-1.60021
C	-4.14241	2.232532	0.184172
H	-3.42013	3.054671	0.135454
H	-5.04117	2.565905	-0.36071
C	3.830207	0.627831	0.076207
H	3.815473	0.948593	1.126709
H	4.496371	-0.24176	0.036404

APTMDS – Water

Si	1.495529	-1.08217	0.682784
O	0.010282	-1.47558	0.039846
Si	-1.44952	-1.10254	-0.67024
C	2.537993	-2.64854	0.66535
H	3.525791	-2.47213	1.102353
H	2.056173	-3.44411	1.241829
H	2.683674	-3.013	-0.35609
C	-1.20262	-0.97838	-2.53334
H	-0.50758	-0.1724	-2.78753
H	-0.7979	-1.91192	-2.93618
H	-2.14906	-0.77785	-3.04536
C	1.25351	-0.49081	2.454605
H	2.211502	-0.25861	2.930267
H	0.635254	0.411285	2.488815
H	0.760847	-1.25983	3.057483
C	-2.63364	-2.51029	-0.27395
H	-2.24107	-3.46293	-0.64252
H	-2.78724	-2.6038	0.80539
H	-3.61119	-2.35094	-0.73945
C	2.283246	0.269643	-0.37574
H	1.612305	1.135786	-0.38586
H	2.320716	-0.09514	-1.4112
C	-2.07816	0.532485	0.036881
H	-1.31189	1.297305	-0.14931
H	-2.14563	0.432977	1.125762
C	-3.43091	1.03184	-0.50149
H	-4.19818	0.256538	-0.37665
H	-3.36269	1.218256	-1.57917
C	-3.93242	2.31625	0.170472
H	-3.16606	3.093336	0.074821
H	-4.81985	2.676027	-0.3733
C	3.688207	0.733711	0.048658
H	3.681439	1.05068	1.099804
H	4.394814	-0.10182	-0.00994

C	4.246441	1.885482	-0.79651
H	5.300329	2.046998	-0.52092
H	4.236694	1.592722	-1.85221
N	3.425809	3.100327	-0.65892
H	3.543696	3.476412	0.278884
H	3.773838	3.818336	-1.28788
N	-4.18554	2.108281	1.606084
H	-4.43817	2.992595	2.037836
H	-4.99882	1.507217	1.716196

There are not exist imaginary frequencies

APTMDS – Methanol

Si	1.493741	-1.07987	0.682601
O	0.010298	-1.47151	0.03537
Si	-1.45215	-1.10182	-0.6702
C	2.532341	-2.64891	0.675077
H	3.5183	-2.47407	1.116855
H	2.045292	-3.4417	1.250973
H	2.682406	-3.0166	-0.34457
C	-1.21208	-0.9857	-2.53475
H	-0.51801	-0.18077	-2.79488
H	-0.80855	-1.92087	-2.93498
H	-2.16026	-0.78751	-3.04449
C	1.247189	-0.48099	2.451315
H	2.204135	-0.25144	2.930368
H	0.632788	0.423923	2.479913
H	0.748455	-1.24566	3.054739
C	-2.63512	-2.50771	-0.26385
H	-2.2426	-3.46218	-0.6277
H	-2.78651	-2.59533	0.816298
H	-3.61368	-2.35166	-0.72835
C	2.289195	0.265577	-0.37823
H	1.620347	1.133234	-0.39557
H	2.330752	-0.10396	-1.41186
C	-2.07828	0.536329	0.031859
H	-1.31372	1.30085	-0.16239
H	-2.14002	0.442701	1.121595
C	-3.43432	1.03176	-0.50181
H	-4.1999	0.25584	-0.37042
H	-3.37167	1.214424	-1.5805
C	-3.93436	2.317762	0.168243
H	-3.16929	3.095342	0.0667
H	-4.82464	2.674867	-0.37276
C	3.69313	0.7286	0.050622
H	3.682508	1.048807	1.100777
H	4.398666	-0.10824	-0.00246

C	4.256596	1.877069	-0.79559
H	5.309833	2.037408	-0.51663
H	4.250127	1.581462	-1.8505
N	3.437423	3.093417	-0.66418
H	3.552316	3.472173	0.272875
H	3.787715	3.809091	-1.29447
N	-4.18055	2.11384	1.605499
H	-4.4304	2.99922	2.036564
H	-4.99295	1.512811	1.721677

There are not exist imaginary frequencies

ClMTMDS – Vacuum

Si	1.21899	-1.07308	-0.2156
O	0.000021	0.000044	-0.5142
Si	-1.219	1.073105	-0.21558
C	-1.17472	1.671906	1.557791
H	-0.22136	2.162043	1.773904
H	-1.97433	2.394965	1.746665
H	-1.29918	0.845328	2.261574
C	-1.08781	2.493156	-1.43474
H	-1.12422	2.137188	-2.46807
H	-1.8963	3.217682	-1.29754
H	-0.13995	3.021601	-1.29765
C	1.087692	-2.49312	-1.43476
H	0.139906	-3.02165	-1.2975
H	1.123854	-2.13711	-2.46809
H	1.89627	-3.21758	-1.29775
C	1.174689	-1.67188	1.557777
H	1.299163	-0.84529	2.261551
H	0.221323	-2.162	1.77389
H	1.974288	-2.39495	1.746665
C	2.86149	-0.17717	-0.56296
H	3.724733	-0.82558	-0.40688
H	2.904915	0.205625	-1.58295
C	-2.86147	0.17713	-0.56295
H	-3.72473	0.825508	-0.40687
H	-2.90487	-0.20567	-1.58293
Cl	-3.11136	-1.26898	0.528567
Cl	3.111433	1.26892	0.528553

There are not exist imaginary frequencies

ClMTMDS – water

Si	1.503125	-0.61766	-0.61598
O	-0.00011	-0.30222	-0.00082
Si	-1.50266	-0.6175	0.616063
C	-2.39946	-1.87238	-0.44584

H	-1.8671	-2.828	-0.44085
H	-3.40975	-2.04905	-0.06446
H	-2.48156	-1.53541	-1.48224
C	-1.36882	-1.18113	2.399612
H	-0.85053	-0.43954	3.013554
H	-2.35922	-1.34952	2.832893
H	-0.81193	-2.12072	2.461938
C	1.370954	-1.18257	-2.39924
H	0.814114	-2.12219	-2.46145
H	0.853236	-0.44139	-3.01417
H	2.361759	-1.35125	-2.83149
C	2.399179	-1.87153	0.447734
H	2.480537	-1.53354	1.483861
H	1.866783	-2.82714	0.443302
H	3.409728	-2.0486	0.067257
C	2.446715	1.03382	-0.62896
H	3.448683	0.923284	-1.04337
H	1.920886	1.798139	-1.20024
C	-2.44661	1.033778	0.628571
H	-3.44822	0.923336	1.043855
H	-1.92051	1.798678	1.198822
Cl	-2.67934	1.739077	-1.05249
Cl	2.67791	1.740514	1.051712
Cl	2.67791	1.740514	1.051712

There are not exist imaginary frequencies

ClMTMDS – methanol

Si	1.49367	-0.6019	-0.64892
O	-0.000067	-0.3157	-0.0005
Si	-1.493331	-0.60163	0.649129
C	-2.407909	-1.89373	-0.35138
H	-1.881443	-2.85181	-0.31153
H	-3.415911	-2.0482	0.045332
H	-2.49716	-1.59925	-1.40005
C	-1.333664	-1.09984	2.449957
H	-0.807908	-0.3364	3.029774
H	-2.317576	-1.25398	2.902875
H	-0.774227	-2.03592	2.537758
C	1.335139	-1.10158	-2.44944
H	0.775844	-2.03778	-2.53682
H	0.809658	-0.33865	-3.03018
H	2.319338	-1.25598	-2.90164
C	2.407874	-1.89304	0.353174
H	2.496467	-1.5977	1.401656
H	1.881593	-2.85124	0.313793
H	3.416133	-2.04766	-0.04283
C	2.43463	1.050755	-0.61326
H	3.428304	0.960757	-1.05196
H	1.895562	1.836445	-1.14159
C	-2.434604	1.050831	0.612678
H	-3.42801	0.961001	1.052019
H	-1.895381	1.837049	1.140065
Cl	-2.698704	1.686208	-1.09114
Cl	2.697629	1.687556	1.090194

There are not exist imaginary frequencie
